# Phosphanylidenes for effective optical tuning of aromatic hydrocarbons

**DOI:** 10.1039/d6qi01172a

**Published:** 2026-07-21

**Authors:** Lisa N. Kreimer, Peter Coburger, Terrance J. Hadlington

**Affiliations:** a Fakultat für Chemie, Technische Universität München Lichtenberg Strasse 4 85747 Garching Germany terrance.hadlington@tum.de

## Abstract

The development of novel concepts in photophysical tuning of organic molecules remains of significant importance. Here we demonstrate that simple carbene-stabilised phosphanylidene moieties, *i.e.* [NHC·P], drive large bathochromic shifts when bound to conjugated aromatic systems. Modulating the size of the conjugated scaffold from phenyl (2) to anthracenyl (5, 6) leads to the isolation of simple systems demonstrating absorption wavelengths of up to 650 nm. Simultaneously, increasing the number of [NHC·P] units in a given system significantly enhances molar absorptivities, reaching values of up to 16 000 L mol^−1^ cm^−1^. Computational analyses suggest that these photophysical characteristics arise from allowed π to π* transitions, that is P-centred lone pair donation into the aromatic π* orbital, *i.e.* [NHC·P] behaves as strong M+ donor, and effectively extends the conjugated π-system.

## Abstract 2

Carbene-stabilised phosphorous(i) centres are found to effectively extend the π-systems of aromatic hydrocarbons. Through sequential tuning, this leads to strongly red-shifted absorptions with molar absorptivities of up to 16 000 L mol^−1^ cm^−1^.

## Graphical abstract

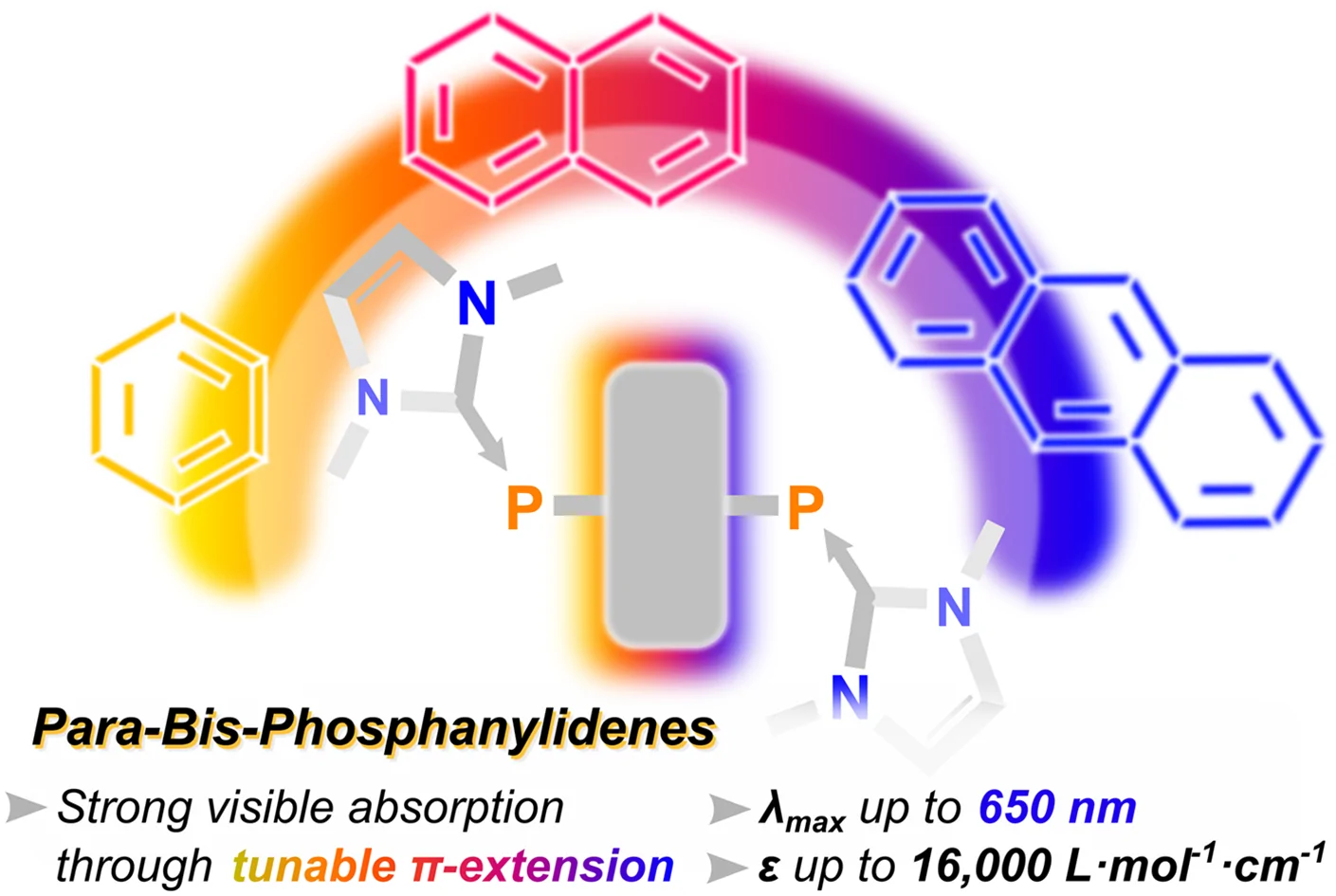

## Introduction

**Fig. 1:**
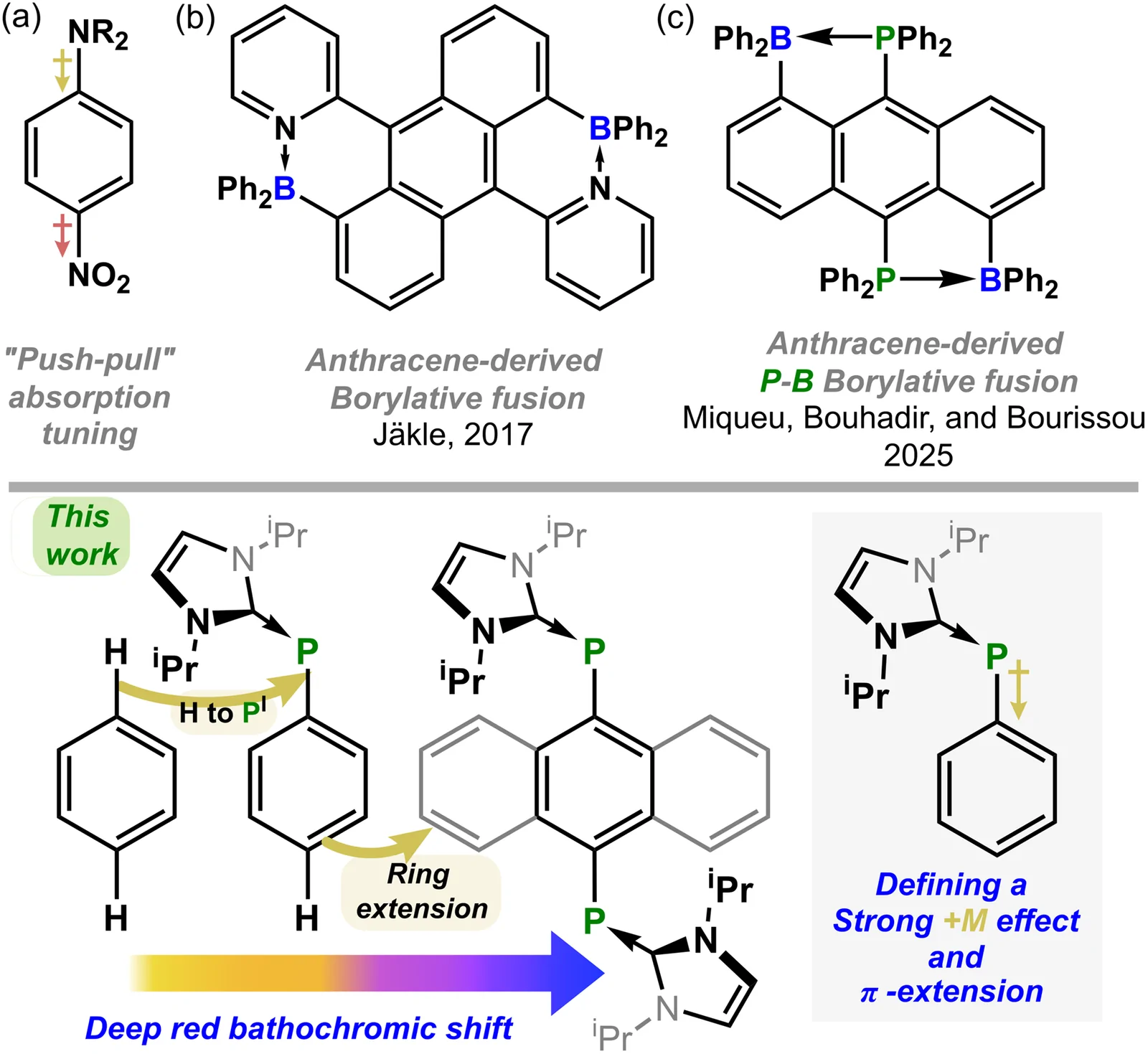
Select methods for optical tuning of aromatic hydrocarbons: “push–pull”, and B–N/B–P borylative fusion methods ((a)–(c)), and this work, defining a strong +M effect and π-extension in aryl phosphanylidenes.

Organic π-conjugated materials have long attracted considerable interest owing to their tunable structural, chemical, and electronic properties.^1,2^ This versatility has enabled the development of tailored functional materials for a wide range of applications including organic light-emitting diodes,^3,4^ organic batteries,^5,6^ and organic photovoltaics;^7,8^ this naturally holds benefits over metal-containing derivatives, regarding sustainability, cost, and safety. A well-established strategy to modulate the properties of such photo-responsive systems is the incorporation of heteroatoms,^9^ such as B,^10–12^ N,^13,14^ Si,^15,16^ P,^17–19^ S,^20,21^*etc*. into the otherwise typically carbon-based frameworks.^22,23^ One prominent example is so-called borylative fusion, describing the N → B Lewis pair fusion within aromatic systems, which has emerged as a key strategy for structural rigidification, thereby extending π-conjugation and modulating the electronic properties of organic π-conjugated materials such as PAHs (Polycyclic Aromatic Hydrocarbons; Fig. 1).^24–26^ This is similar to the well-known +M effect, which also induces a bathochromic shift of the lowest electronic transition in conjugated systems, through donation of e^−^-density, contracting the S_0_–S_1_ energy separation – utilising “push–pull” motifs (Fig. 1(a)) further narrows this gap.^27^ More recently, Le Gac *et al.* reported functionalised pyrenes and anthracenes in which they replaced nitrogen with phosphorus to geometrically enforce strong P → B interactions (Fig. 1(c)). Interestingly, in contrast to N → B systems, these P → B systems do not formally extend the π-systems of PAHs. Still, they demonstrated that the photophysical properties are significantly impacted through a substantial energetic lowering of the first excited state, thereby highlighting the potential of P-containing π-conjugated systems in modulating the photophysical properties of conjugated hydrocarbons.^28^

The π-conjugation abilities of aryl-phosphines compared to their lighter nitrogen congeners were already investigated in 1981 by Cabelli *et al.*^29^ Even though nitrogen more readily adopts a trigonal planar geometry, which facilitates overlap of its lone pair with the neighboring carbon p-orbitals due to the low inversion barriers of amines (*ca*. 2–5 kcal mol^−1^) compared to phosphines (*ca*. 30–35 kcal mol^−1^), the investigation of P-containing chromophores remains of interest as they are expected to exhibit rather different electronic properties, for example in having intrinsically energetically lowered first excited states relative to N-based congeners.^19,30^ A key demonstration of this is found in low-valent phosphorus species such as diphosphenes and phosphanylidenes. The former compound class, for example, show π–π* absorptions at ∼460 nm,^31^ whilst that for azobenzene lies in the UV region.^32^ Base-stabilised phosphanylidenes, which are inherently electron rich, are known to display intense colouration – Kilian and co-workers,^33^ and more recently our group,^34^ accessed naphthalene-supported phosphanyl-phosphanylidenes which demonstrate deep purple-red colouration (*e.g. λ*_max_ = ∼520 nm). TD-DFT calculations attributed these colours to an extended π-conjugation of the aryl ring caused by the phosphanylidene unit. Moving from phosphine to NHC (N-heterocyclic carbene) stabilisation, it is particularly interesting that even the simple ^^i^Pr^NHC-stabilised phenylphosphanylidene (*viz.*1) displays an intense yellow colour,^35^ indicative of a significant bathochromic shift of its first excited state relative to that for benzene.

Despite these discoveries, the π-conjugation abilities of base-stabilised phosphanylidenes remain largely unexplored. While base-stabilised phosphanylidenes are well known in reactive main-group chemistry,^36–40^ their potential role as building blocks for organic optically active materials has not been systematically investigated. In this contribution, we take steps towards describing the potential utility of NHC-stabilised aryl-phosphanylidenes in this regard. We demonstrate that (i) the number of phosphanylidene units, (ii) the size of the π-conjugated backbone, and (iii) the substitution pattern of the phosphanylidene centers have a significant influence on the absorption properties, ultimately leading to a strongly deep-blue absorbing anthracene derivative, with a *λ*_max_ of 653 nm (*ε* = 14 936 L mol^−1^ cm^−1^). Computational assessment models these observations well, suggesting that the [NHC·P] unit effectively extends the conjugated π-system, behaving as a powerful +M donor. These findings thus provide the first well-defined insights into the extended π-conjugation abilities of phosphanylidenes, and reveal their potential as building blocks for organic π-conjugated materials.

## Results and discussion

### Synthesis and characterisation of *p*-bisphosphanylidenes

**Scheme 1:**
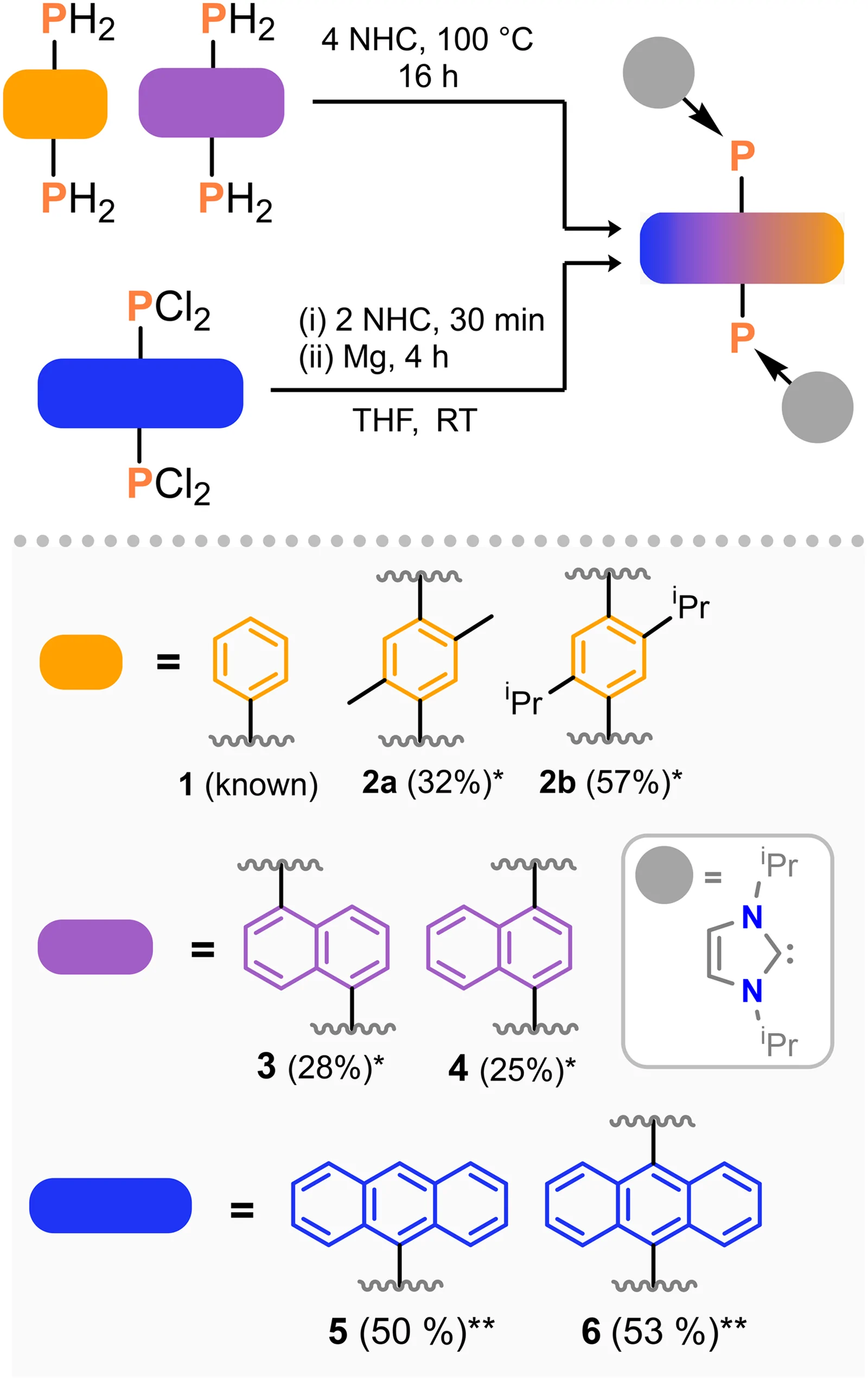
Syntheses of aryl-derived phosphanylidenes featuring phenyl (1, 2a, 2b), naphthyl (3, 4), and anthracenyl (5, 6) backbone scaffolds. Values in parentheses refer to isolated yields; * based on the dibromo-arene starting material (4 reaction steps); ** based on the 9,10-(PCl_2_)_2_Anth starting material (2 reaction steps).

**Fig. 2:**
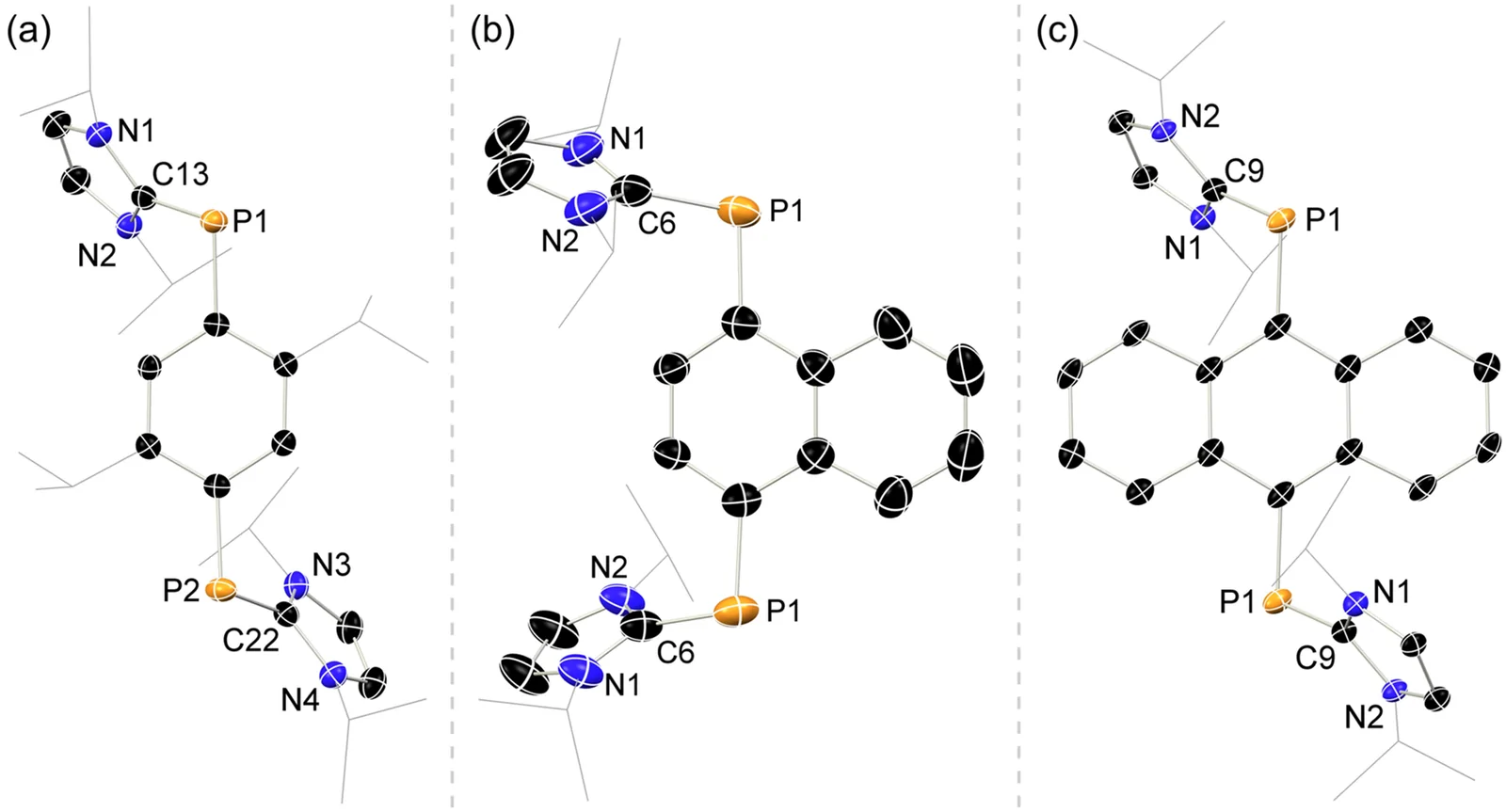
Molecular structures of (a) 2b, (b) 4, and (c) 6, with thermal ellipsoids at 30% probability, and hydrogen atoms omitted for clarity. Selected bond lengths (Å) and angles (°) for 2b: P1–C13 1.790(3); P2–C22 1.785(2); C1–P1–C13 102.1(1); C4–P2–C22 102.4(1). For 4: P1–C6 1.84(1); C4–P1–C6 100.0(6). For 6: P1–C15 1.768(9); P2–C24 1.77(1); C1–P1–C15 102.4(4); C24–P2–C8 104.2(4).

The starting point of this study regards the synthesis of a family of carbene-stabilised aryl-(bis-)phosphanylidenes. Our first approach here was the synthesis of the corresponding aryl-bisphosphines R-(PH_2_)_2_, followed by reduction with the small ^i^Pr-substituted N-heterocyclic carbene ^^i^Pr^NHC (^^i^Pr^NHC = [{(H)CN(^i^Pr)}_2_C:]), in line with our earlier reported xanthene-bound derivatives (Scheme 1).^39^ This allowed for the synthesis of 1,4-bis-(phosphanylidene)benzene derivatives 2a and 2b, and 1,5- and 1,4-bis(phosphanylidene)naphthalene derivatives 3 and 4, as bright orange (2a, 2b) and crimson red (3, 4) solids. In contrast, the anthracene derivative 6 required a modified approach, proceeding most favourably *via* initial ^^i^Pr^NHC addition to the 9,10-bis(dichlorophosphanyl)anthracene, followed by Mg reduction (Scheme 1). This gave access to the deep blue bis-phosphanylidene in reasonable yield of 53%.††All compounds are indefinitely stable under inert atmosphere at ambient temperature. Dilute solutions of 4 (5 × 10^−5^ M) are found to fully decomposed under air after 1 h at 25 °C (Fig. S52). Near complete decomposition of powdered solid-state samples of 4 is observed after 3 h, proceeding with first order decay (Fig. S53–S55). Employing the Ar-PH_2_ reduction route here led to mixtures of 6 with a second species, found to be the 9-phosphanylidene anthracene derivative 5; this blue species can be accessed in its pure form through ^^i^Pr^NHC addition and Mg reduction of the 9-PCl_2_-anthracene.‡‡Attempts to access the mono-phosphanylidene naphthalene derivative of 3 and 4 led to the isolation of mixtures of tetrameric (NapthP)_4_ and the target phosphanylidene, which could not be separated. The X-ray crystal structure of the former is given in the SI (Fig. S53). All synthesised compounds were analyzed by ^31^P NMR spectroscopy, which display singlet resonances between −65 and −85 ppm, characteristic of electron-rich P-centers, and aligning with previously reported carbene-stabilised aryl-phosphanylidenes.^35^ Analysis of the crystal structures of ^^i^Pr^NHC-stabilised aryl-phosphanylidenes 2b, and 4, and 6 reveals C^NHC^–P distances in the range of 1.76–1.84 Å (Fig. 2). These values are significantly elongated compared to classical P=C double bonds in phosphaalkenes,^37^ but in the same range as previously reported C^NHC^–P distances of NHC-stabilised bis-phosphanylidenes.^41–43^ The naphthyl and anthracenyl rings in 4 and 6 maintain planarity but each compound shows bending of the [^^i^Pr^NHC-P] moiety slightly out of the plane, this value increasing with the size of the π-system (*e.g.*2b: 3.96°; 4: 7.30°; 6: 11.09°).

The isolated family of novel mono-and bis-phosphanylidene arenes described above, in addition to the known phenyl phosphanylidene ^^i^Pr^NHC·PPh (*viz*. 1),^35^ forms a strong foundation for investigating the effect of the [^^i^Pr^NHC·P] moiety on the optoelectonic properties of π-conjugated systems. The described colour of the solids already qualitatively indicates a significant red shift on moving from the phenyl group in 1, to the anthracenyl group in 5 and 6. Indeed, in comparison to benzene, compound 1 already shows a significant bathochromic shift in the UV/vis spectrum, solely due to the incorporation of the [^^i^Pr^NHC·P] unit. As a point of reference, the protio-phosphanylidene ^Me^NHC·PH is reportedly colourless (^Me^NHC = [{(H)CN(Me)}_2_C:]).^44^ These observations prompted us to more closely examine the effect of the number and relative position of phosphanylidene units on differing aryl scaffolds on the electronic and optical properties of these compounds. The results are summarised in Table 1.

**Table 1:** Photophysical data for compounds 1–6

	*λ*_tol_ [nm]	*ε*_tol_ [L mol^−1^ cm^−1^]	*λ*_THF_ [nm]	*ε*_THF_ [L mol^−1^ cm^−1^]	*λ*_max em_ [nm]	*Φ* a [%]	*τ* [ns]	
1	346	4000	346	5400				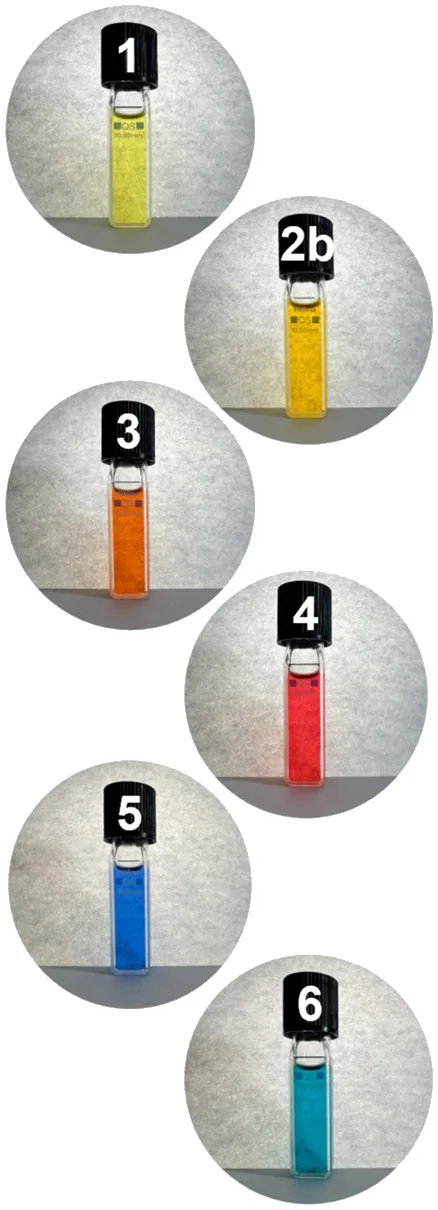
	420	4900	422	6300				
2a	351	9900	346	10 300				
	453	14 100	451	14 900				
2b	351	12 800	352	11 500	370^b^	—	—	
	447	16 900	448	15 900				
3	363	8000	364	6100	372^b^	—	—	
	523	12 900	523	13 400				
4	368	8000	367	8300	435^c^	—	6.0	
	502	15 000	504	15 400				
5	360	11 100	360	9400	432^d^	—	6.3	
	376	12 100	377	10 300				
	395	9400	393	8200				
	604	6600	606	6100				
6	334	13 000	332	14 100	436^e^	2.07	5.9	
	378	14 400	378	15 800	746^f^	0.37	—	
	647	12 100	653	14 900				

a Quantum yields denoted ‘—’ were below the detection limit of the instrument used.

b Excited at 317 nm.

c Excited at 368 nm.

d Excited at 376 nm.

e Excited at 343 nm.

f Excited at 661 nm. Emission data was not collected for 1 or 2a.

**Fig. 3:**
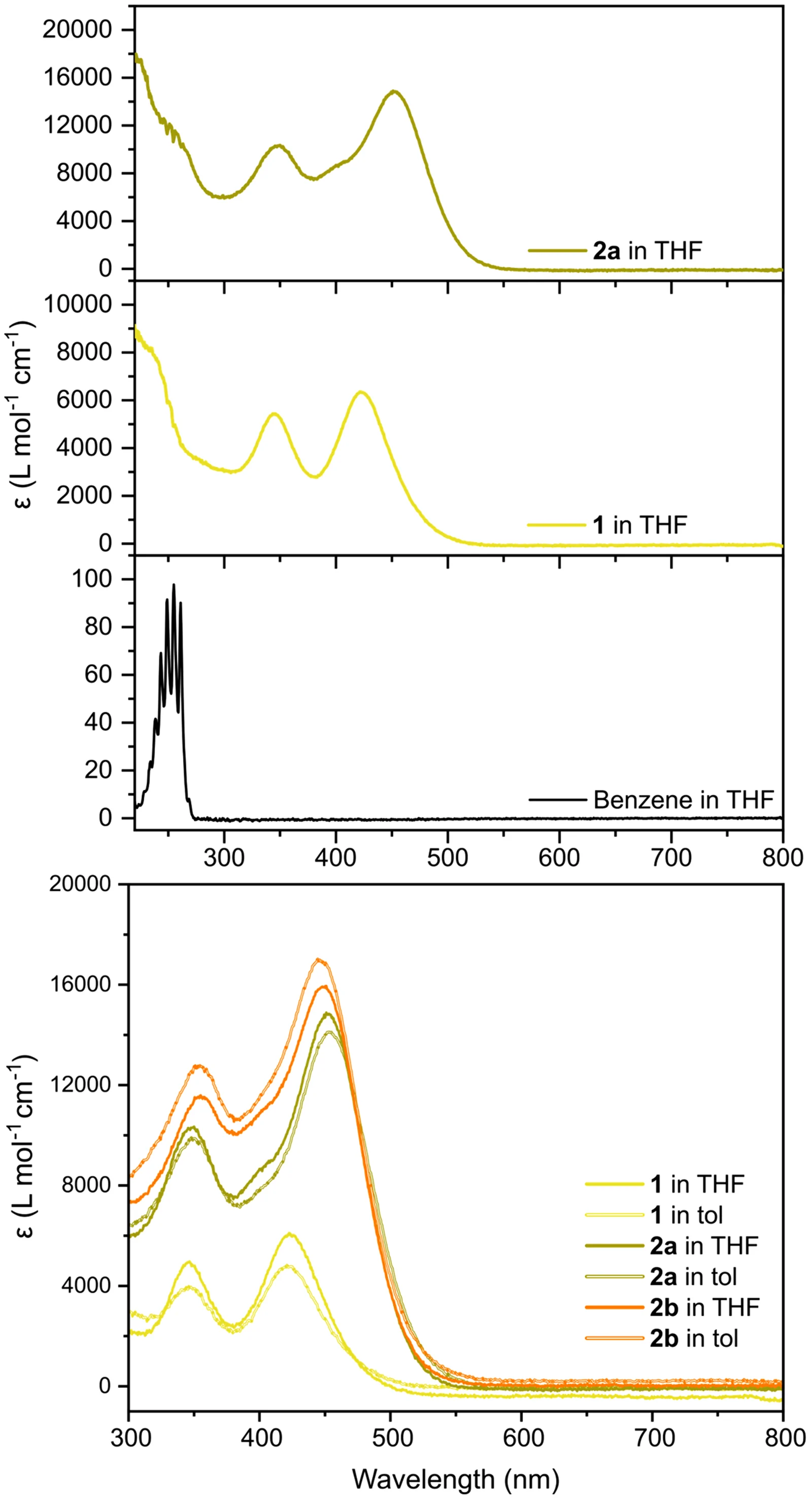
Above: UV/vis spectra in THF of a 1 × 10^−2^ M solution of benzene, a 1 × 10^−4^ M solution of 1, and a 5 × 10^−5^ M solution of 2a; Below: UV/vis spectra of 5 × 10^−5^ M solutions of 1, 2a and 2b in THF and toluene.

We first sought to define the effect of the number of P^I^-centres on the absorption properties of benzene derivatives. The UV/vis spectrum of 1 in THF displays a maximum absorption wavelength (*λ*_max_) at 422 nm with a molar extinction coefficient (*ε*) of 6400 L mol^−1^ cm^−1^ (Fig. 3), confirming the expected bathochromic shift relative to benzene (*λ*_max_ = 255 nm), *i.e.* due to the yellow colouration of crystalline 1. Incorporation of a second [^^i^Pr^NHC·P] moiety induced an additional red-shift of 29 nm, affording a *λ*_max_ of 451 nm for 2a in THF. Though this shift is small, we do observe a remarkable increase of *ε* for 2a, to 14 900 L mol^−1^ cm^−1^. This is greater than an additive effect, being 2.34 times larger and that for 1, emphasising the influence of the number of [^^i^Pr^NHC·P] moieties on the photophysical properties. Similar absorption wavelengths are observed for ^i^Pr-substituted 2b (448 nm, THF), with a somewhat increased molar absorptivity of 15 900 L mol^−1^ cm^−1^. Comparing the UV/vis spectra of 1, 2a, and 2b in THF and toluene (Fig. 3(b)), we find that the spectra do not indicate significant changes in *λ*_max_, indicating that the origin of these absorptions likely relate to π–π* transitions, and not charge-transfer, where one would expect strong solvatochromism.^45^

**Fig. 4:**
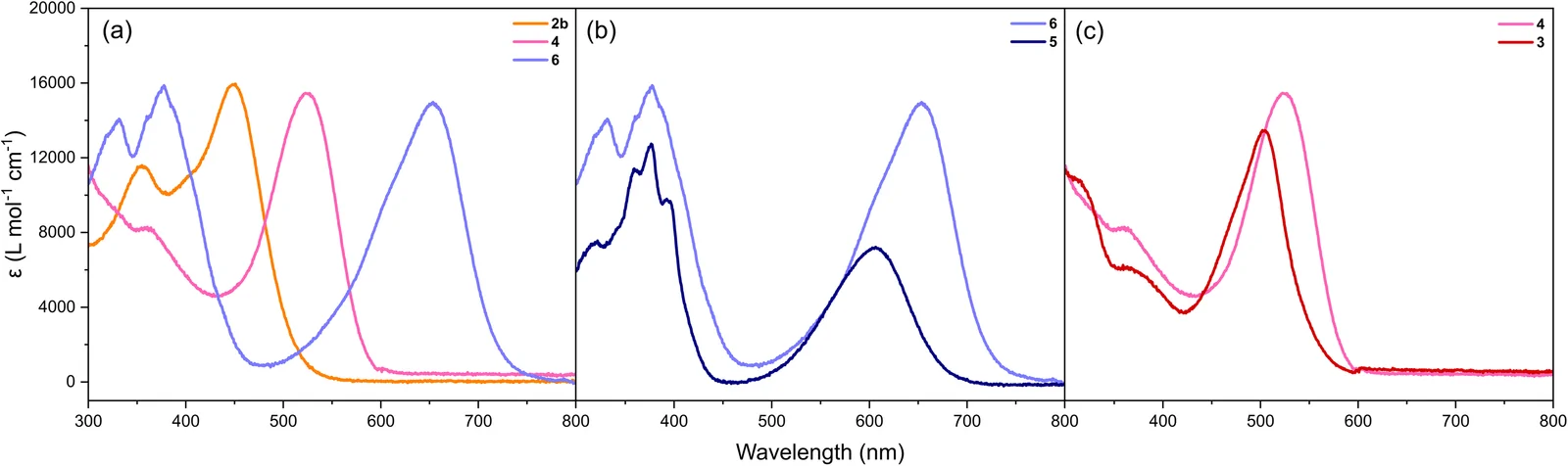
UV/vis spectra of 5 × 10^−5^ M solutions, in THF, of (a) 2b, 4 and 6, (b) 5 and 6, and (c) 3 and 4, demonstrating the effect of π-scaffold extension, additional [^^i^Pr^NHC·P] moieties, and the relative position of [^^i^Pr^NHC·P] substitution, respectively. The molar absorptivity scale given on the left applies for all spectra.

Having established that additional phosphanylidene units induce a red-shift and increase absorption intensity, we next explored expansion of the π-conjugated scaffold which supports the [^^i^Pr^NHC·P] units. Indeed, anthracene itself demonstrates *λ*_max_ values (375 nm, *ε* = ∼7000 L mol^−1^ cm^−1^) red-shifted and of significantly increased intensity relative to benzene (255 nm, ∼200 L mol^−1^ cm^−1^) – we thus anticipated a similar effect in our system. Overlaying the UV/vis spectra of *para*-bis(phosphanylidene)-phenyl (*λ*_max_ = 448 nm), -naphthyl (*λ*_max_ = 523 nm), and -anthracenyl (*λ*_max_ = 653 nm) systems 2b, 4, and 6 (Fig. 4(a)) indicates a significant total bathochromic shift of 231 nm for 6 relative to mono-phosphindene 1. All *para*-bis(phosphanylidene) compounds exhibit high molar absorptivities, up to ∼16 000 L mol^−1^ cm^−1^. In line with the observed increase in *ε* on moving from 1 to 2a/b, the anthracene mono-phosphanylidene 5 demonstrates a value of 6100 L mol^−1^ cm^−1^, some 60% lower than that for 6 (Fig. 4(b)). In this case, *λ*_max_ is also somewhat lower (606 nm), so demonstrating that an increase in [^^i^Pr^NHC·P] moieties increases both *λ*_max_ and *ε*. The strongly red-shifted absorption values for anthracene derivatives 5 and 6 are particularly noteworthy, with such characteristics being of importance in *e.g.* phototheranostics and photocatalysis;^46,47^ whilst observed *ε* values do not compare with the extremely high values of fully conjugated chromophores such as phthalocyanines (*e.g.*, H_2_Pc; *ε* = ∼280 000 L mol^−1^ cm^−1^),^48,49^ they are greater than those in common metal-free blue dyes, *e.g.*, indanthrone blue (*ε* = ∼2000 L mol^−1^ cm^−1^)^50^ and azulene derivatives (∼300–1000 L mol^−1^ cm^−1^).^51^ First, this further suggests that the red-shifted absorptions in our systems arise from allowed π–π* transitions. More broadly, this introduces a novel, and simple-to-install functional group allowing for significant photophysical tuning of conjugated systems.

As a final point of investigation, particularly regarding the origin of the high observed molar absorptivities, we examined the influence of the substitution pattern of the phosphanylidene units. To this end, 1,5- and 1,4-substituted naphthalene derivatives were compared (*viz*. 3 and 4, respectively; Fig. 4(c)). We find that 3 demonstrates a *λ*_max_ value (504 nm) only slightly red-shifted relative to that for 4 (523 nm). However, a more pronounced effect is seen on the magnitude of *ε*: this decreases from 15 500 L mol^−1^ cm^−1^ for 4, to 13 500 L mol^−1^ cm^−1^ for 3, giving some evidence that *para*-substitution is beneficial for maximising molar absorptivity.

We note that the emission behavior of all novel chromophores described here was also investigated *via* fluorescence spectroscopy (see Table S1 in SI). Whilst all compounds exhibit fluorescence in the near-UV and blue visible region (*λ*_em_ = 370–436 nm), only compound 6 shows an additional emission in the far red region (*λ*_em_ = 746 nm). However, this emission is associated with a very low quantum yield (*Φ* = 0.4%), likely due to non-radiative decay pathways, *e.g.* translational quenching through the non-rigid carbene ligands. We thus envisage that further structural modification will lead to enhanced fluorescence efficiency in these systems.

### Computational analysis of optical properties

**Fig. 5:**
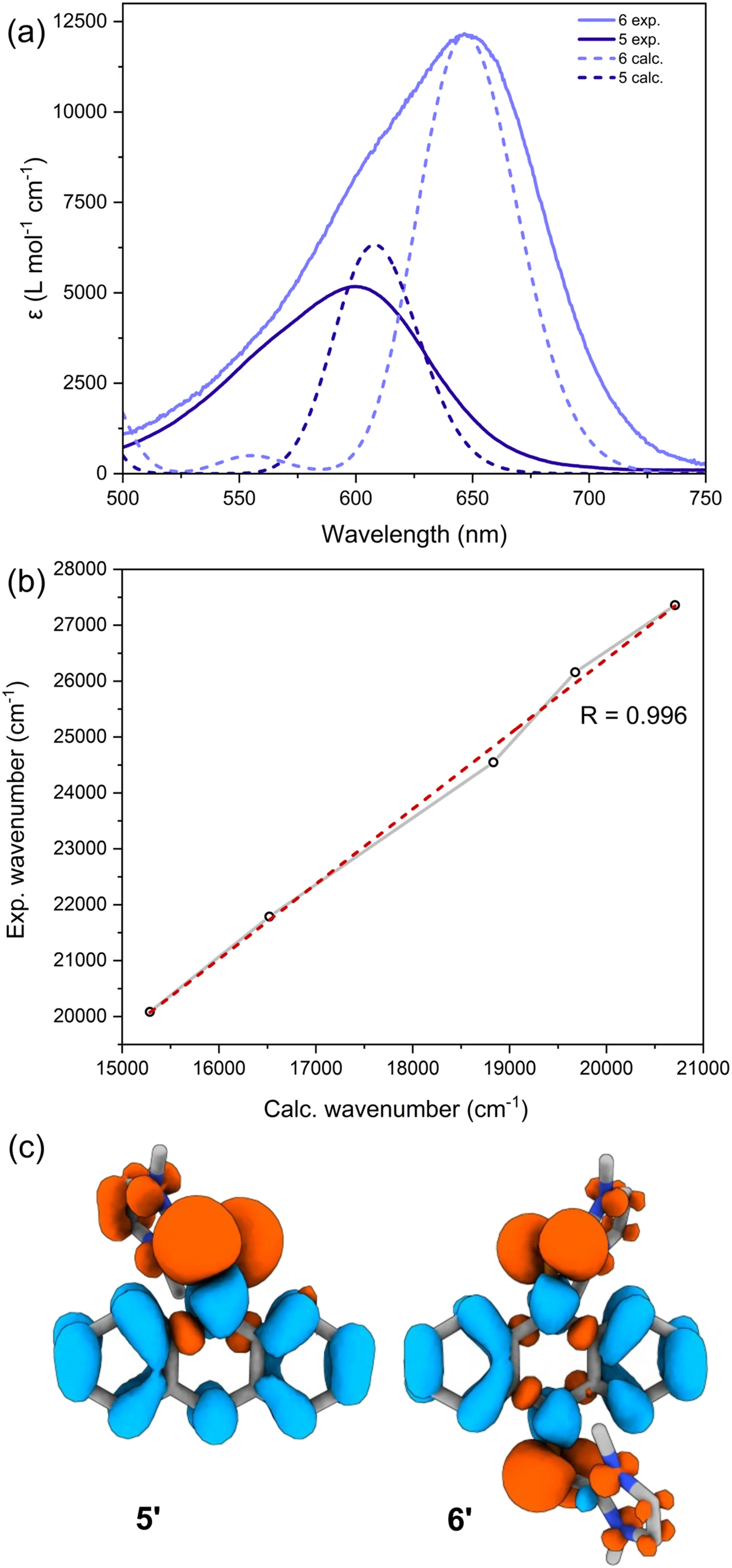
(a) Overlay of the corrected calculated UV-vis spectra for 5′ and 6′ with corresponding experimental spectra (shift and intensity normalised for 6′); (b) plot of the calculated and experimental absorption maxima for all compound pairs; (c) difference density plots for compounds 5′ and 6′; transitions proceed from orange to blue. Surface isovalue = 0.001.

The photophysical properties of compounds 2–6 were further explored using computational methods – this used Density Functional Theory (DFT)-optimised structures 2′–6′ (see SI for details), and a combination of Time-Dependent-DFT (TD-DFT) and Complete Active Space Self-Consistent Field (CASSCF) calculations to define the transitions leading the intense absorption characteristics of bis-phosphanylidene reported herein. Firstly, frontier orbitals for these species (*e.g.* for 5′ and 6′, Fig. S66) suggest significant phosphorous centred p-type lone-pair character for the HOMO, and arene-π* character as the LUMO. To gain deeper insight into the experimental spectra, we performed spectral line fitting with the program Fityk (Fig. S59–S63 in SI).^52^ The strong absorptions observed in the visible region consist of multiple transitions, which can be attributed to vibronic splitting; additional broadening arises from solvent effects. Following the fitting procedure, TD-DFT calculations were conducted. These results qualitatively reproduce the shape of the experimental spectra well (*e.g.*Fig. 5(a); Fig. S64 in SI). In particular, plotting the wavenumbers of the first intense fitted transition against the vertical S_0_–S_1_ transition energies reveals a linear trend (Fig. 5(b)). This trend suggests that, despite a systematic offset in the TDDFT-calculated excitation energies—likely due to the absence of vibronic contributions—the electronic structure and the underlying nature of the transitions are accurately captured by the computational method. Consequently, the TDDFT results can be used to analyze the nature of the observed transitions. In the case of 4′, 5′, and 6′, only the S_0_–S_1_ transitions contribute to the intense absorption bands in the visible region (see Fig. S64). For 3′, S_0_–S_2_ transitions show a notable contribution, whilst for 2′ S_0_–S_3_ transitions also contribute to the maxima in the visible region.

The corresponding difference density plots for all mentioned transitions are shown in Fig. S65 in the SI – that for 5′ and 6′ are given in Fig. 5(c). Aside from the S_0_–S_2_ and S_0_–S_3_ transitions in 2′, all transitions largely comprise π–π* character. That is, an electron is shifted from p-type lone pairs at a P-atom – which show partial overlap with the π-system of the arene in all cases – to the arene π* orbitals. Thus, the phosphanylidene substituents formally represent extensions of the π-system, and suggest a strong +M (mesomeric) effect of the [R–P] substituent. The S_0_–S_2_ and S_0_–S_3_ transitions in 2′ are also π–π* transitions, but show a stronger involvement of the carbene moiety instead of the central arene (see Fig. S65). To quantify the magnitude of the +M effect of the phosphanylidene unit, *i.e.* [^^i^Pr^NHC-P], the localised CASSCF orbtials were analysed (orbital occupations are summarised in Table S4 in SI). The occupation of the p-type lone pairs at the phosphorus atoms significantly deviates from 2.000 in all cases, thus supporting the description as +M substituents. Across all systems, each phosphorus center donates 0.056(15) electrons into the arene system.

## Conclusions

In summary, we report the synthesis of a series of NHC-stabilised aryl-(bis)phosphanylidenes – in this, we demonstrate that their photophysical properties can be easily tuned by (i) varying the number of phosphanylidene units on the aryl core, (ii) the size of the π-conjugated backbone, and (iii) altering the substitution pattern of the phosphanylidene centers. This leads to strongly red-absorbing systems based on the anthracene scaffold, with *λ*_max_ and *ε* values of up to 650 nm and 16 000 L mol^−1^ cm^−1^. DFT calculations suggest that this is caused by the extension of the π-system by these phosphanylidene units, with major S_0_ → S_1_ being P-π to Ar-π* transitions, establishing a strong +M effect for the [NHC·P] fragment. Beyond providing fundamental insights into the electronic nature of these compounds, this contribution establishes NHC-stabilised aryl-phosphanylidenes as a versatile new class of P-containing π-chromophores. Their strong molar absorptivities and tunable UV-vis absorption profiles open new design principles for organic optical materials, bridging main-group element chemistry with functional π-materials research.

## Author contributions

LNK carried out all experimental work and analyses. PC carried out all computational aspects of the work. TJH supervised the experimental aspects of this work, and devised the study. LNK wrote the initial draft of the manuscript, which was edited by all authors.

## Conflicts of interest

There are no conflicts to declare.

## Supplementary Material

QI-013-D6QI01172A-s001

QI-013-D6QI01172A-s002

## Data Availability

The data supporting this article have been included as part of the supplementary information (SI): Details for the synthesis of all novel compounds; analytical data for all novel compounds; printed spectra; summary of X-ray data; details regarding computational studies. See DOI: https://doi.org/10.1039/d6qi01172a. CCDC 2537609–2537614 for compounds 2b, 3, 4, 5, 6 and (NaphthP)_4_ contain the supplementary crystallographic data for this paper.^53*a*–*f*^
