# Supplementary material for: Phosphanylidenes for effective optical tuning of aromatic hydrocarbons
Source: Inorg Chem Front. 2026 Jul 21. Online ahead of print. doi: 10.1039/d6qi01172a (PMC13411660; doi:10.1039/d6qi01172a)
Supplement: QI-OLF-D6QI01172A-s001 [file QI-OLF-D6QI01172A-s001.pdf]

Supplementary Information (SI) for Inorganic Chemistry Frontiers.
This journal is © the Partner Organisations 2026

Phosphanylidenes for effective optical tuning of aromatic hydrocarbons

Lisa N. Kreimer, [a] Peter Coburger,[a] and Terrance J. Hadlington*,[a]

Fakultat für Chemie, Technische Universität München, Lichtenberg Strasse 4, 85747, Garching, Germany. E-mail: terrance.hadlington@tum.de.

1. Experimental methods and data …… 2

General Considerations…… 2

Synthetic details and printed spectra…… 3

Details of fluorescence measurements…… 40

Details on stability measurements…… 41

3. X-ray crystallographic details…… 44

4. Computational methods and details…… 47

5. References…… 52

Experimental methods and data

General considerations. All experiments and manipulations were carried out under dry oxygen free argon atmosphere using standard Schlenk techniques or in a MBraun inert atmosphere glovebox containing an atmosphere of high purity argon. C_6D_6 was dried, degassed by standard procedures and stored over a potassium mirror. All other solvents were degassed by standard procedures and dried over activated 4Å mol sieves. 1,4-dibromo-2,5-(bis)isopropylbenzene,[1] 9-anthryldichlorophosphine,[2] 9,10-bis(dichlorophosphino)anthracene,[2] and 1,3-diisopropylimidazol-2-ylidene (^{iPr}NHC)[3] were synthesized according to known literature procedures. All other reagents were used as received.

NMR spectra were recorded on a Bruker AV 400 Spectrometer. The ^{1}H and ^{13}C{^{1}H} NMR spectra were referenced to the residual solvent signals as internal standards. ^{31}P{^{1}H} NMR spectra were externally calibrated with H_3PO_4. Liquid Injection Field Desorption Ionization Mass Spectrometry (LIFDI-MS) was measured directly from an inert atmosphere glovebox with a Thermo Fisher Scientific Exactive Plus Orbitrap equipped with an ion source from Linden CMS.[4] Elemental analyses (C, H, N) were performed with a combustion analyzer (elementar vario EL, Bruker). Absorption spectra (UV/vis) were recorded in solution on an Agilent Cary 60 UV/vis spectrophotometer with a fiber optic dip probe in the glovebox under inert conditions. Fluorescence emission, excitation and lifetime measurements were recorded in solution on a FS5 spectrofluorometer from Edinburgh Instruments. Quantum yield measurements were performed in solution on a Hamamatsu Quantaurus-QY Absolute PL quantum yield spectrometer.

General synthetic procedure 1 (for primary phosphine synthesis)

The respective $ArBr_n$ (n = 1, 2) was dissolved in THF or Et_2O (10 mL/g). It was cooled to -78 °C and a lithiation reagent (*n*-Buli or *t*-BuLi) was added. The reaction mixture was stirred for 30 min at this temperature, the cold bath removed, and the reaction mixture stirred for a further 30 min. Subsequently, the reaction mixture was again cooled to -78 °C and 1.1·*n* equivs. $ClP(OEt)_2$ added dropwise over the course of 5 min. The reaction mixture was then slowly warmed to RT with stirring over the course of 16 h. All volatiles were subsequently removed *in vacuo*, the product extracted with Et_2O (10 mL/g) and slowly added to a suspension of $LiAlH_4$ and Me_3SiCl (1:1, 2.5 equivs.) in Et_2O (10 mL/g) at -78°C. The mixture was slowly warmed to RT with stirring over the course of 16 h. Subsequently, the reaction was quenched with degassed H_2O (~10 equivs.). The organic and aqueous phases were separated, and the aqueous phase washed with Et_2O. The combined organic phases were dried over dry $MgSO_4$. Volatiles were removed *in vacuo* to give the respective phosphine as a yellow to orange oil or oily solid, which was used without further purification.

General synthetic procedure 2 (for NHC-phosphanylidene synthesis)

^{iPr}NHC was added to a solution of the respective bis-phosphine $Ar(PH_2)_2$ in toluene and the reaction mixture heated to 100 °C for 16 h. Subsequently, all volatiles were removed *in vacuo*. Pentane was added and the mixture sonicated in an ultrasonic bath for 30 min. After filtration, the precipitate was washed with pentane and dried at 80 °C for 1 h to yield the respective bis-phosphanylidene as a brightly coloured powder. Crystallization was different for each product and is described in more detail for each compound (see below).

Synthesis of 1-(^{iPr}NHC·P)-benzene, 1.

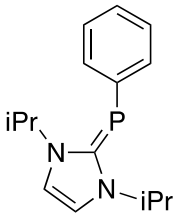

1 was synthesized according to a known literature procedure.[5]

NMR data was in agreement with previously reported data.[5] UV/vis data was taken as additional information.

λ_{max} (tol), nm (ε, $Lmol^{-1}$ cm^{-1}): 346 (4000), 420 (4900).

λ_{max} (THF), nm (ε, $Lmol^{-1}$ cm^{-1}): 346 (5400), 422 (6300).

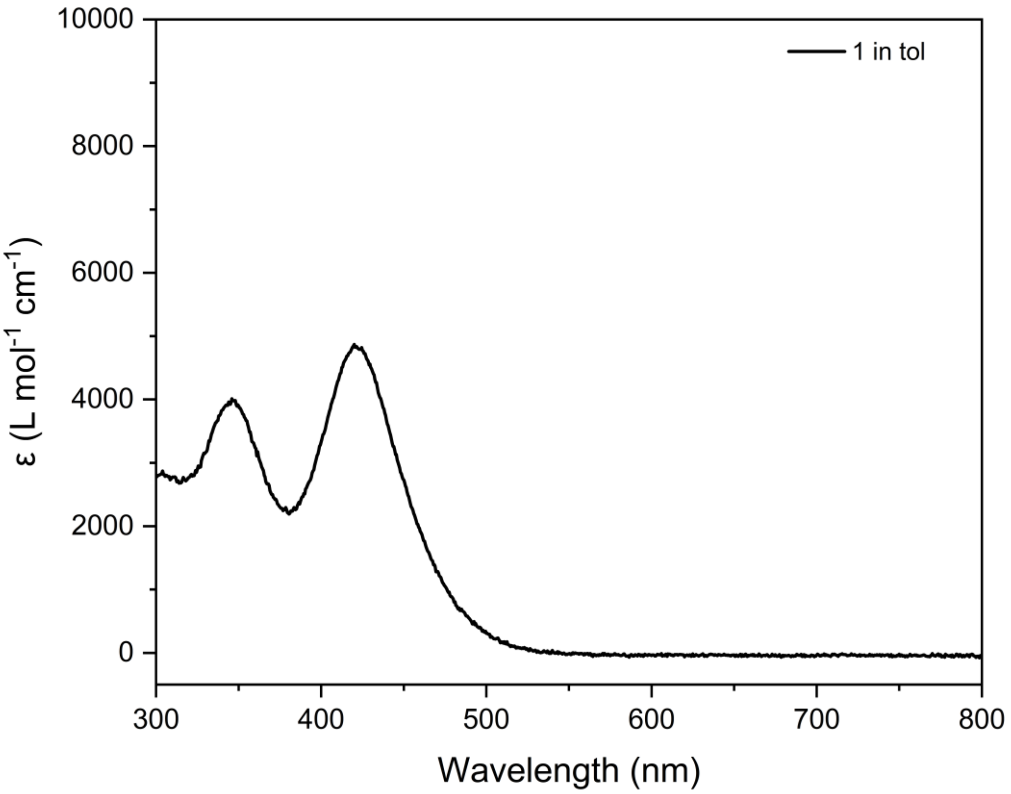

Figure S1. UV/vis spectrum of a 1x10^{-4} M solution of **1** in toluene at ambient temperature.

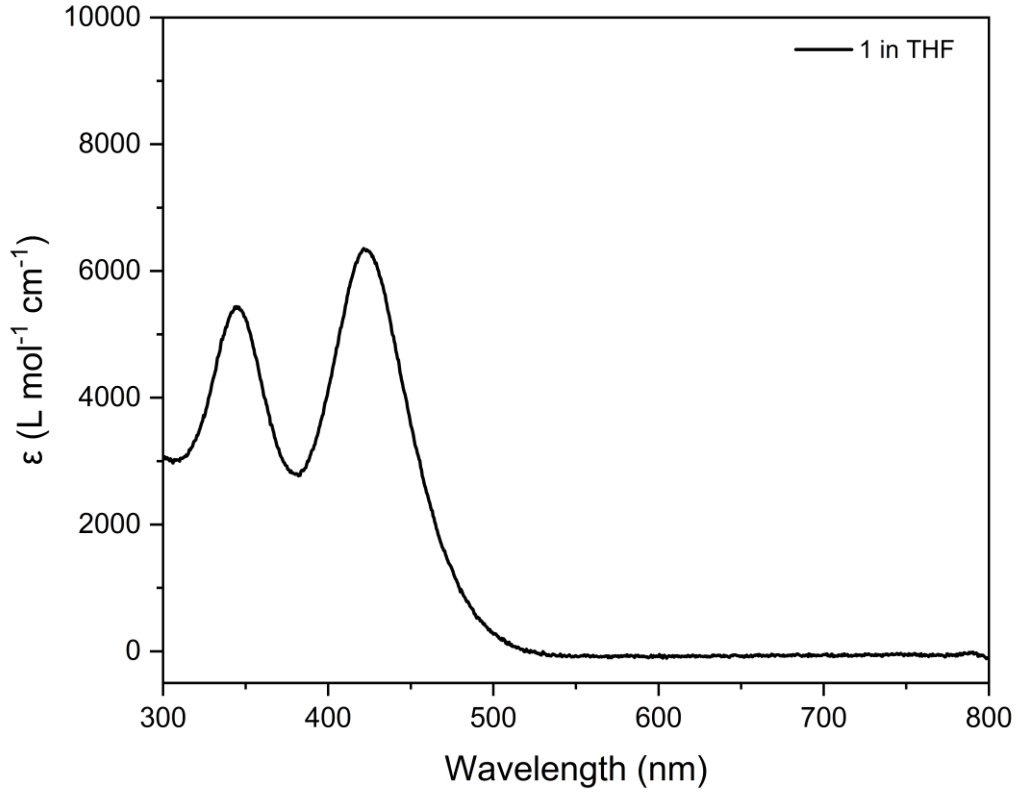

Figure S2. UV/vis spectrum of a 1x10^{-4} M solution of **1** in THF at ambient temperature.

Synthesis of 1,4-Me$_2$-2,5-(PH$_2$)$_2$-benzene.

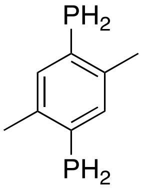

The compound was obtained using the general procedure 1, using 1,4-dibromo-2,5-(bis)methylbenzene (0.500 g, 1.89 mmol) in THF (35 mL), *t*-BuLi (1.7 M in hexanes, 5.0 mL), ClP(OEt)$_2$ (0.57 mL, 3.98 mmol), LiAlH$_4$ (179 mg, 4.74 mmol) and Me$_3$SiCl (0.60 mL, 4.74 mmol). The compound was used without further purification.

Spectroscopic data was in agreement with previously reported data.[6]

Synthesis of 1,4-Me$_2$-2,5-(^{iPr}NHC·P)$_2$-benzene, 2a.

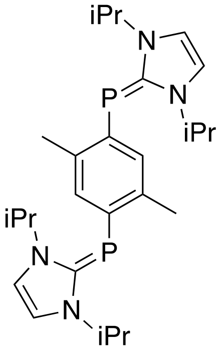

The compound was obtained using the general procedure 2, using *p*-MePH$_2$ (0.322 g, 1.89 mmol - assuming 100% yield in the previous step), ^{iPr}NHC (1.15 mL, 7.57 mmol). Compound **2a** was isolated as an orange solid (286 mg, 32 %).

^{1}H NMR (400 MHz, C$_6$D$_6$, 298 K): δ = 0.94 (d, $^3J_{HH}$ = 6.7 Hz, 24H, NHC-^{iPr}CH_3), 2.69 (s, 6H, Ph-CH_3), 5.03 (pd, J_{HH} = 6.6, 3.8 Hz, 4H, NHC-^{iPr}C*H*), 6.19 (s, 4H, NHC-C*H*), 7.52 (t, $^4J_{HH}$ = 4.2 Hz, 2H, Ar*H*).

^{13}C{^{1}H} NMR (101 MHz, C$_6$D$_6$, 298 K): δ = 21.7 (NHC-^{iPr}*C*H$_3$), 22.7 (d, $^3J_{CP}$ = 18.0 Hz Ph-*C*H$_3$), 49.3 (d, $^3J_{CP}$ = 10.8 Hz, NHC-^{iPr}*C*H), 114.7 (d, $^3J_{CP}$ = 3.1 Hz, NHC-*C*H), 135.0 (m, Ar*C*H), 136.5 (d, J_{CP} = 18.6, 2.2 Hz, Ar*C*), 140.5 (d, $^1J_{CP}$ = 46.4 Hz, Ar*C*), 168.8 (d, $^1J_{CP}$ = 104.5 Hz, NHC-*C*).

^{31}P{^{1}H} NMR (162 MHz, C$_6$D$_6$, 298 K): δ = - 68.9 (s, ^{iPr}NHC-*P*).

Anal.calcd. for C$_{26}$H$_{40}$N$_4$P$_2$: C, 66.36%; H, 8.57%; N, 11.91%; found: C, 64.73%; H, 8.00%; N, 11.44%.

N.B. After several runs, consistently low C values were found for this compound, which we believe to be due to incomplete combustion due to the presence of phosphorous.

MS/LIFDI-HRMS found (calcd.) m/z: 470.2713 (470.2725) for $[M]^+$.

λ_{max} (tol), nm (ε, $Lmol^{-1}$ cm^{-1}): 351 (9900), 453 (14100).

λ_{max} (THF), nm (ε, $Lmol^{-1}$ cm^{-1}): 346 (10300), 451 (14900).

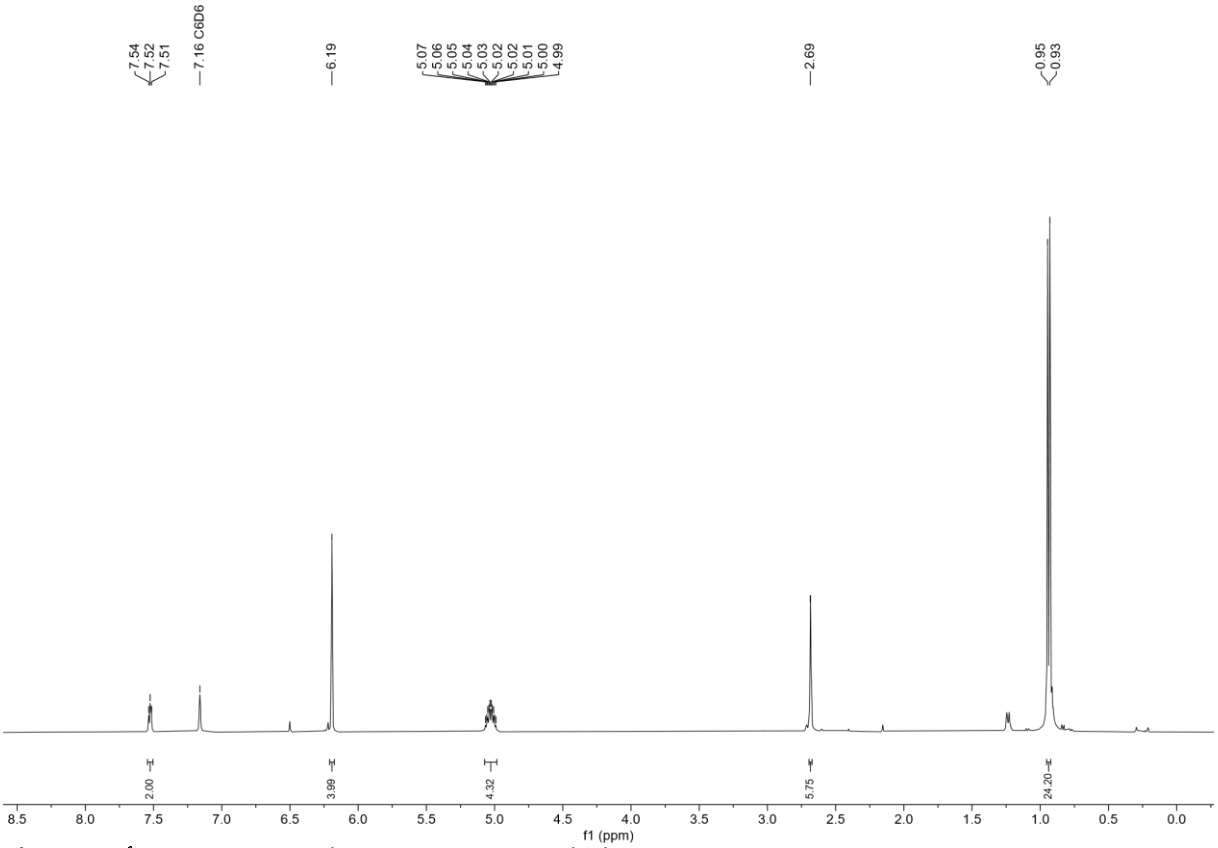

Figure S3. ^1H NMR spectrum (400 MHz, C_6D_6, 298 K) of **2a**.

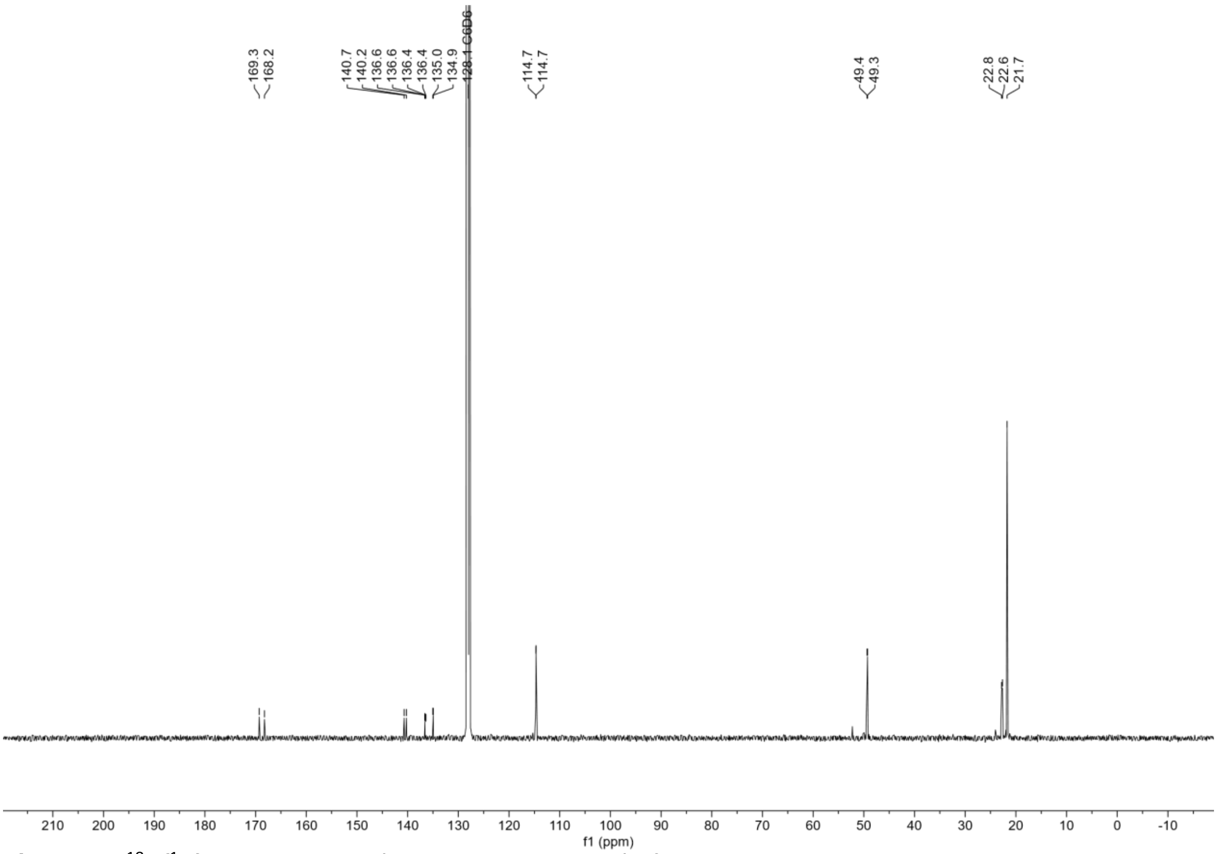

Figure S4. $^{13}C\{^{1}H\}$ NMR spectrum (101 MHz, C_6D_6, 298 K) of **2a**.

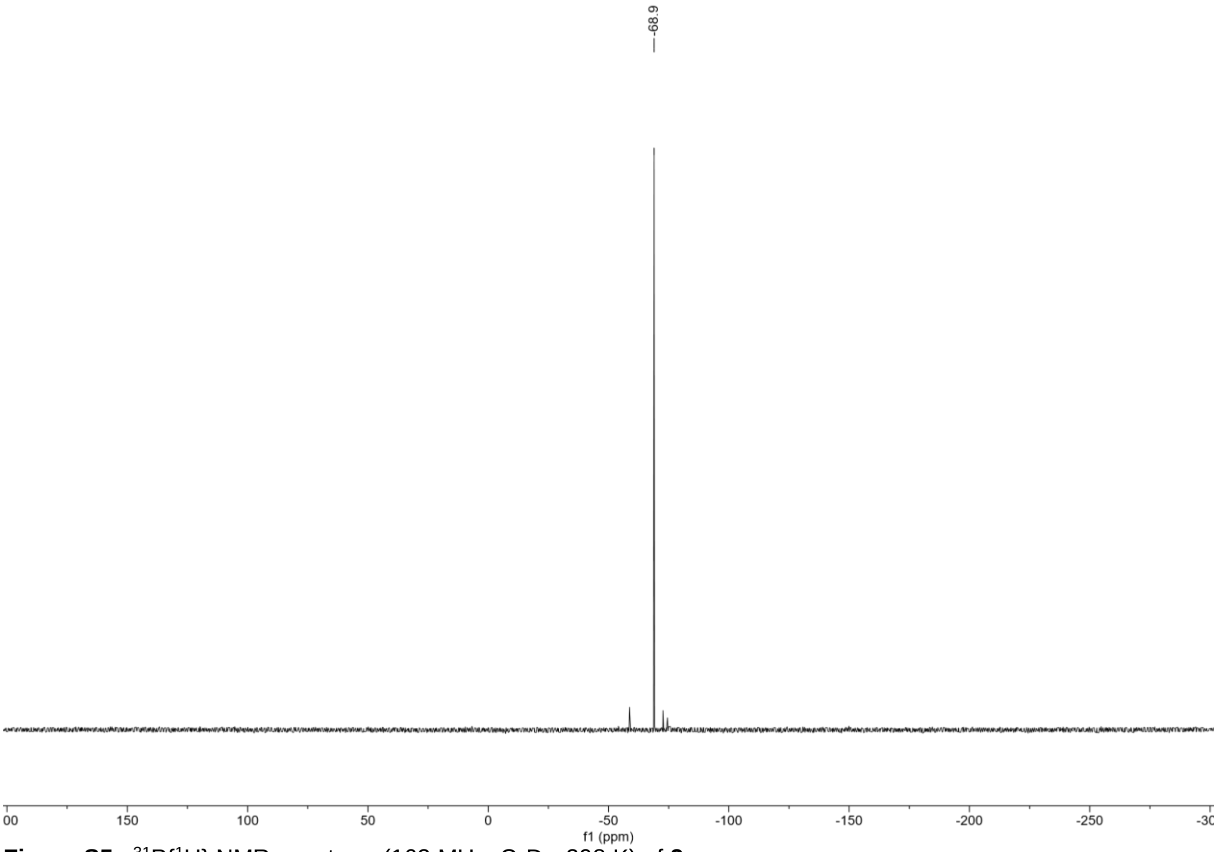

Figure S5. $^{31}P\{^{1}H\}$ NMR spectrum (162 MHz, C_6D_6, 298 K) of **2a**.

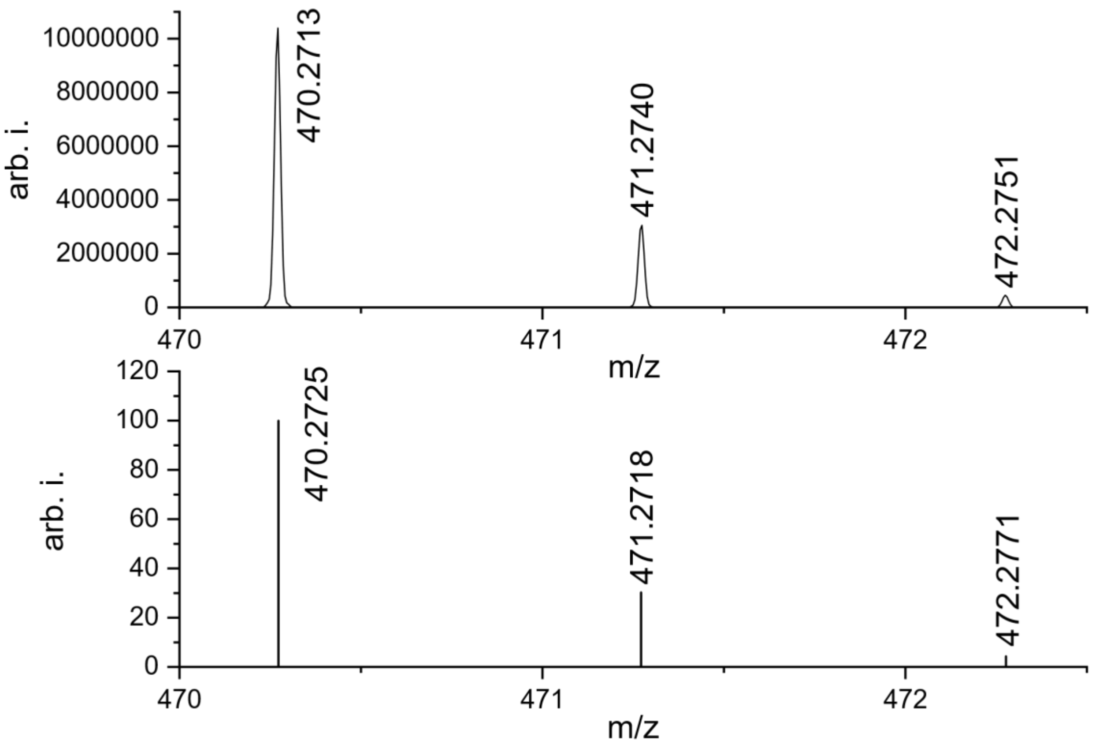

Figure S6. Cutout from LIFDI/MS of **2**, Top: found MS for [**2a**]$^+$; Bottom: Calculated MS spectrum of [**2a**]$^+$.

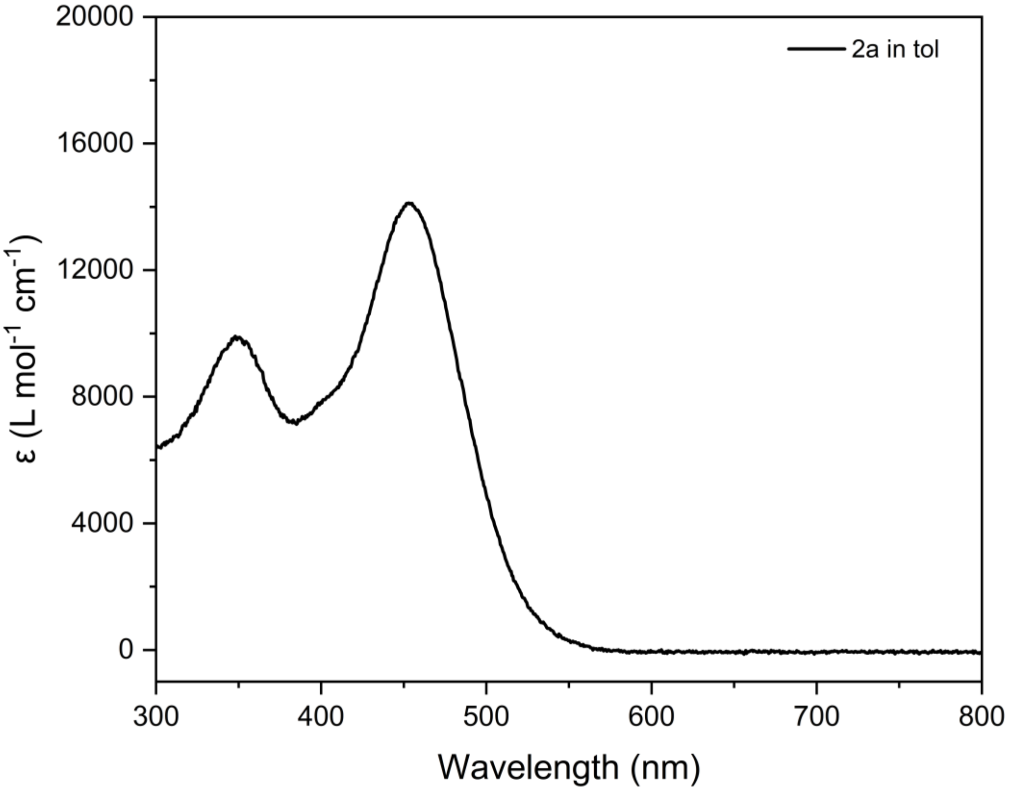

Figure S7. UV/vis spectrum of a 5x10^{-5} M solution of **2a** in toluene at ambient temperature.

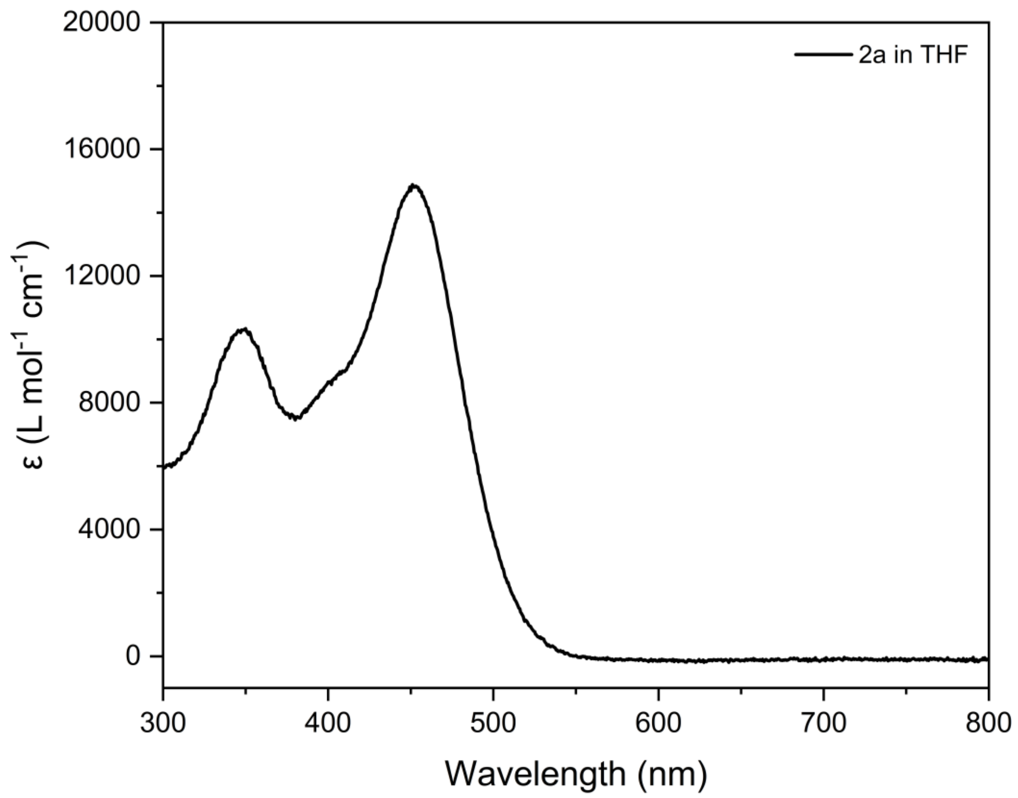

Figure S8. UV/vis spectrum of a $5x10^{-5}$ M solution of **2a** in THF at ambient temperature.

Synthesis of 1,4-$^{i}Pr_2$-2,5-(PH_2)$_2$-benzene.

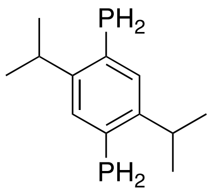

The compound was obtained using the general procedure 1, using 1,4-dibromo-2,5-(bis)isopropylbenzene (3.00 g, 9.37 mmol) in THF (100 mL), *t*-BuLi (1.7 M in hexanes, 24.8 mL), $ClP(OEt)_2$ (2.83 mL, 19.7 mmol), $LiAlH_4$ (888 mg, 23.4 mmol) and Me_3SiCl (2.97 mL, 23.4 mmol). The product (1.01 g, 48 %) was used without further purification.

$^{31}P\{^1H\}$ NMR (162 MHz, C_6D_6, 298 K): δ = - 129.9 (s, Ar*P*H_2).

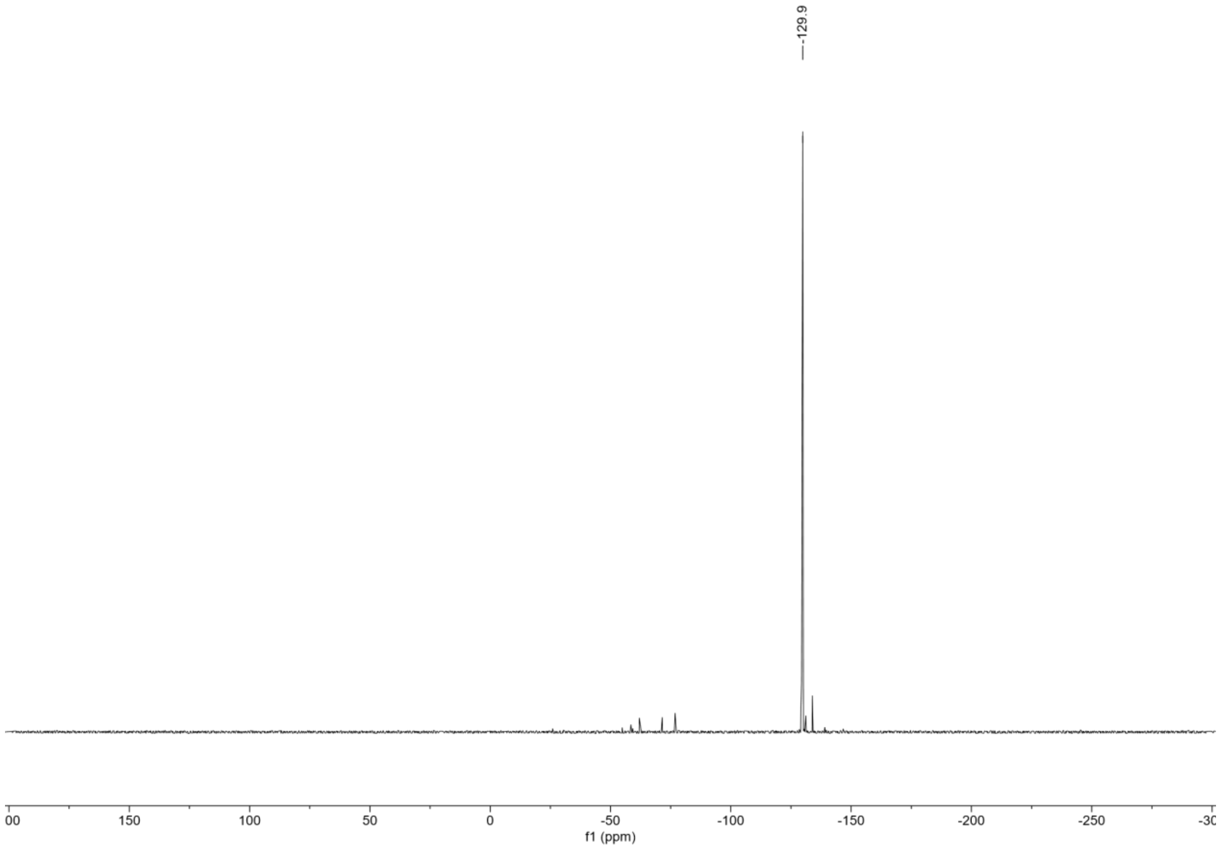

Figure S9. $^{31}P\{^1H\}$ NMR spectrum (162 MHz, C_6D_6, 298 K) of ***p*-iPrPhPH₂**.

Synthesis of 1,4-^{i}Pr$_2$-2,5-(^{iPr}NHC·P)$_2$-benzene, 2b.

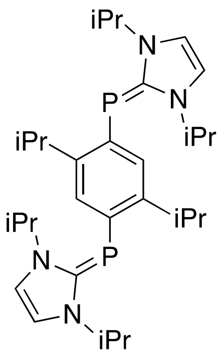

The compound was obtained using the general procedure 2, using *p*-iPrPhPH$_2$ (1.01 g, 4.46 mmol), ^{iPr}NHC (2.72 mL, 17.9 mmol). Compound **2b** was isolated as an orange solid (1.35 g, 57 %)

Orange crystals, suitable for SC-XRD analysis could be obtained from the pentane washing solution of **2b**.

^{1}H NMR (400 MHz, C_6D_6, 298 K): δ = 0.96 (d, $^{3}J_{HH}$ = 6.7 Hz, 24H, NHC-^{iPr}C*H*$_3$), 1.42 (d, $^{3}J_{HH}$ = 6.9 Hz, 12H, Ph-^{iPr}C*H*$_3$), 4.41 (h, $^{3}J_{HH}$ = 6.8 Hz, 2H, Ph-^{iPr}C*H*), 4.97 (pd, J_{HH} = 6.7, 3.8 Hz, 4H, NHC-^{iPr}C*H*), 6.21 (s, 4H, NHC-C*H*), 7.56 (t, $^{4}J_{HH}$ = 3.8 Hz, 2H, Ar*H*).

^{13}C{^{1}H} NMR (101 MHz, C_6D_6, 298 K): δ = 21.7 (NHC-^{iPr}*C*H$_3$), 24.3 (Ph-^{iPr}*C*H$_3$), 32.6 (d, $^{3}J_{CP}$ = 18.8 Hz, Ph-^{iPr}*C*H), 48.8 (d, $^{3}J_{CP}$ = 11.1 Hz, NHC-^{iPr}*C*H), 114.4 (d, $^{3}J_{CP}$ = 3.2 Hz, NHC-*C*H), 132.0 (Ar*C*), 140.1 (d, $^{1}J_{CP}$ = 45.9 Hz, Ar*C*), 147.6 (d, J_{CP} = 17.5 Hz, Ar*C*), 168.9 (d, $^{1}J_{CP}$ = 105.9 Hz, NHC-*C*).

^{31}P{^{1}H} NMR (162 MHz, C_6D_6, 298 K): δ = - 67.7 (s, ^{iPr}NHC-*P*).

Anal.calcd. for $C_{30}H_{48}N_4P_2$: C, 68.41%; H, 9.19%; N, 10.64%; found: C, 67.88%; H, 9.39%; N, 10.18%.

MS/LIFDI-HRMS found (calcd.) m/z: 526.3345 (526.3351) for $[M]^+$.

λ_{max} (tol), nm (ε, Lmol^{-1} cm^{-1}): 351 (12800), 447 (16900).

λ_{max} (THF), nm (ε, Lmol^{-1} cm^{-1}): 352 (11500), 448 (15900).

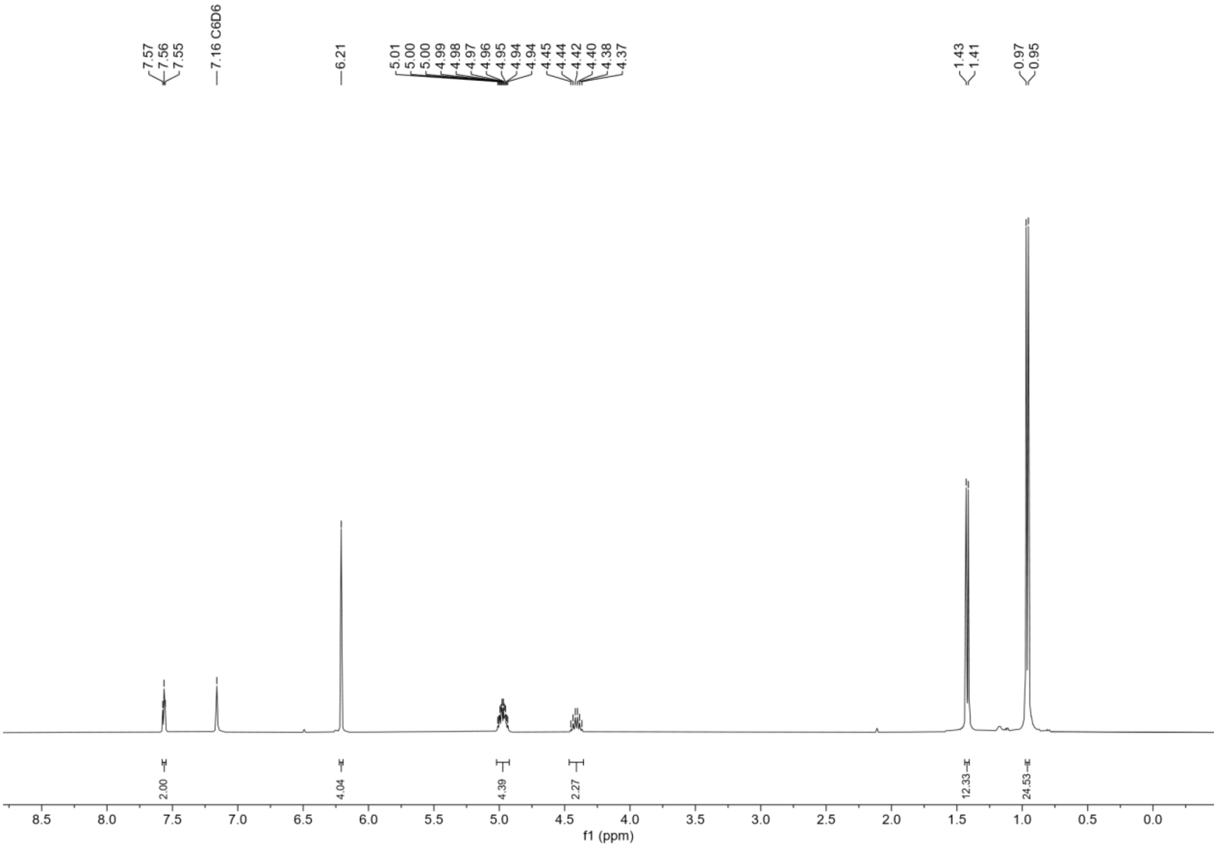

Figure S10. ^{1}H NMR spectrum (400 MHz, C_6D_6, 298 K) of **2b**.

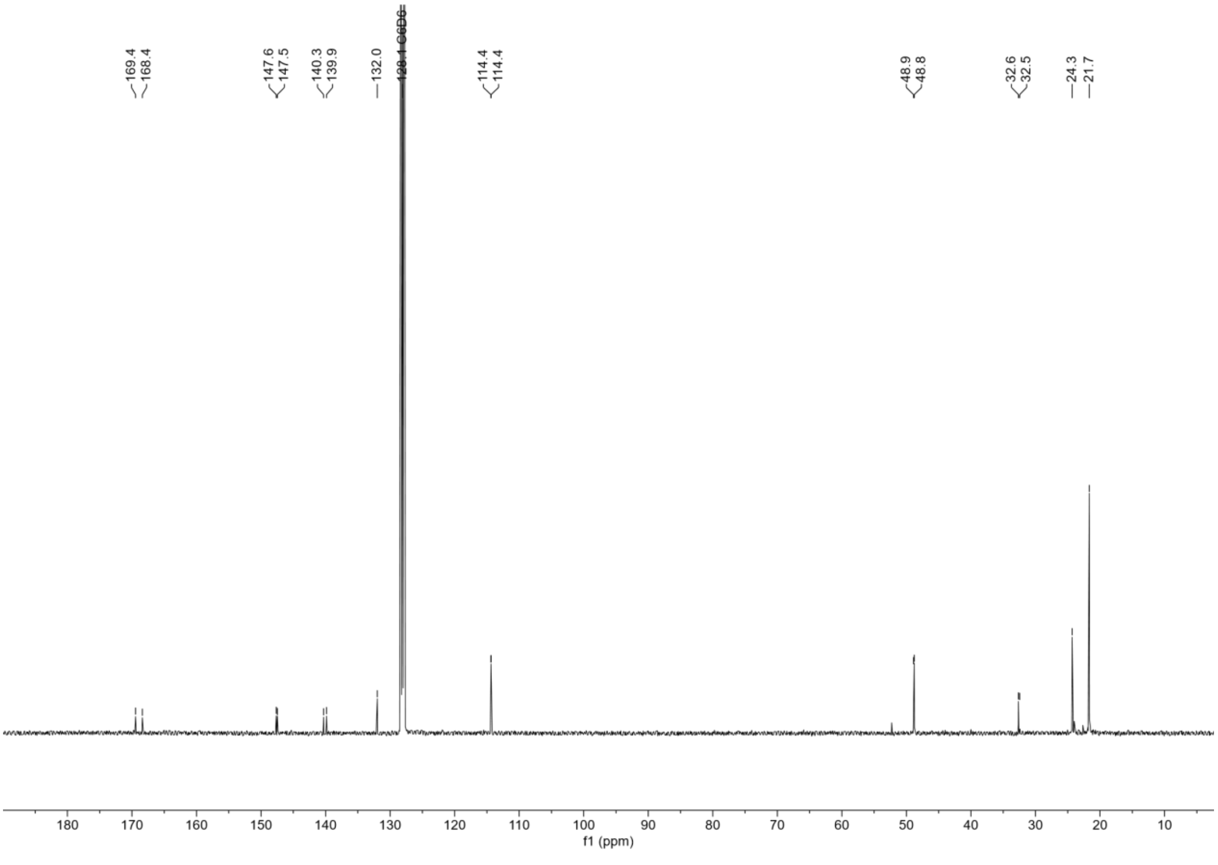

Figure S11. $^{13}C\{^{1}H\}$ NMR spectrum (101 MHz, C_6D_6, 298 K) of **2b**.

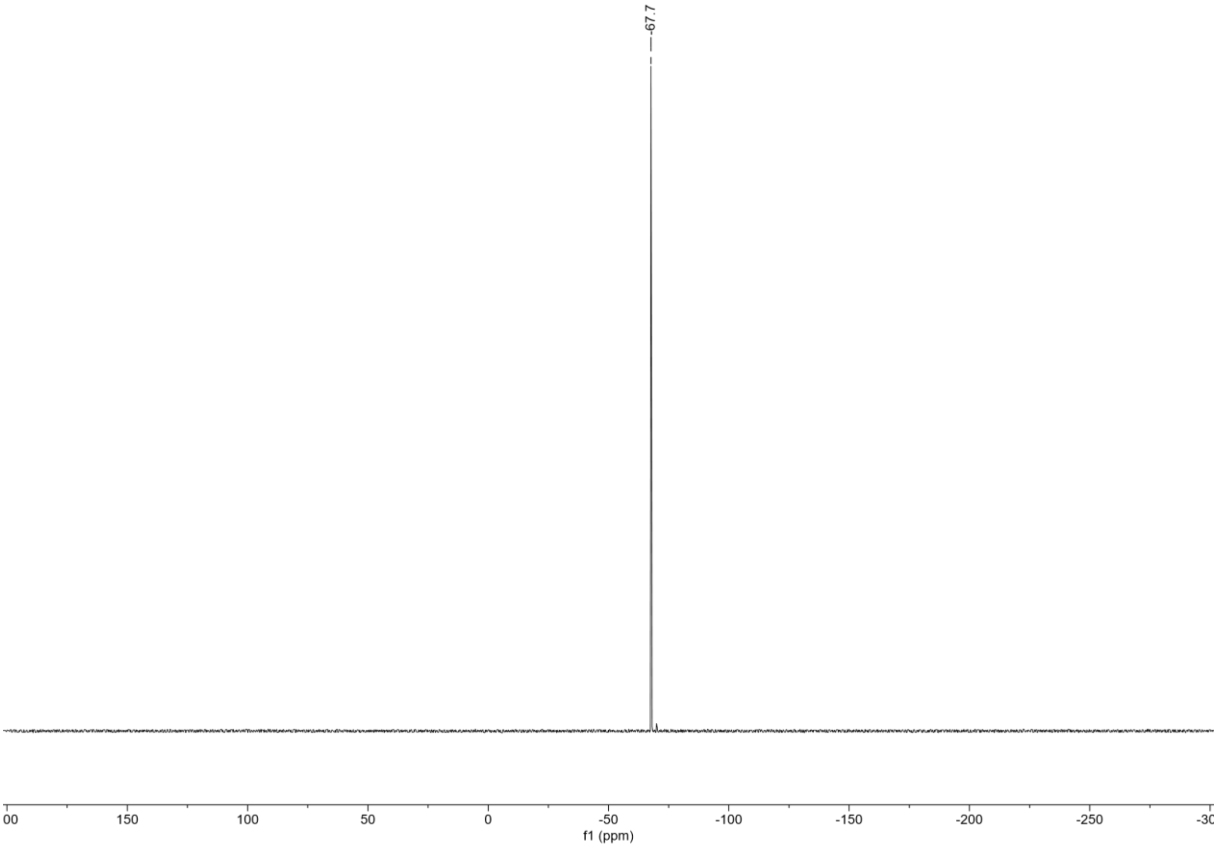

Figure S12. $^{31}P\{^{1}H\}$ NMR spectrum (162 MHz, C_6D_6, 298 K) of **2b**.

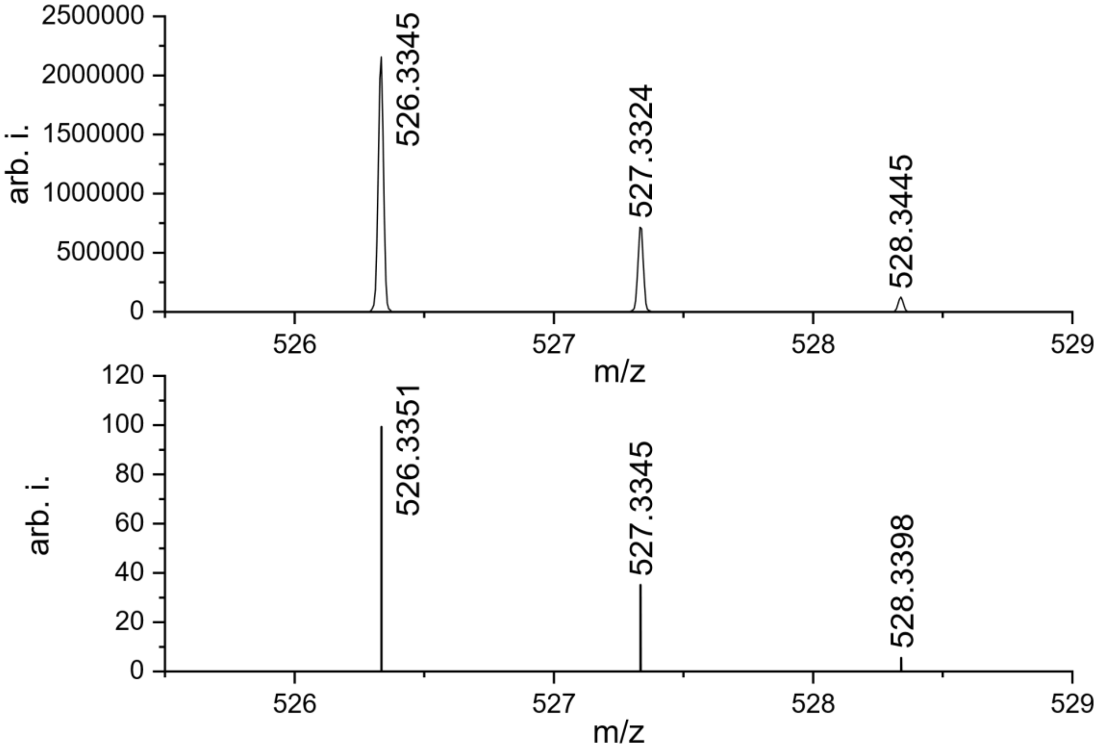

Figure S13. Cutout from LIFDI/MS of **4**, Top: found MS for [**2b**]$^+$; Bottom: Calculated MS spectrum of [**2b**]$^+$.

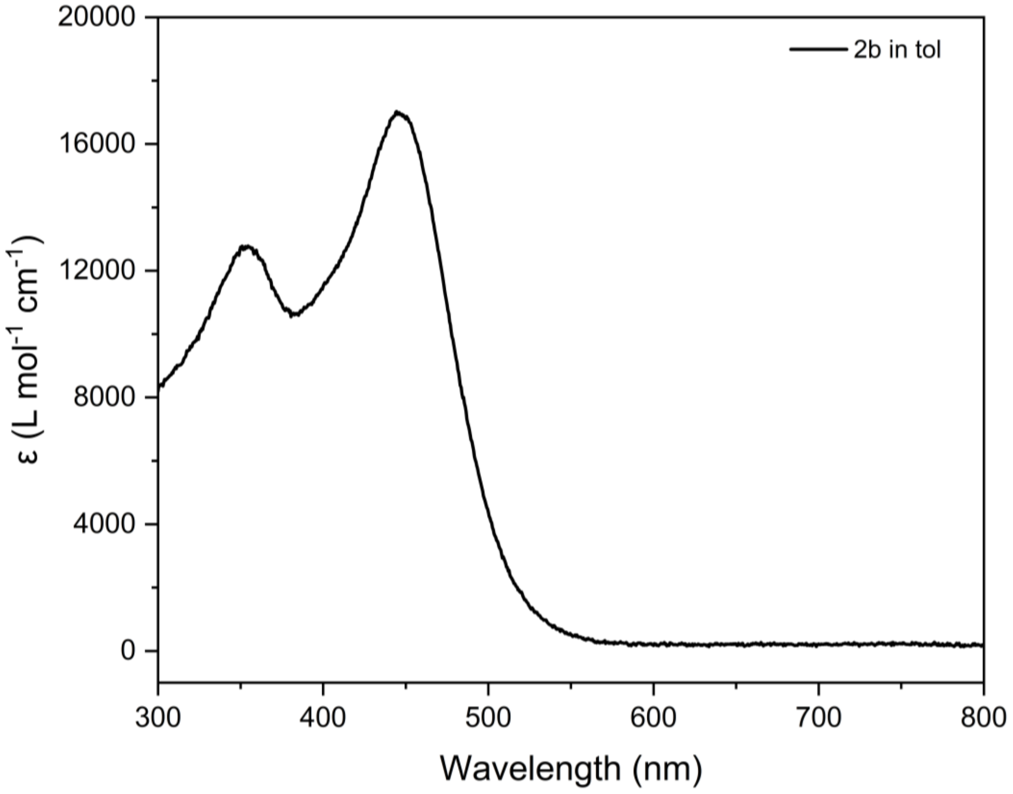

Figure S14. UV/vis spectrum of a 5x10^{-5} M solution of **2b** in toluene at ambient temperature.

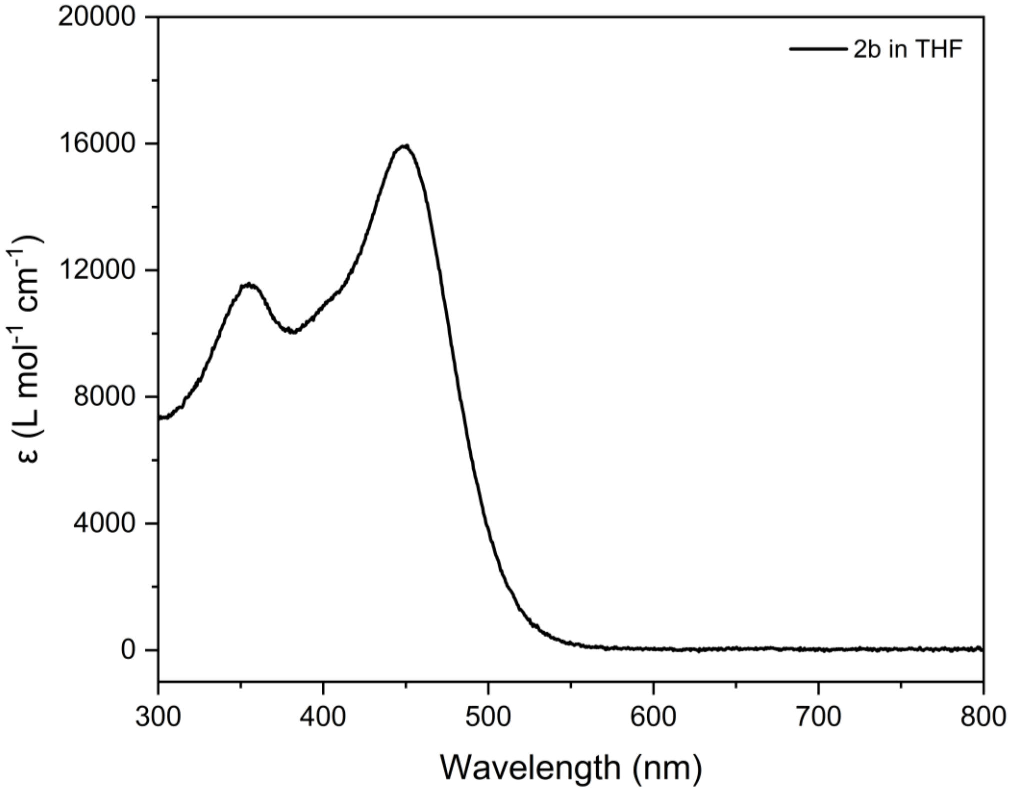

Figure S15. UV/vis spectrum of a 5x10^{-5} M solution of **2b** in THF at ambient temperature.

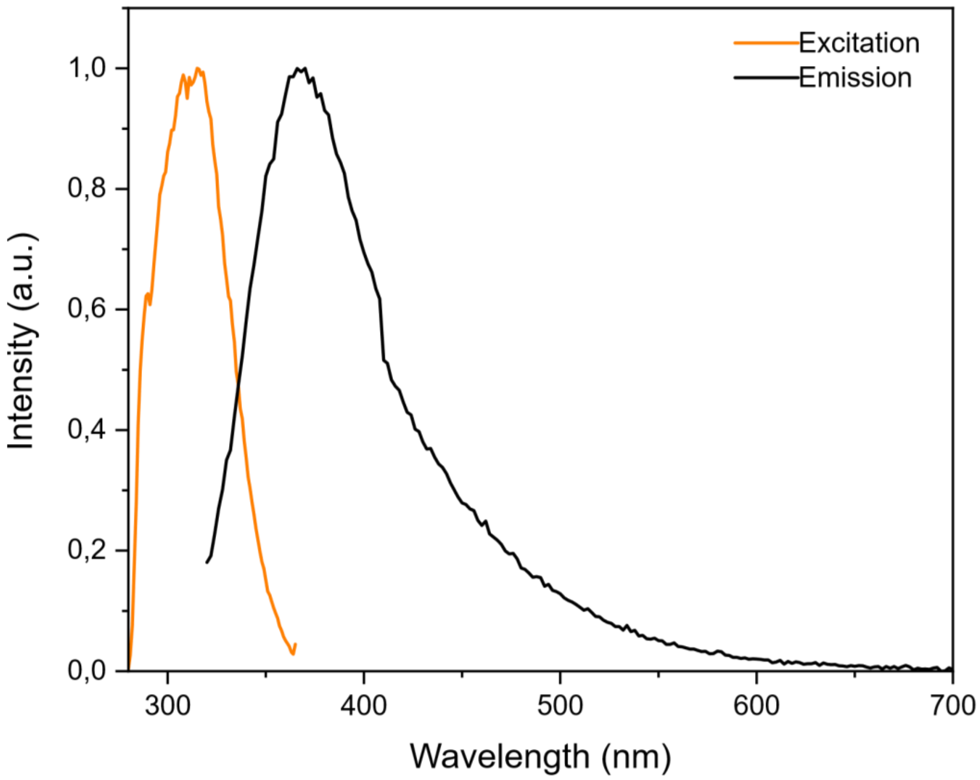

Figure S16. Orange: Excitation spectrum of **2b** in toluene; Black: Fluorescence emission spectrum of **2b** in toluene excited at 317 nm.

Synthesis of 1,5-$(PH_2)_2$-naphthylene.

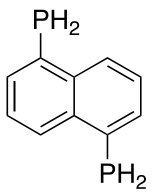

The compound was obtained using the general procedure 1, using 1,5-dibromonaphthalene (0.30 g, 1.05 mmol) in Et_2O (30 mL), *t*-BuLi (1.7 M in hexanes, 2.8 mL), $ClP(OEt)_2$ (0.32 mL, 2.20 mmol), $LiAlH_4$ (99 mg, 2.62 mmol) and Me_3SiCl (0.33 mL, 2.62 mmol) and was isolated as a yellow oil.

$^{31}P\{^1H\}$ NMR (162 MHz, C_6D_6, 298 K): δ = - 134.1 (s, Ar*P*H_2).

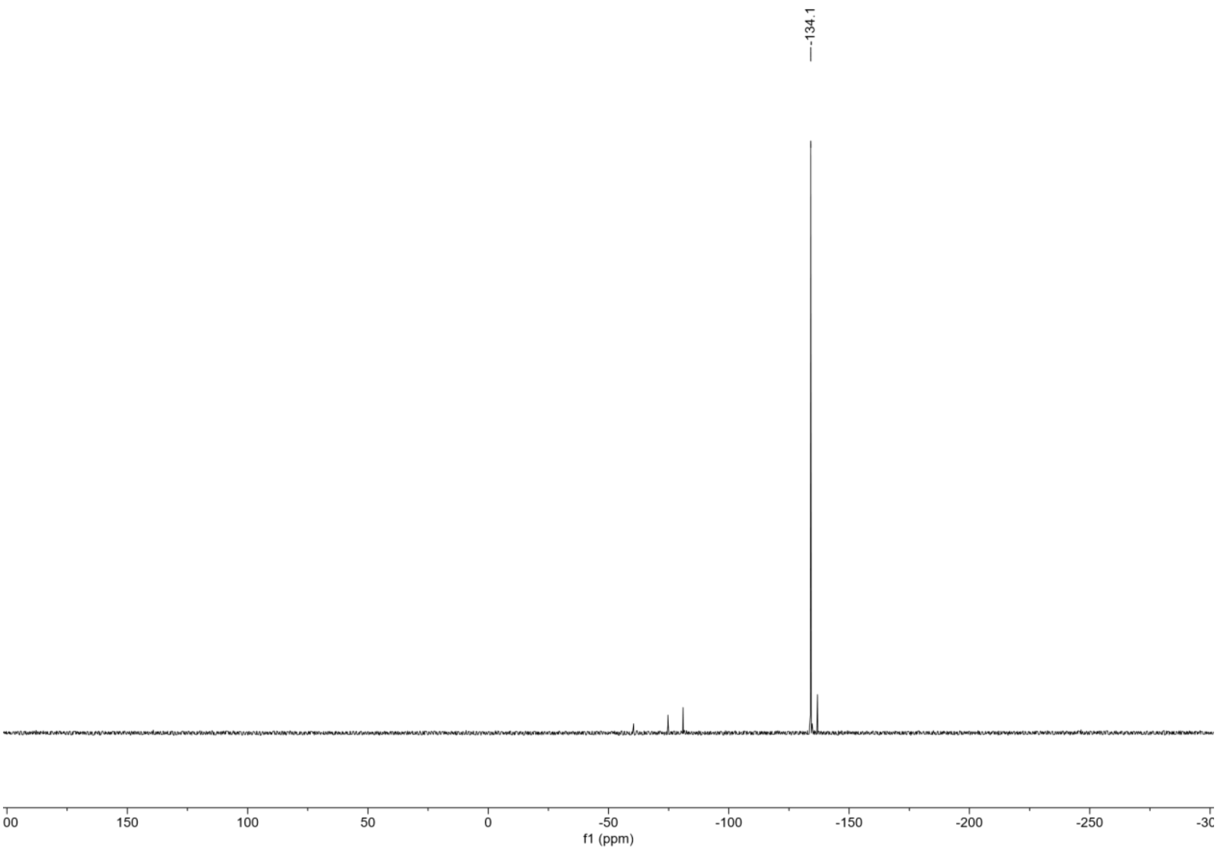

Figure S17. $^{31}P\{^1H\}$ NMR spectrum (162 MHz, C_6D_6, 298 K) of **1,5-*p*-NaphPH2.**

Synthesis of 1,5-(^{iPr}NHC·P)$_2$-naphthylene, 3.

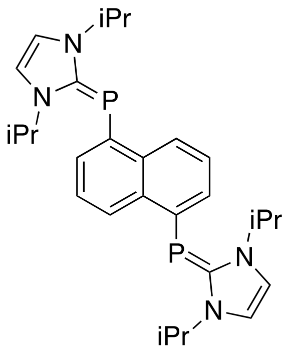

The compound was obtained using the general procedure 2, using 1,4-*p*-NaphPH$_2$ (285 mg, 1.48 mmol), ^{iPr}NHC (0.91 mL, 5.92 mmol). Compound **3** was isolated as an orange solid (205 mg, 28 %).

^{1}H NMR (400 MHz, C_6D_6, 298 K): δ = 0.85 (d, $^3J_{HH}$ = 6.7 Hz, 24H, NHC-^{iPr}CH_3), 5.00 (pd, J_{HH} = 6.7, 3.9 Hz, 4H, NHC-^{iPr}C*H*), 6.18 (s, 4H, NHC-C*H*), 7.23 (dd, J_{HH} = 8.3, 7.0 Hz, 2H, Ar*H*), 7.68 (ddd, J_{HH} = 7.1, 4.4, 1.2 Hz, 2H, Ar*H*), 9.00 (dd, J_{HH} = 8.3, 4.3 Hz, 2H, Ar*H*).

^{13}C{^{1}H} NMR (101 MHz, C_6D_6, 298 K): δ = 21.8 (NHC-*C*H$_3$), 49.7 (d, $^3J_{CP}$ = 10.1 Hz, NHC-^{iPr}*C*H), 115.1 (d, $^3J_{CP}$ = 3.2 Hz, NHC-*C*H), 124.3 (Ar*C*), 124.3 (d, J_{CP} = 3.1 Hz, Ar*C*), 124.6 (Ar*C*), 130.4 (d, J_{CP} = 7.9 Hz, Ar*C*), 136.9 (d, J_{CP} = 15.2 Hz, Ar*C*), 148.0 (d, $^1J_{CP}$ = 52.4 Hz, Ar*C*), 168.5 (d, $^1J_{CP}$ = 104.0 Hz, NHC-*C*).

^{31}P{^{1}H} NMR (162 MHz, C_6D_6, 298 K): δ = - 70.3 (s, ^{iPr}NHC-*P*).

Anal.calcd. for $C_{30}H_{48}N_4P_2$: C, 68.27%; H, 7.78%; N, 11.37%; found: C, 68.45%; H, 7.64%; N, 11.19%.

MS/LIFDI-HRMS found (calcd.) m/z: 492.2542 (492.2569) for $[M]^+$.

λ_{max} (tol), nm (ε, Lmol^{-1} cm^{-1}): 368 (8000), 502 (12900).

λ_{max} (THF), nm (ε, Lmol^{-1} cm^{-1}): 367 (6100), 504 (13400).

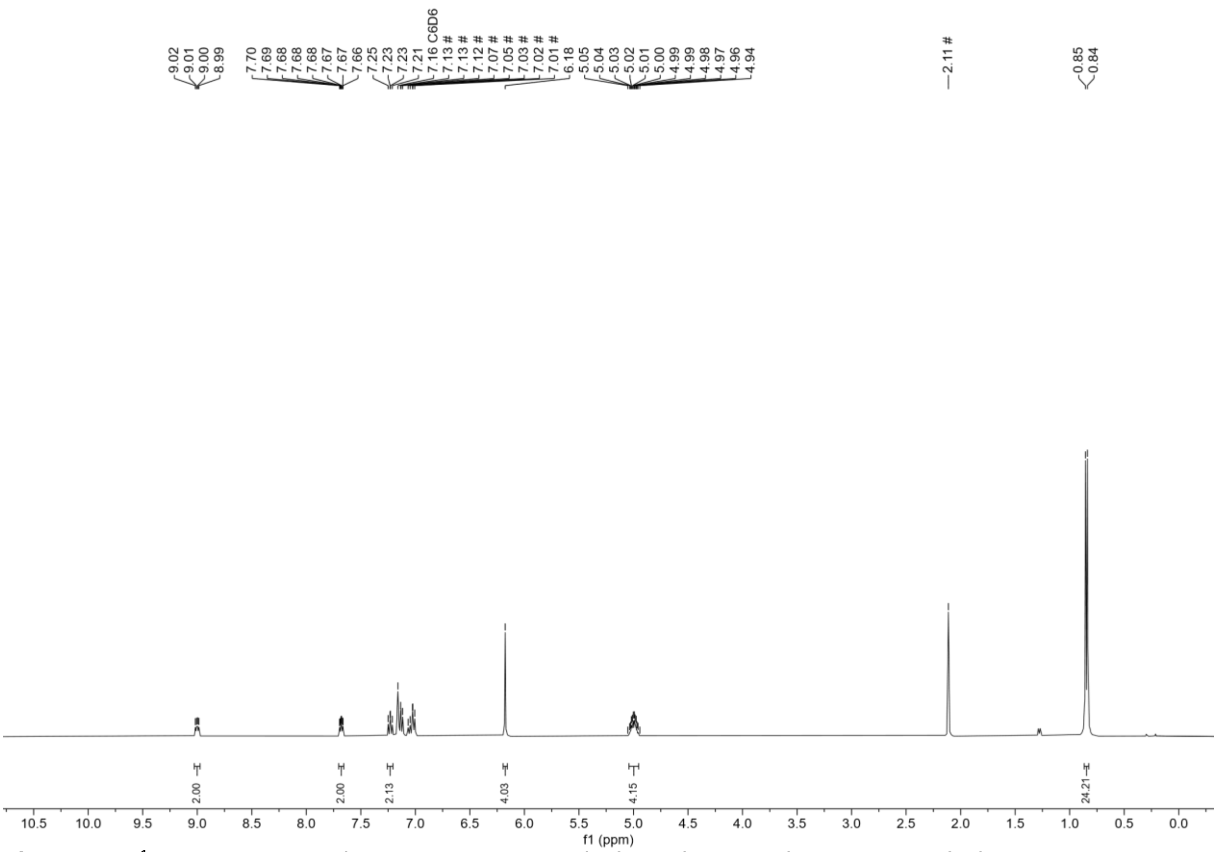

Figure S18. ^{1}H NMR spectrum (400 MHz, C_6D_6, 298 K) of **3**. # denotes minor amounts of toluene.

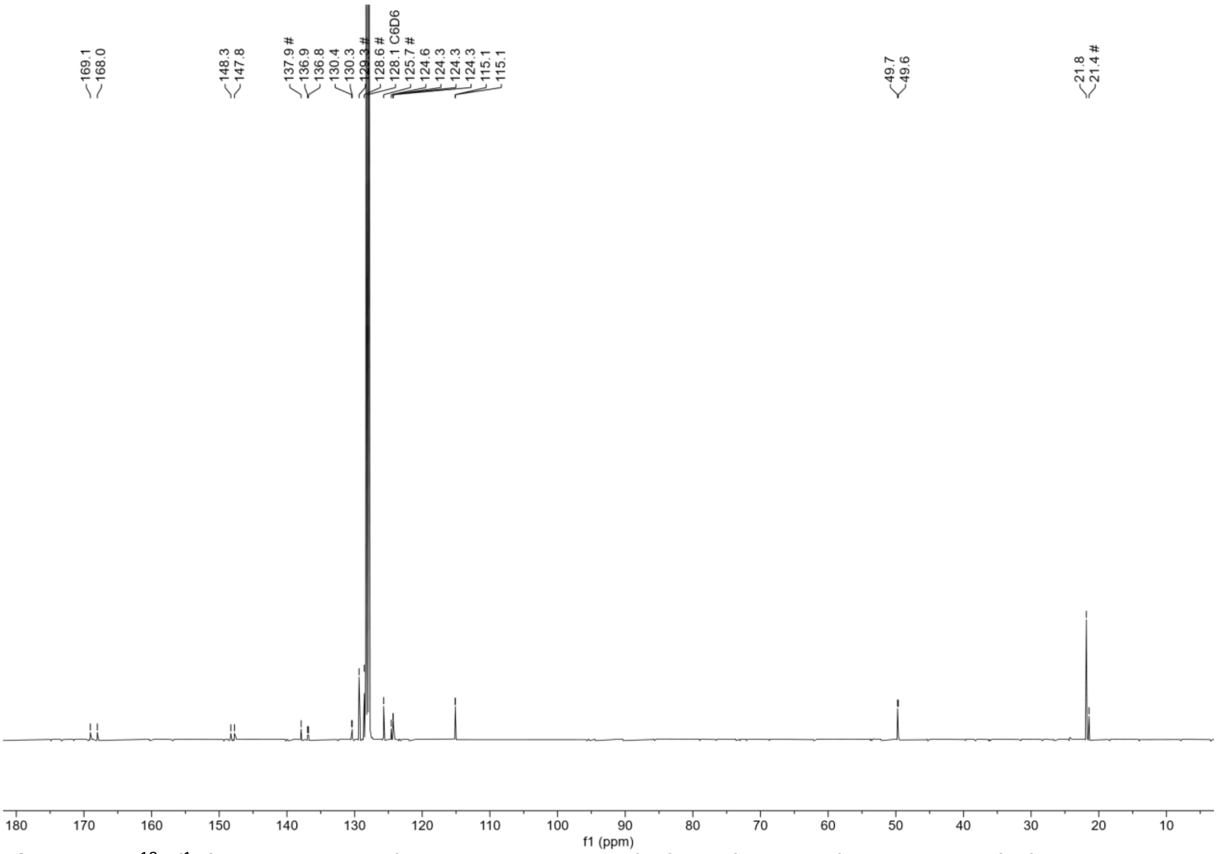

Figure S19. $^{13}C\{^{1}H\}$ NMR spectrum (101 MHz, C_6D_6, 298 K) of **3**. # denotes minor amounts of toluene.

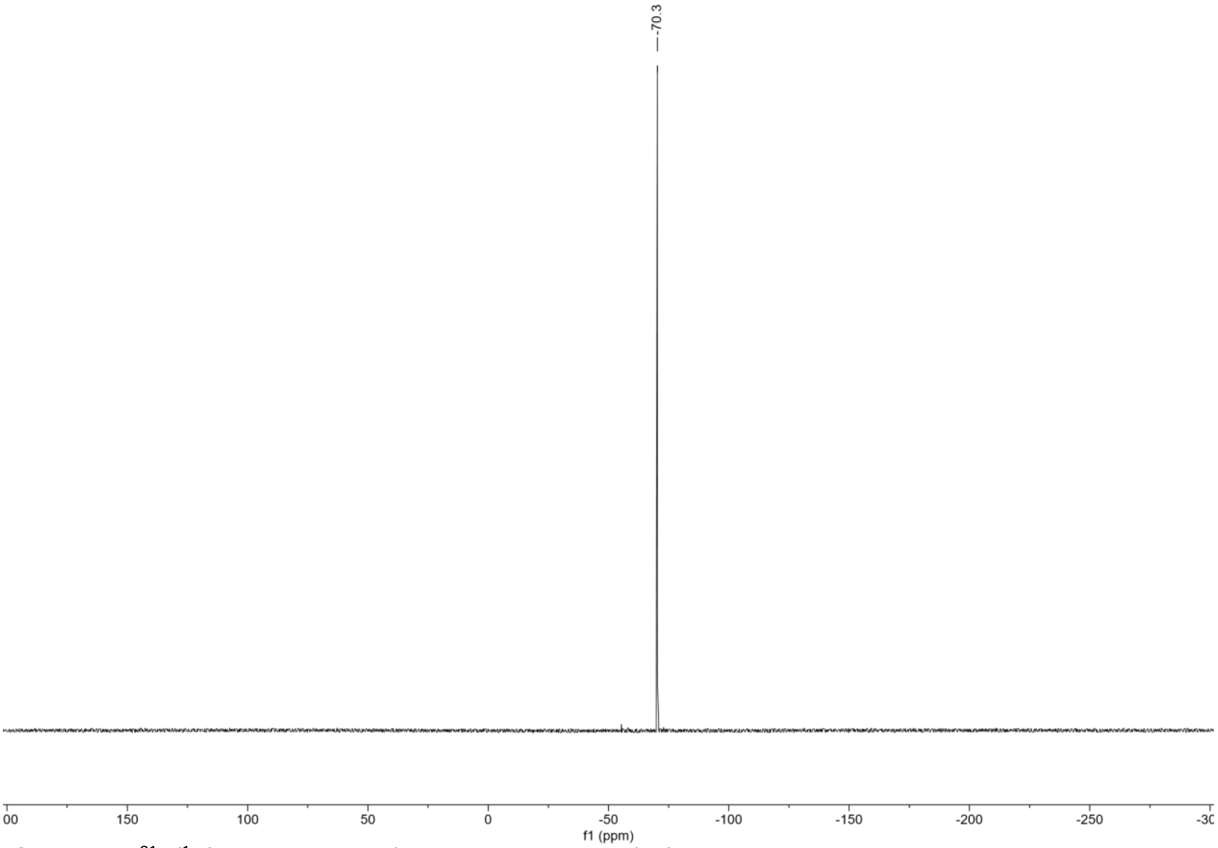

Figure S20. $^{31}P\{^{1}H\}$ NMR spectrum (162 MHz, C_6D_6, 298 K) of **3**.

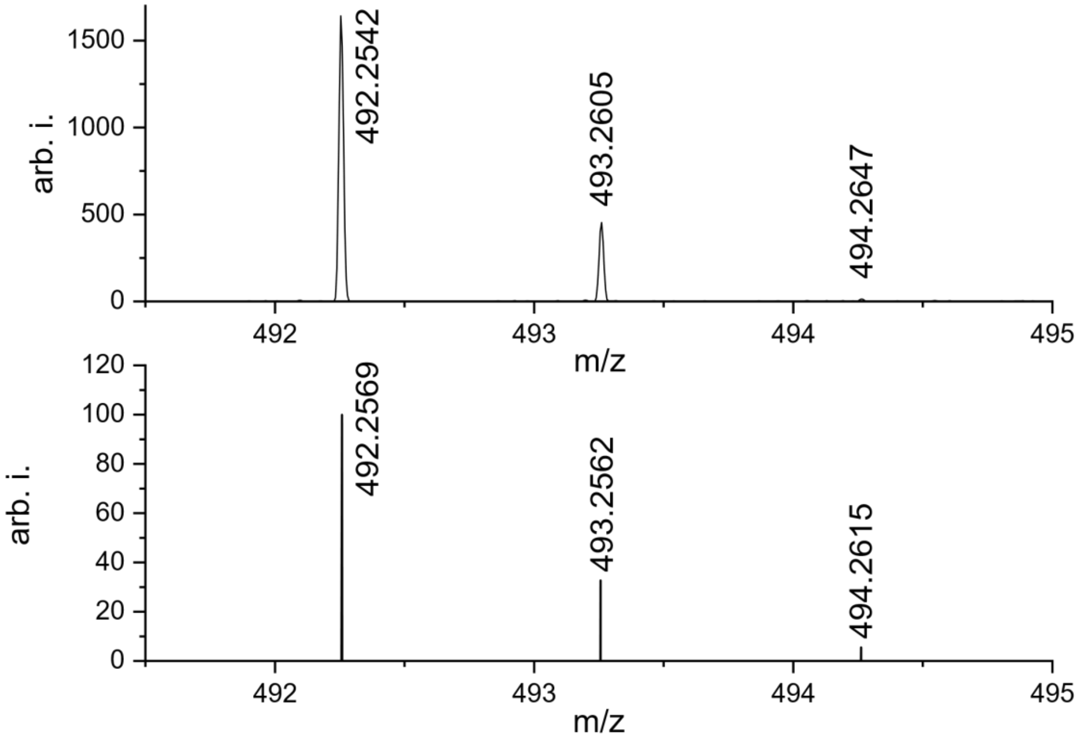

Figure S21. Cutout from LIFDI/MS of **3**, Top: found MS for [**3**]$^+$; Bottom: Calculated MS spectrum of [**3**]$^+$.

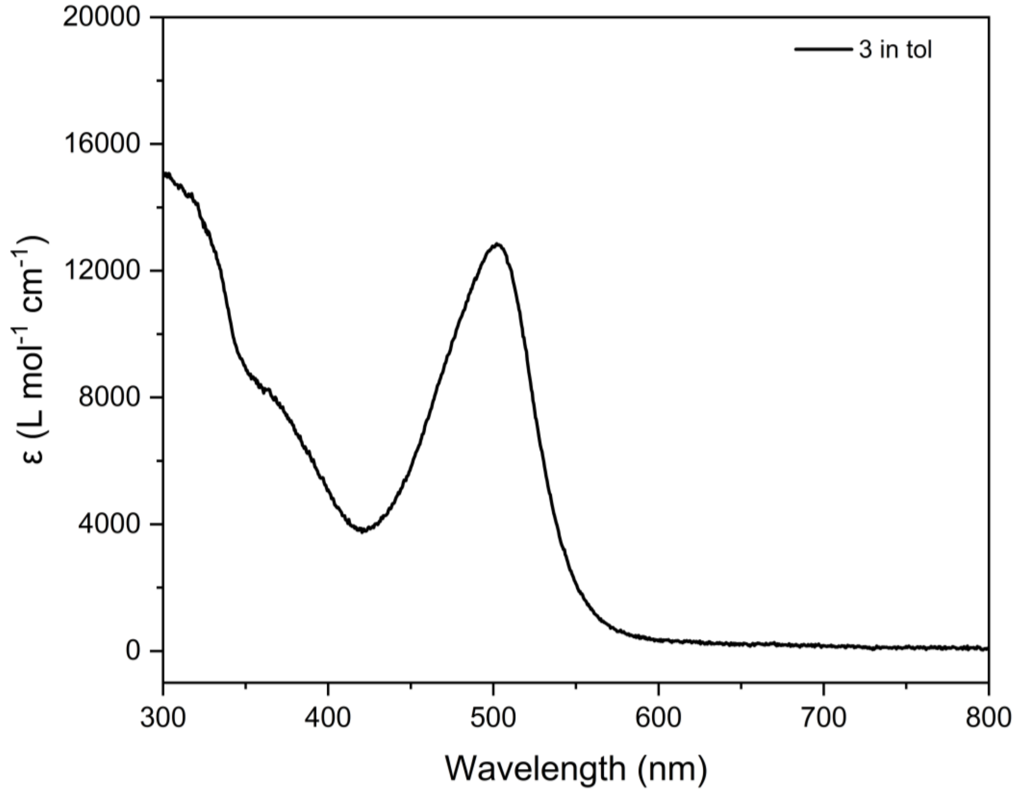

Figure S22. UV/vis spectrum of a 5x10^{-5} M solution of **3** in toluene at ambient temperature.

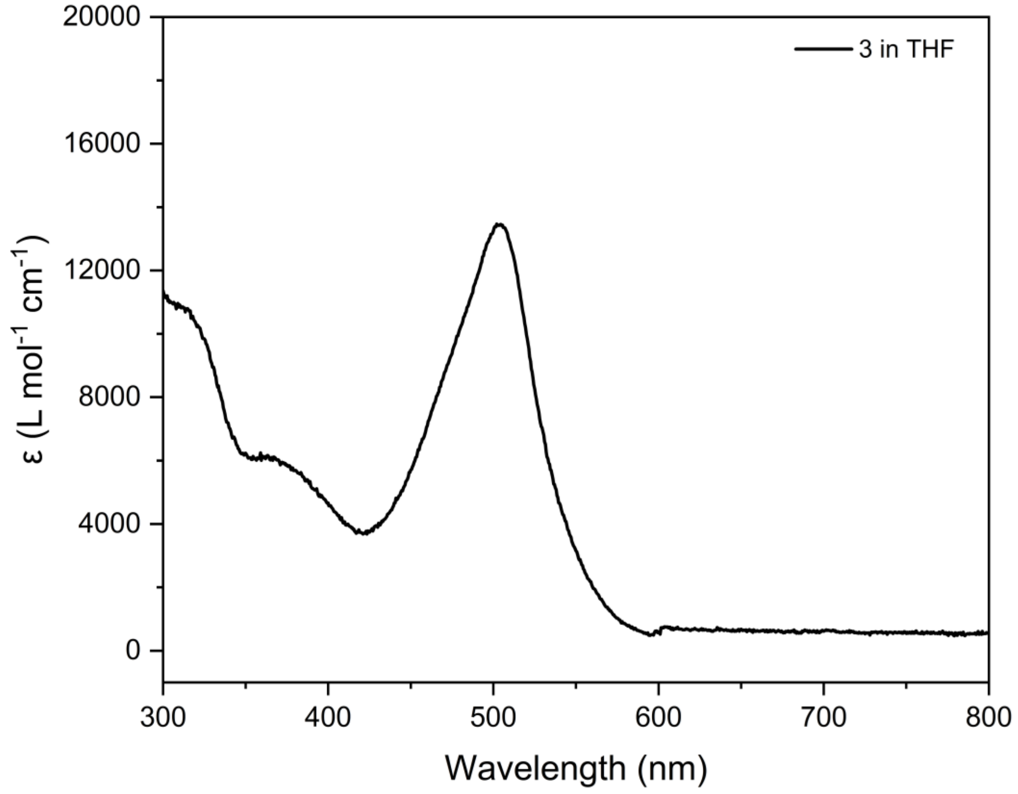

Figure S23. UV/vis spectrum of a 5x10^{-5} M solution of **3** in THF at ambient temperature.

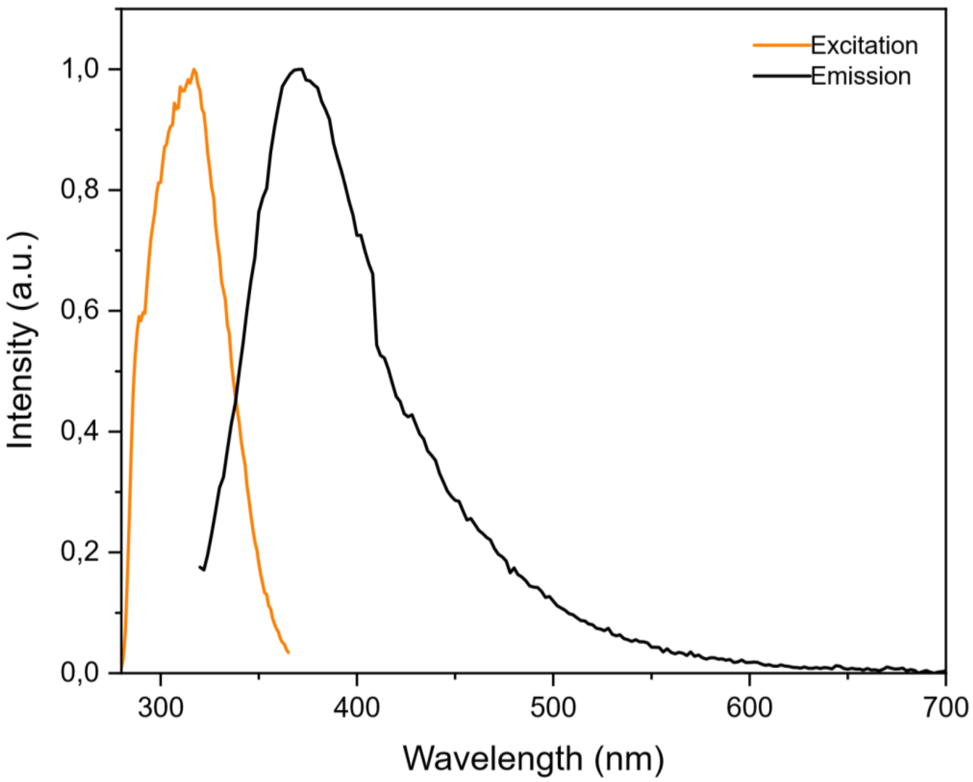

Figure S24. Orange: Excitation spectrum of **3** in toluene; Black: Fluorescence emission spectrum of **3** in toluene excited at 317 nm.

Synthesis of 1,4-(PH_2)$_2$-naphthylene.

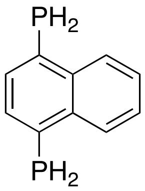

The compound was obtained using the general procedure 1, using 1,4-dibromonaphthalene (0.50 g, 1.75 mmol) in Et_2O (40 mL), *t*-BuLi (1.7 M in hexanes, 4.6 mL), $ClP(OEt)_2$ (0.54 mL, 3.67 mmol), $LiAlH_4$ (166 mg, 4.37 mmol) and Me_3SiCl (0.55 mL, 4.36 mmol) and was isolated as a yellow oil (285 mg, 85 %).

^{1}H NMR (400 MHz, C_6D_6, 298 K): δ = 3.95 (d, $^1J_{PH}$ = 202.3 Hz, 4H, P*H*$_2$), 7.25 (dd, *J* = 6.4, 3.3 Hz, 2H, Ar*H*), 7.29 (dd, *J* = 6.3, 3.3 Hz, 2H, Ar*H*), 7.94 - 8.15 (m, 2H, Ar*H*).

^{31}P{^{1}H} NMR (162 MHz, C_6D_6, 298 K): δ = - 132.7 (s, Ar*P*H$_2$).

^{31}P NMR (162 MHz, C_6D_6, 298 K): δ = - 132.7 (t, $^1J_{PH}$ = 201.2 Hz Ar*P*H$_2$).

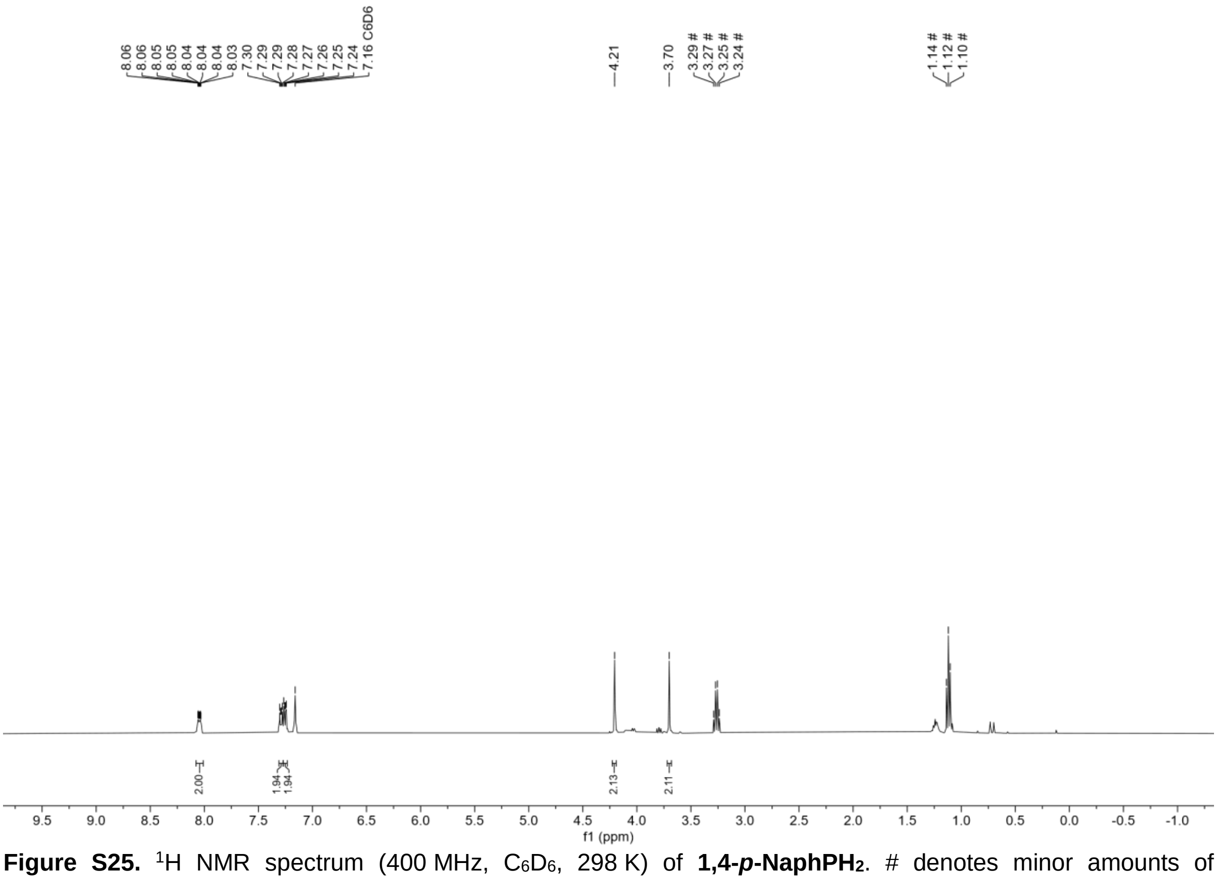

Figure S25. ^{1}H NMR spectrum (400 MHz, C_6D_6, 298 K) of **1,4-*p*-NaphPH$_2$**. # denotes minor amounts of diethylether.[7]

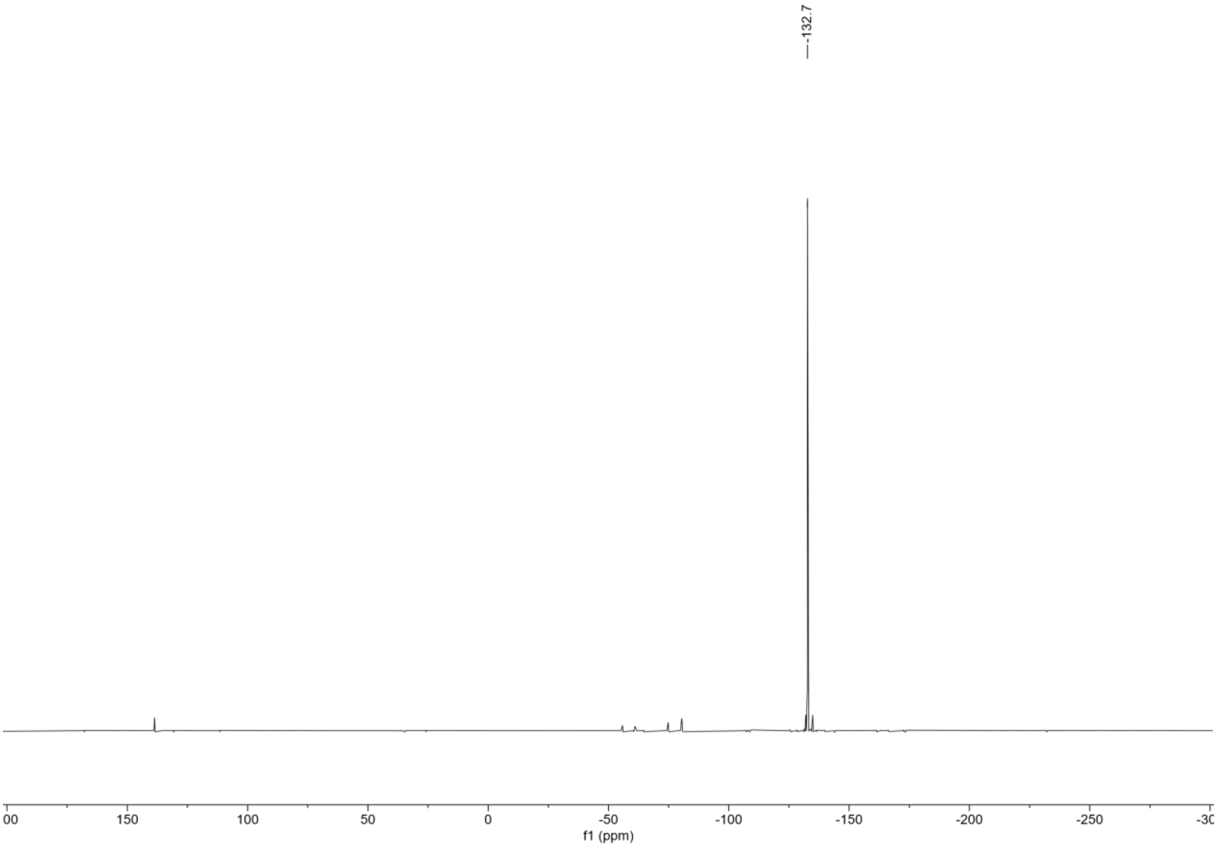

Figure S26. $^{31}P\{^{1}H\}$ NMR spectrum (162 MHz, C_6D_6, 298 K) of **1,4-*p*-NaphPH₂.**

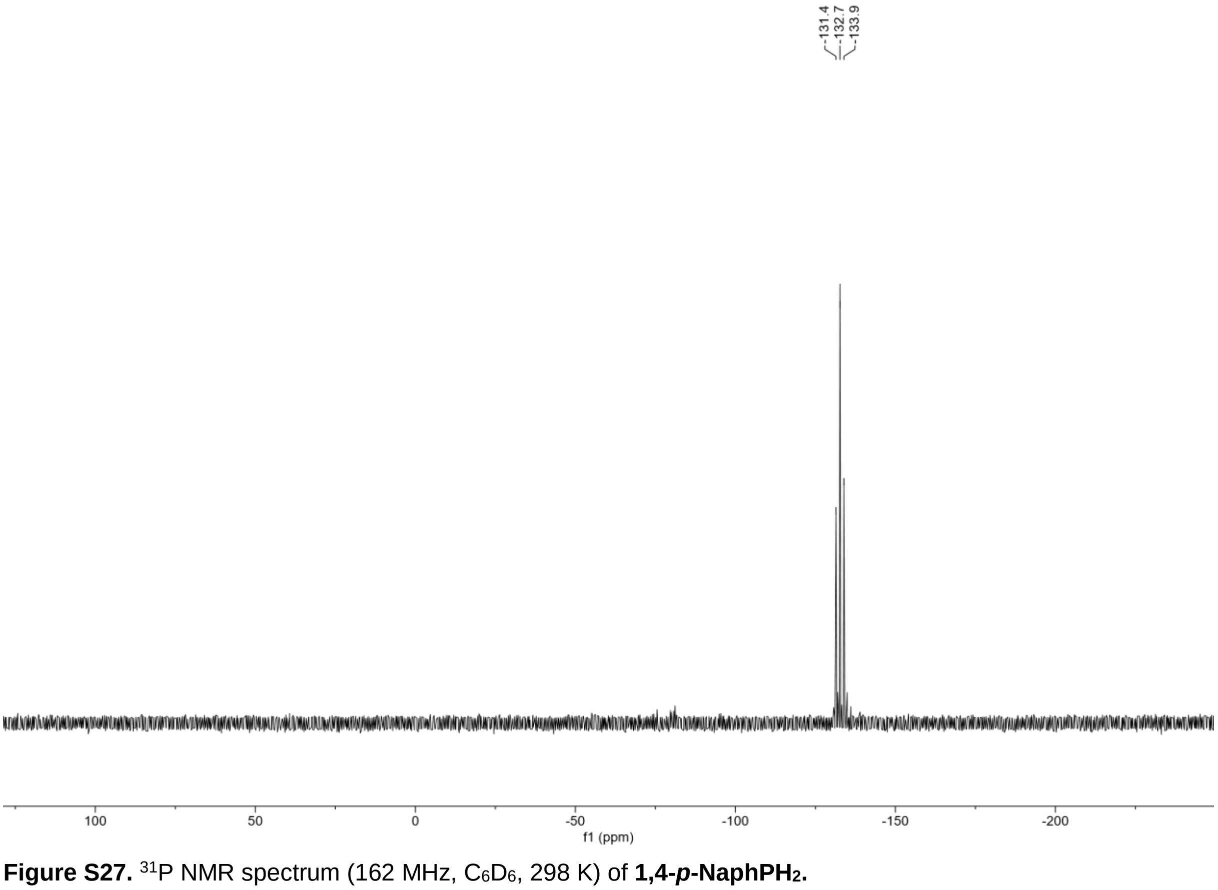

Figure S27. ^{31}P NMR spectrum (162 MHz, C_6D_6, 298 K) of **1,4-*p*-NaphPH$_2$.**

Synthesis of 1,4-(^{iPr}NHC·P)$_2$-naphthylene, 4.

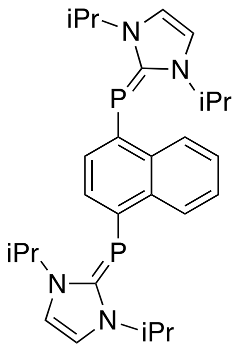

The compound was obtained using the general procedure 2, using 1,4-*p*-NaphPH$_2$ (285 mg, 1.48 mmol), ^{iPr}NHC (0.91 mL, 5.92 mmol). Compound **4** was isolated as a dark red solid (182 mg, 25 %)

Dark red crystals, suitable for SC-XRD analysis could be obtained from a concentrated solution of **4** in toluene, layered with *n*-heptane.

^{1}H NMR (400 MHz, C_6D_6, 298 K): δ = 0.84 (d, $^{3}J_{HH}$ = 6.7 Hz, 24H, NHC-^{iPr}CH_3), 4.96 (heptd, J_{HH} = 6.7, 4.1 Hz, 4H, NHC-^{iPr}C*H*), 6.16 (s, 4H, NHC-C*H*), 7.36 (dt, J_{HH} = 6.6, 3.3 Hz, 2H, Ar*H*), 7.79 (dd, J_{HH} = 3.9, 2.5 Hz, 2H, Ar*H*), 9.22 (dt, J_{HH} = 6.6, 3.4 Hz, 2H, Ar*H*).

^{13}C{^{1}H} NMR (101 MHz, C_6D_6, 298 K): δ = 21.8 (NHC-*C*H$_3$), 49.3 (d, $^{3}J_{CP}$ = 11.0 Hz, NHC-^{iPr}*C*H), 114.8 (d, $^{3}J_{CP}$ = 3.0 Hz, NHC-*C*H), 124.6 (Ar*C*), 129.9 (d, J_{CP} = 19.8 Hz, Ar*C*), 131.8 (dd, J_{CP} = 13.2, 3.6 Hz, Ar*C*), 137.3 (d, J_{CP} = 11.7 Hz, Ar*C*), 141.2 (d, $^{1}J_{CP}$ = 50.6 Hz, Ar*C*), 168.6 (d, $^{1}J_{CP}$ = 105.9 Hz, NHC-*C*).

^{31}P{^{1}H} NMR (162 MHz, C_6D_6, 298 K): δ = - 69.3 (s, ^{iPr}NHC-*P*).

Anal.calcd. for $C_{30}H_{48}N_4P_2$: C, 68.01%; H, 7.75%; N, 10.72%; found: C, 68.45%; H, 7.64%; N, 11.19%.

MS/LIFDI-HRMS found (calcd.) m/z: 492.2513 (492.2569) for $[M]^+$.

λ_{max} (tol), nm (ε, $Lmol^{-1}$ cm^{-1}): 363 (8000), 523 (15000).

λ_{max} (THF), nm (ε, $Lmol^{-1}$ cm^{-1}): 364 (8300), 523 (15400).

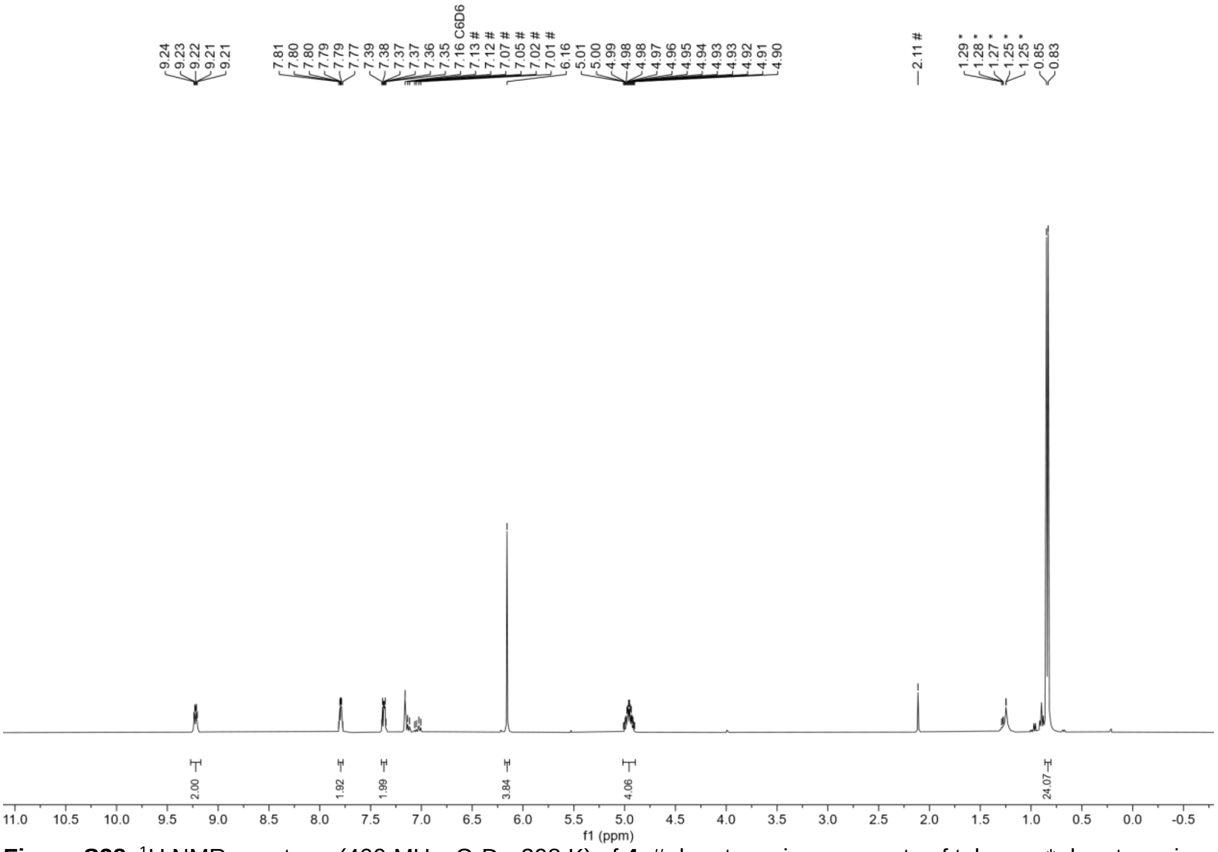

Figure S28. ^1H NMR spectrum (400 MHz, C_6D_6, 298 K) of **4**. # denotes minor amounts of toluene. * denotes minor amounts of *n*-heptane.

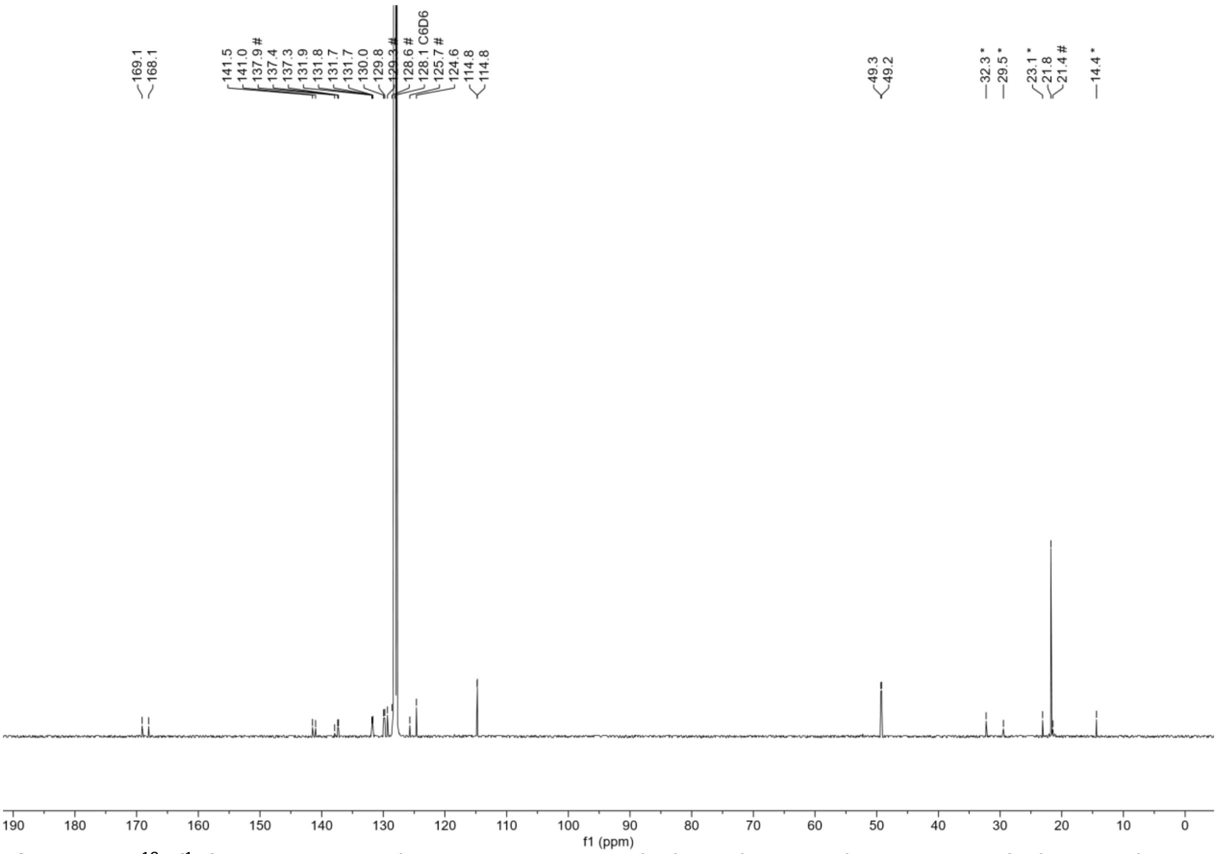

Figure S29. $^{13}C\{^1H\}$ NMR spectrum (101 MHz, C_6D_6, 298 K) of **4**. # denotes minor amounts of toluene. * denotes minor amounts of *n*-heptane.

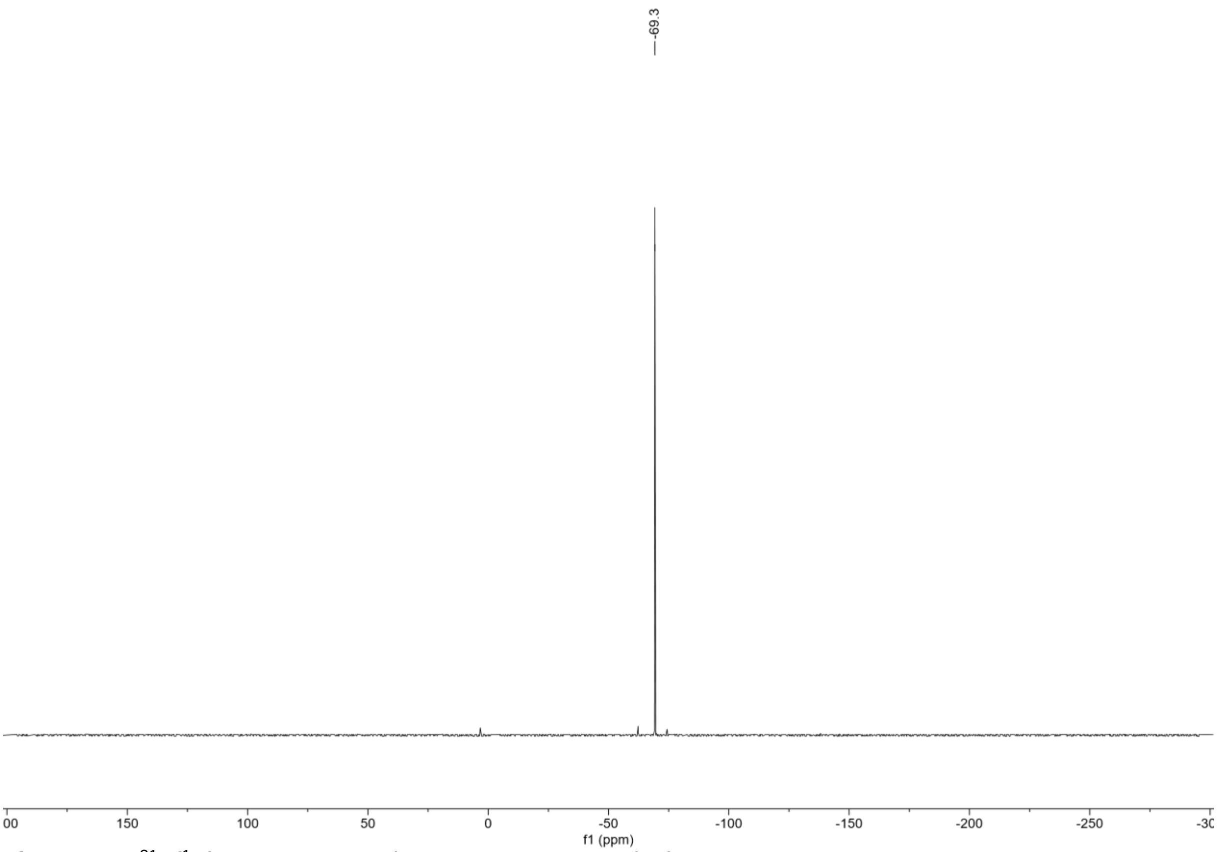

Figure S30. $^{31}P\{^{1}H\}$ NMR spectrum (162 MHz, C_6D_6, 298 K) of **4**.

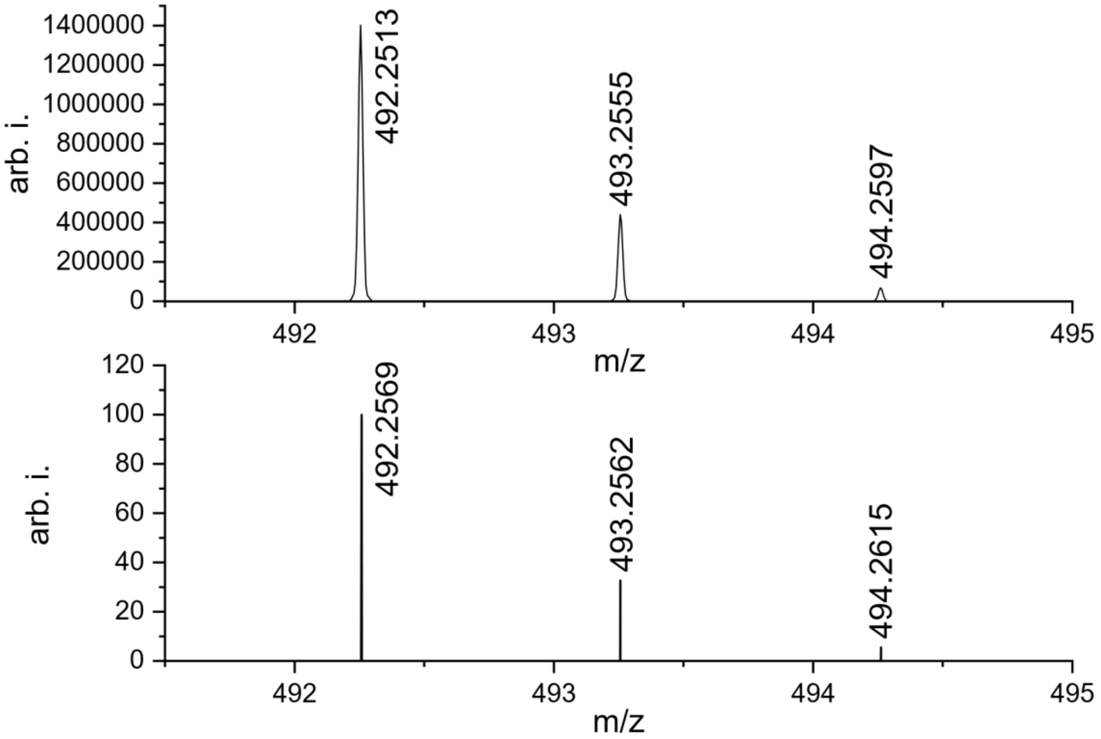

Figure S31. Cutout from LIFDI/MS of **4**, Top: found MS for [**4**]$^+$; Bottom: Calculated MS spectrum of [**4**]$^+$.

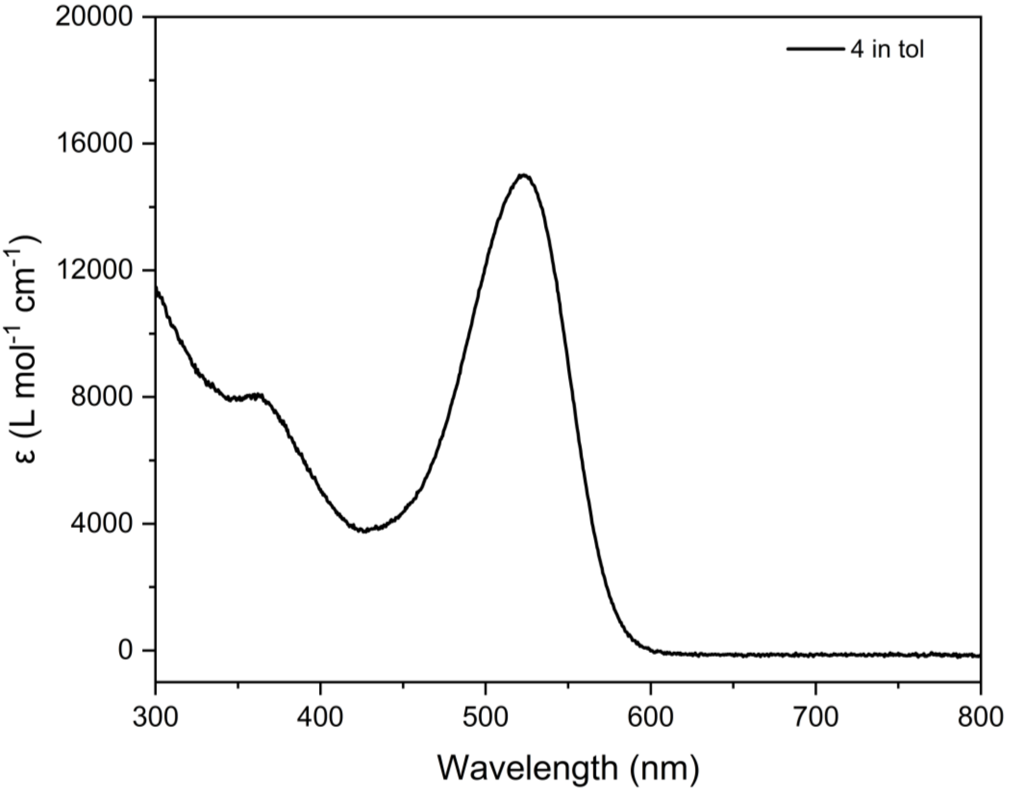

Figure S32. UV/vis spectrum of a 5x10^{-5} M solution of **4** in toluene at ambient temperature.

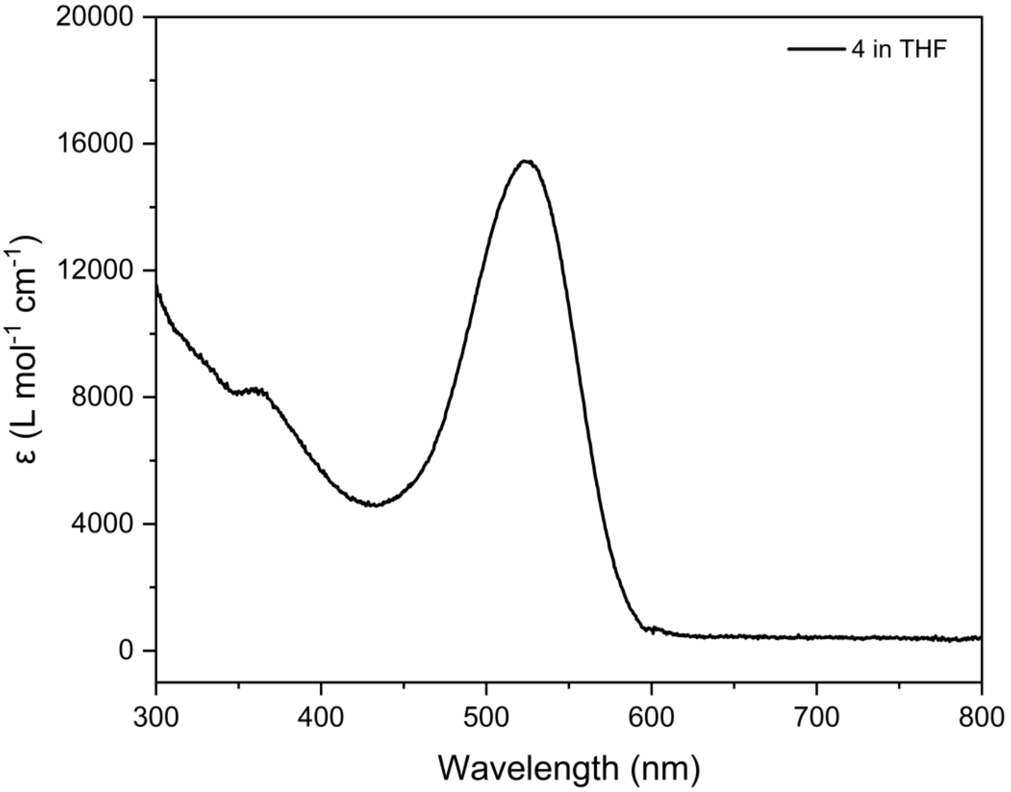

Figure S33. UV/vis spectrum of a 5x10^{-5} M solution of **4** in THF at ambient temperature.

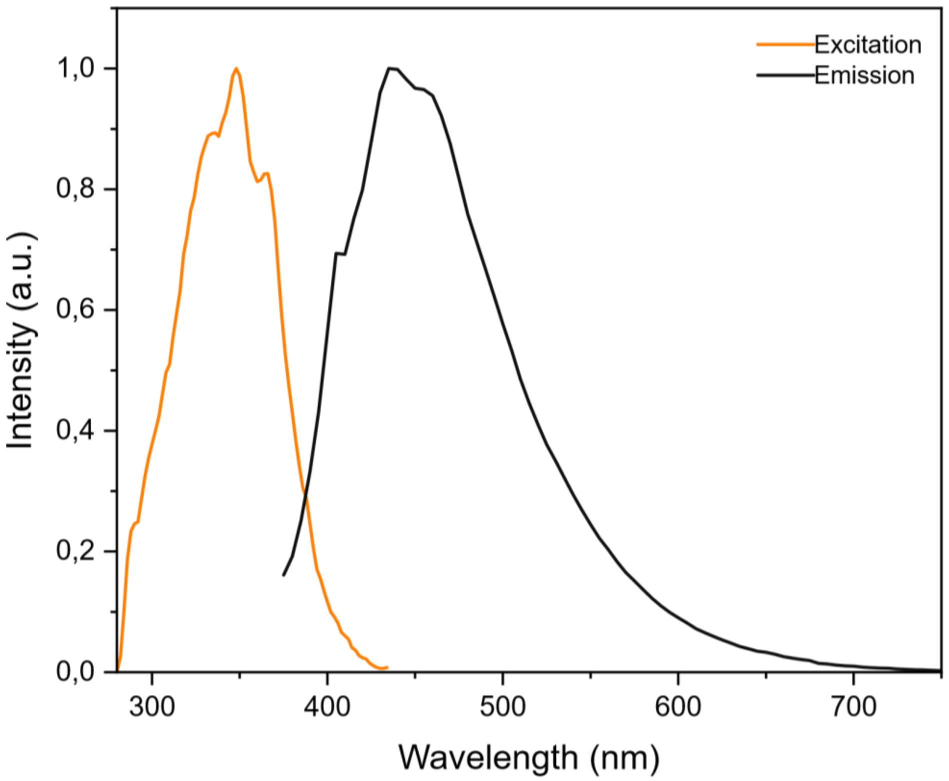

Figure S34. Orange: Excitation spectrum of **4** in toluene; Black: Fluorescence emission spectrum of **4** in toluene excited at 368 nm.

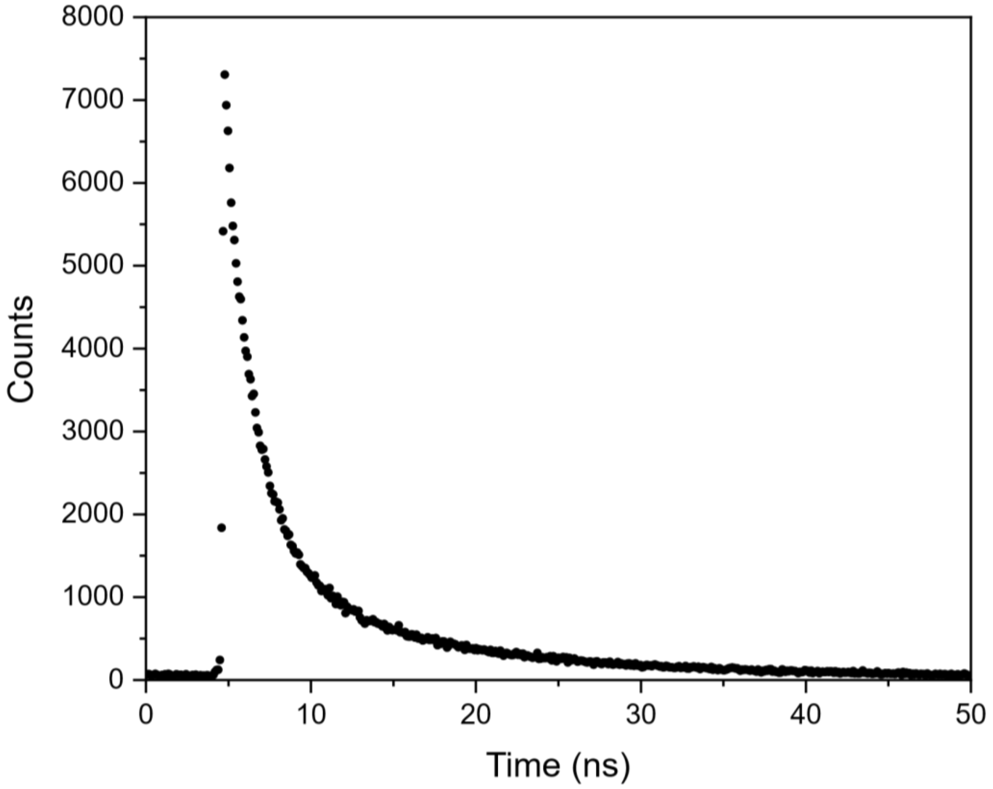

Figure S35. Fluorescence lifetime measurement of **4** in toluene.

Synthesis of 9-(^{iPr}NHC·P)-anthracene, 5.

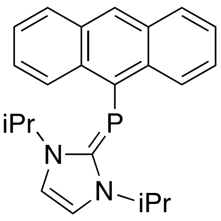

9-(PCl_2)-anthracene[2] (542 mg, 1.94 mmol) was dissolved in toluene (40 mL) and ^{iPr}NHC (0.30 mL, 1.94 mmol) was added dropwise, which immediately resulted in the precipitation of a green solid. The reaction mixture was stirred for 3 h and subsequently filtered. The green precipitate was washed with *n*-hexane (3 x 10 mL) and dried *in vacuo*. Activated Mg powder (59 mg, 2.42 mmol) and THF (40 mL) were then added, and the suspension stirred at RT for 16 h. All volatiles were subsequently removed *in vacuo*, the product extracted with toluene (3x 30 mL), and the supernatant filtered. All volatiles were again removed *in vacuo* to give **5** as a blue solid (350 mg, 50%).

Blue crystals, suitable for SC-XRD analysis could be obtained from a concentrated solution of **5** in toluene layered with *n*-pentane.

^{1}H NMR (400 MHz, C_6D_6, 298 K): δ = 0.66 (d, $^3J_{HH}$ = 6.7 Hz, 12H, NHC-^{iPr}C*H*$_3$), 4.62 (heptd, J_{HH} = 6.7, 4.0 Hz, 2H, NHC-^{iPr}C*H*), 6.04 (s, 2H, NHC-C*H*), 7.22 - 7.36 (m, 4H, Ar*H*), 7.87 (d, $^3J_{HH}$ = 1.7 Hz, 1H, Ar*H*), 7.88 - 7.90 (m, 1H, Ar*H*), 8.13 (s, 1H, Ar*H*), 9.49 - 9.57 (m, 2H, Ar*H*).

^{13}C{^{1}H} NMR (101 MHz, C_6D_6, 298 K): δ = 21.5 (NHC-*C*H$_3$), 49.2 (d, $^3J_{CP}$ = 11.0 Hz, NHC-^{iPr}*C*H),), 114.5 (d, $^3J_{CP}$ = 3.4 Hz, NHC-*C*H), 124.2 (d, J_{CP} = 2.1 Hz, Ar*C*), 124.6 (d, J_{CP} = 1.8 Hz, Ar*C*), 125.2 (Ar*C*), 129.4 (Ar*C*), 130.8 (d, J_{CP} = 16.5 Hz, Ar*C*), 132.7 (Ar*C*), 136.0 (d, J_{CP} = 8.1 Hz, Ar*C*), 145.0 (d, $^1J_{CP}$ = 59.9 Hz, Ar*C*), 168.3 (d, $^1J_{CP}$ = 106.6 Hz, NHC-*C*).

^{31}P{^{1}H} NMR (162 MHz, C_6D_6, 298 K): δ = - 85.3 (s, ^{iPr}NHC-*P*).

Anal.calcd. for $C_{23}H_{25}N_2P$: C, 76.64%; H, 6.99%; N, 7.77%; found: C, 77.32%; H, 6.99%; N, 7.22%.

MS/LIFDI-HRMS found (calcd.) m/z: 360.1736 (360.1753) for $[M]^+$.

λ_{max} (tol), nm (ε, $Lmol^{-1}$ cm^{-1}): 360 (11100), 376 (12100), 395 (9400), 604 (6600).

λ_{max} (THF), nm (ε, $Lmol^{-1}$ cm^{-1}): 360 (9400), 377 (10300), 393 (8200), 606 (6100).

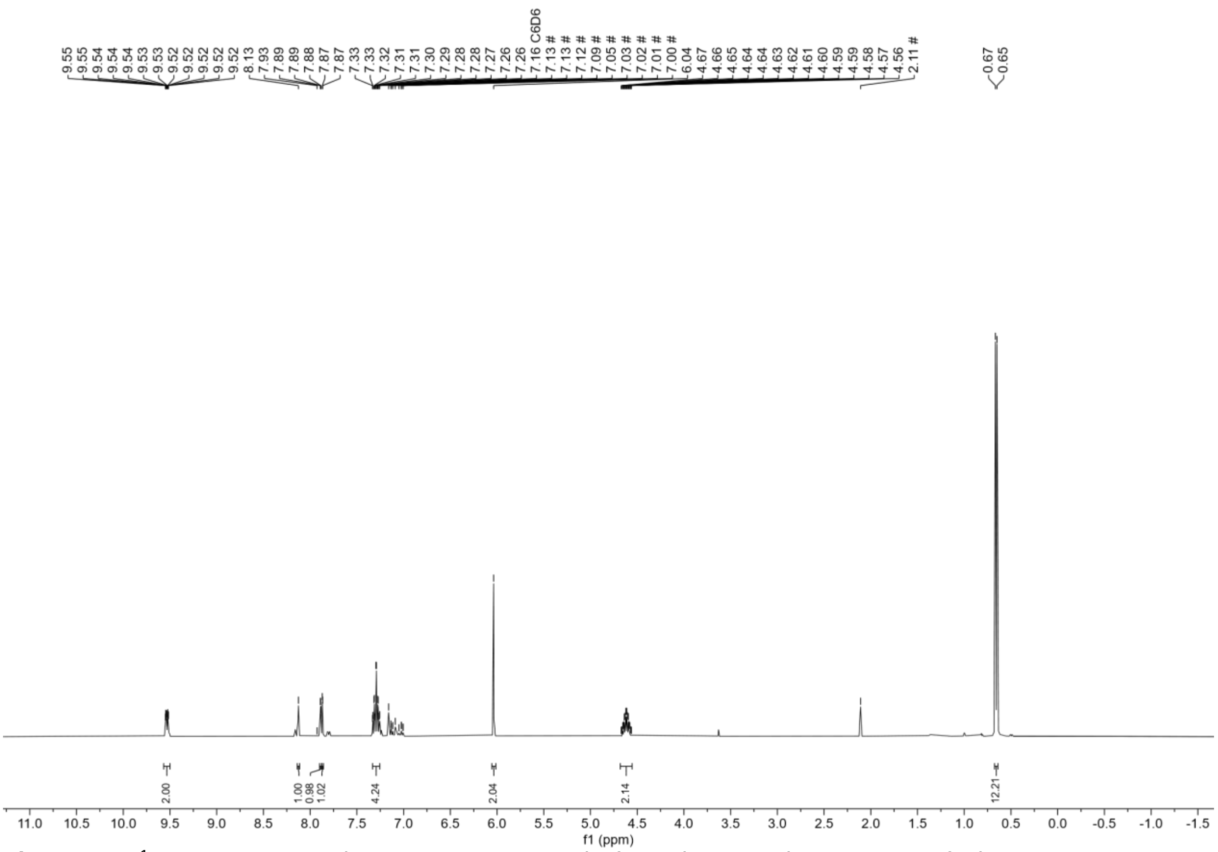

Figure S36. ^{1}H NMR spectrum (400 MHz, C_6D_6, 298 K) of **5**. # denotes minor amounts of toluene.

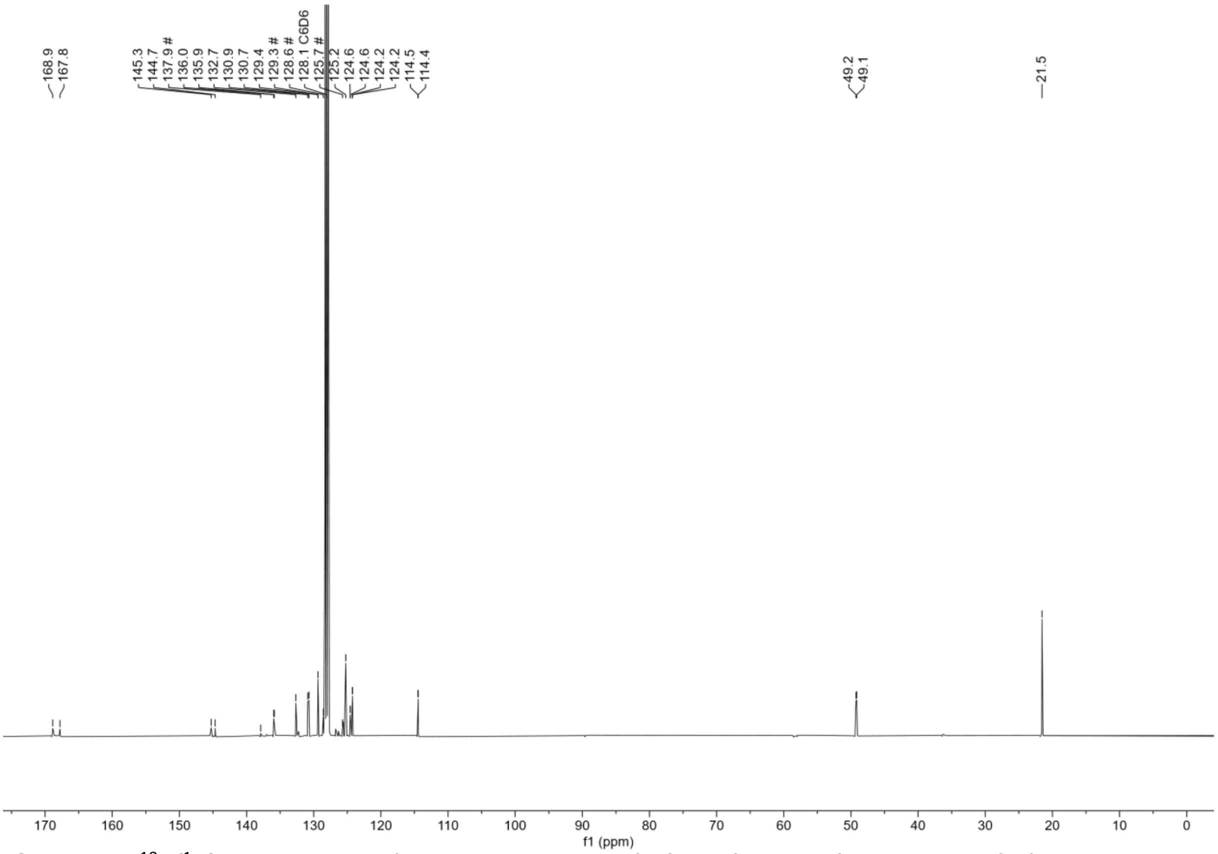

Figure S37. $^{13}C\{^{1}H\}$ NMR spectrum (101 MHz, C_6D_6, 298 K) of **5**. # denotes minor amounts of toluene.

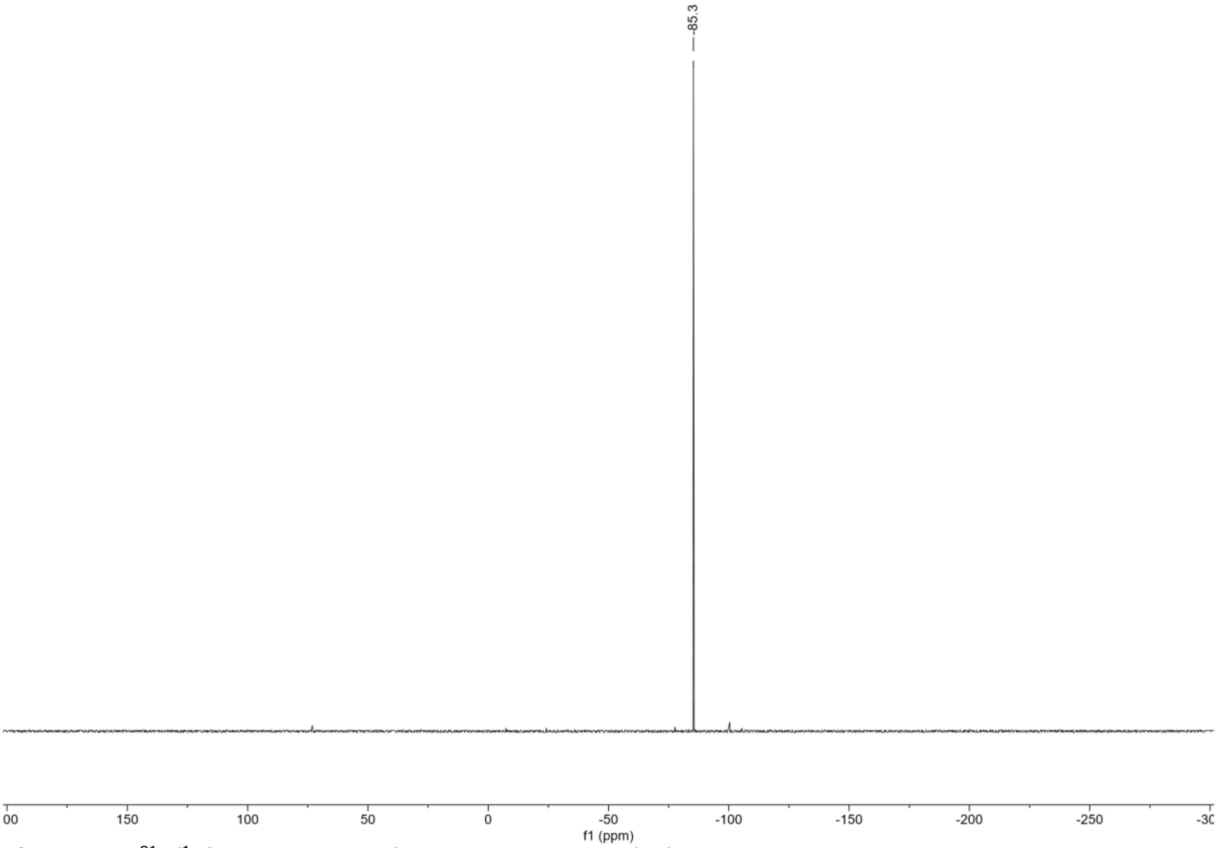

Figure S38. $^{31}P\{^{1}H\}$ NMR spectrum (162 MHz, C_6D_6, 298 K) of **5**.

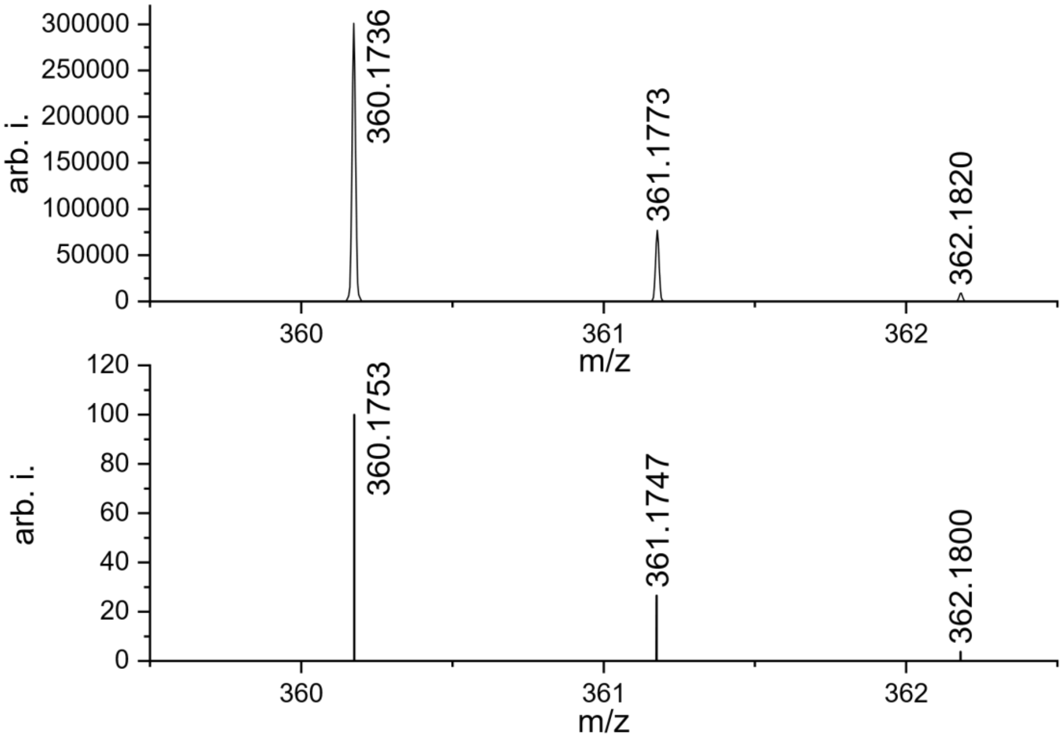

Figure S39. Cutout from LIFDI/MS of **5**, Top: found MS for [**5**]$^{+}$; Bottom: Calculated MS spectrum of [**5**]$^{+}$.

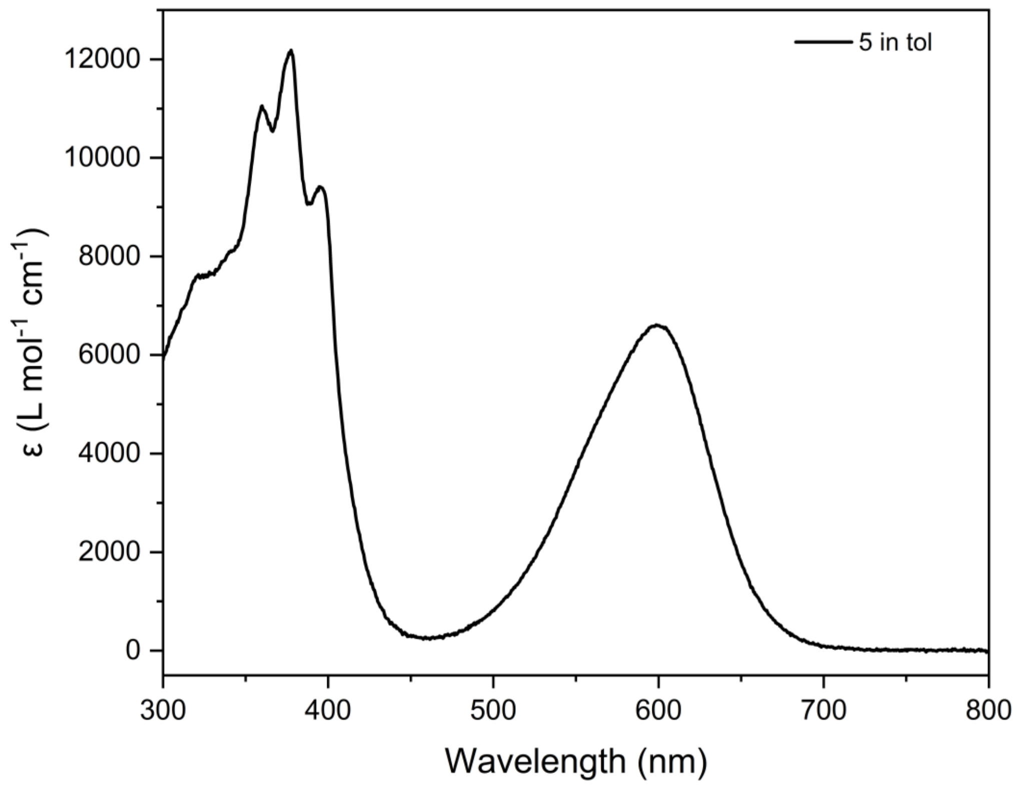

Figure S40. UV/vis spectrum of a 1x10^{-4} M solution of **5** in toluene at ambient temperature.

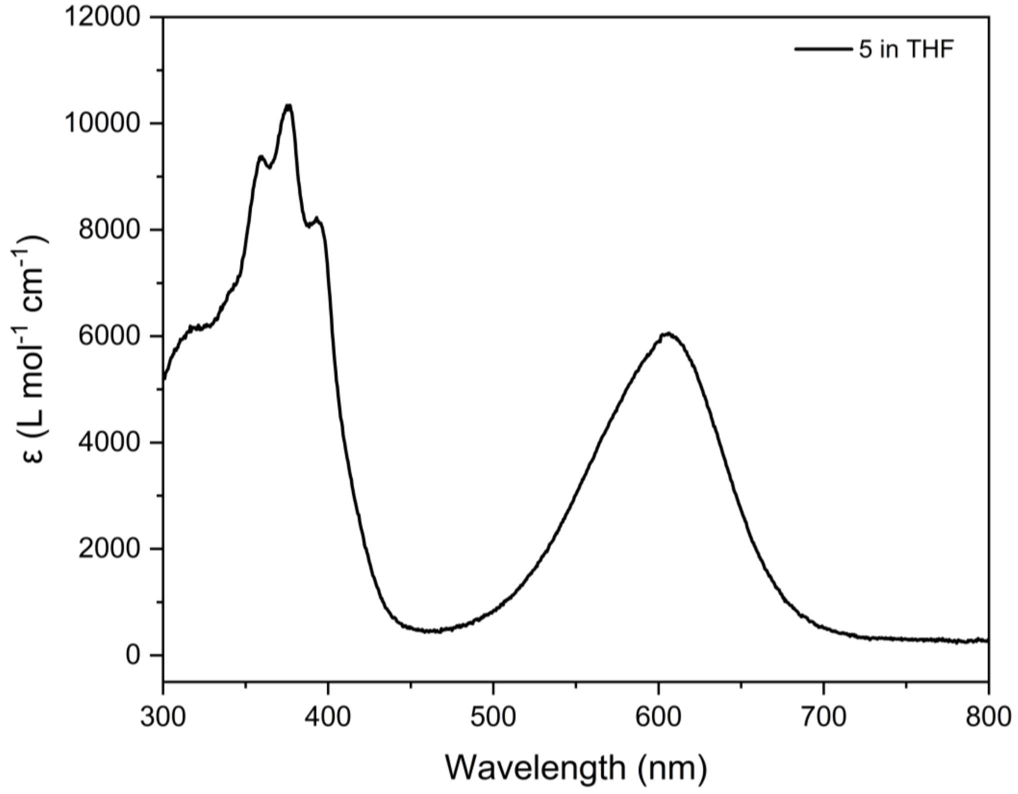

Figure S41. UV/vis spectrum of a 1x10^{-4} M solution of **5** in THF at ambient temperature.

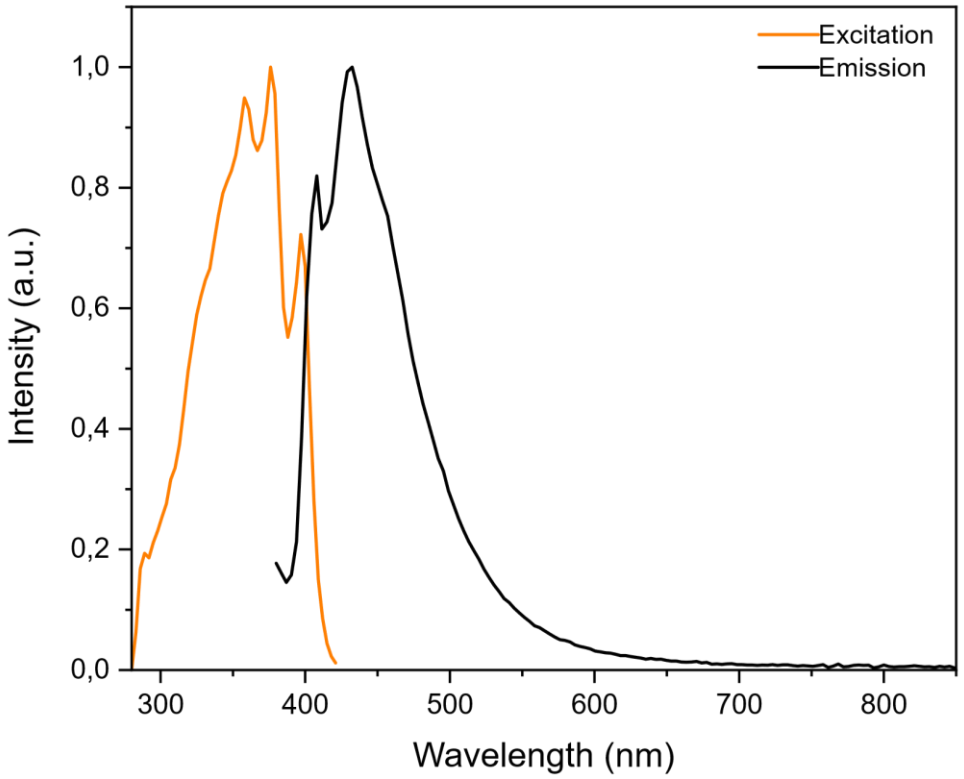

Figure S42. Orange: Excitation spectrum of **5** in toluene; Black: Fluorescence emission spectrum of **5** in toluene excited at 376 nm.

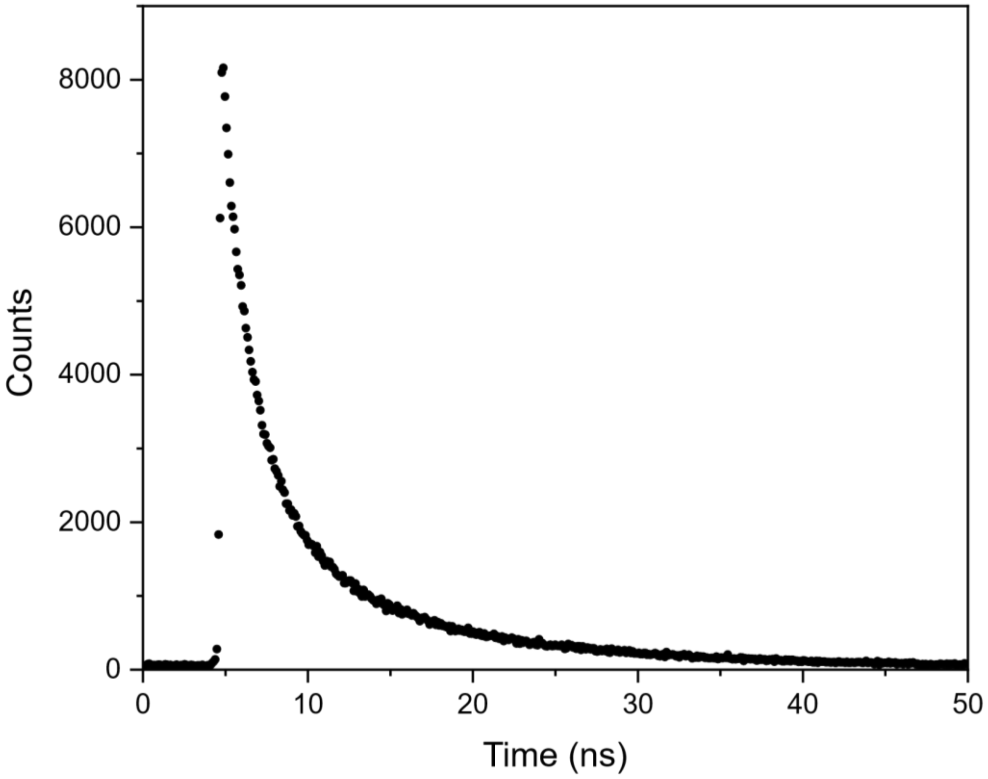

Figure S43. Fluorescence lifetime measurement of **5** in toluene.

Synthesis of 9,10-(^{iPr}NHC·P)$_2$-anthracene, 6.

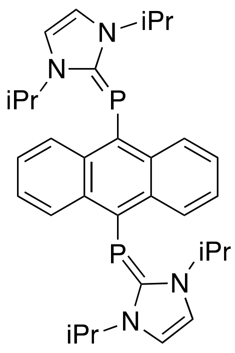

To a solution of 9,10-(PCl_2)$_2$-anthracene[2] (1.13 g, 2.97 mmol) in toluene (70 mL), ^{iPr}NHC (0.90 g, 5.95 mmol) was added dropwise via syringe, which immediately led to the formation of a green precipitate. After stirring for 3 h, the reaction mixture was filtered, the precipitate washed with pentane (3 x 15 mL), and dried *in vacuo* to give a green powder. Subsequently, Mg powder (181 mg, 7.45 mmol) was added, and the solid mixture dispersed in THF (70 mL). The suspension was stirred at RT for 16 h forming a blue-green solution above a grey precipitate. All volatiles were removed *in vacuo*, the product was extracted with toluene (100 mL), and filtered, giving a deep blue-green solution. All volatiles were again removed from the filtrate to give a blue-green solid (856 mg, 53 %).

Dark blue crystals, suitable for SC-XRD analysis could be obtained from a concentrated solution of **6** in THF layered with *n*-pentane.

^{1}H NMR (400 MHz, C_6D_6, 298 K): δ =0.68 (d, $^3J_{HH}$ = 6.8 Hz, 24H, NHC-^{iPr}CH_3), 4.71 (tt, J_{HH} = 10.4, 5.2 Hz, 4H, NHC-^{iPr}C*H*), 6.06 (s, 4H, NHC-C*H*), 7.34 (dd, J_{HH} = 6.8, 3.3 Hz, 4H, Ar*H*), 9.67 - 9.75 (m, 4H, Ar*H*).

^{31}P{^{1}H} NMR (162 MHz, C_6D_6, 298 K): δ = = - 84.1 (s, ^{iPr}NHC-*P*).

Anal.calcd. for $C_{32}H_{40}N_4P_2$: C, 70.83%; H, 7.43%; N, 10.32%; found: C, 66.37%; H, 7.47%; N, 8.91%.

N.B. After several runs, consistently low C and N values were found for this compound, which we believe to be due to incomplete combustion due to the presence of phosphorous.

MS/LIFDI-HRMS found (calcd.) m/z: 542.2700 (542.2725) for $[M]^+$.

λ_{max} (tol), nm (ε, Lmol^{-1} cm^{-1}): 334 (13000), 378 (14400), 647 (12100).

λ_{max} (THF), nm (ε, Lmol^{-1} cm^{-1}): 332 (14100), 378 (15800), 653 (14900).

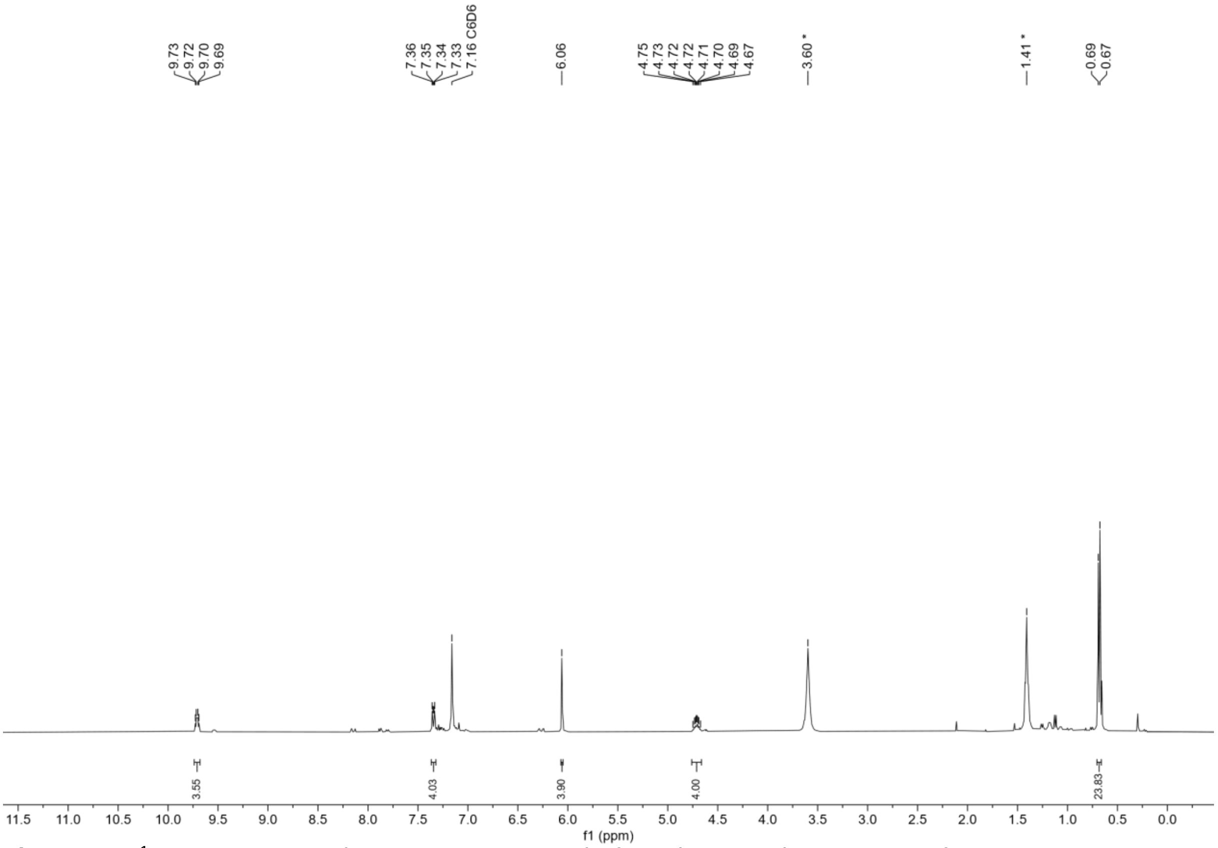

Figure S44. ^{1}H NMR spectrum (400 MHz, C_6D_6, 298 K) of **6**. * denotes minor amounts of THF.

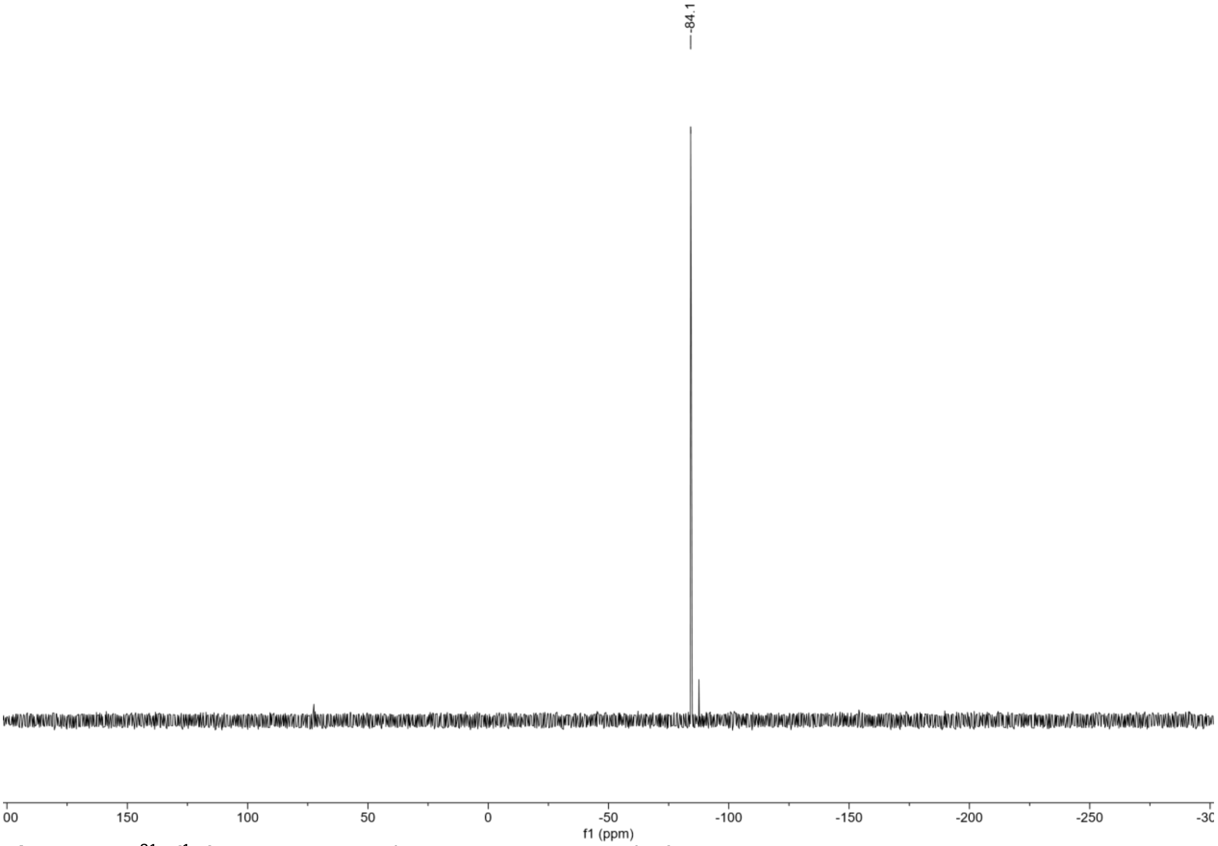

Figure S45. $^{31}P\{^{1}H\}$ NMR spectrum (162 MHz, C_6D_6, 298 K) of **6**.

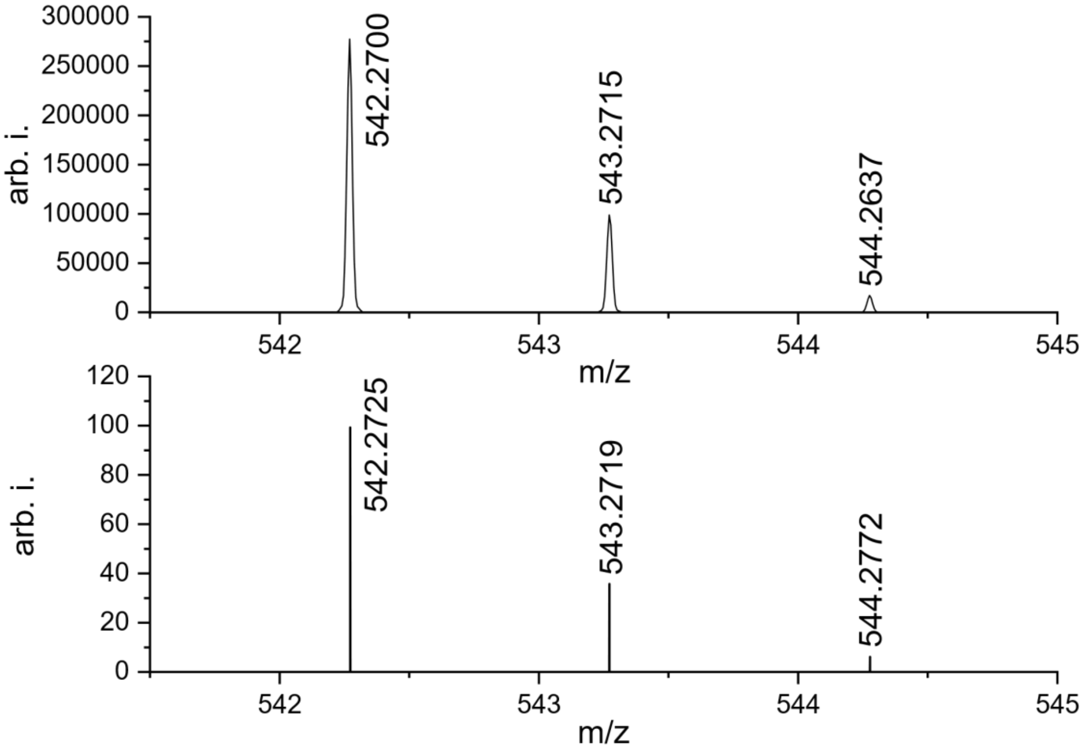

Figure S46. Cutout from LIFDI/MS of **6**, Top: found MS for [**6**]$^+$; Bottom: Calculated MS spectrum of [**6**]$^+$.

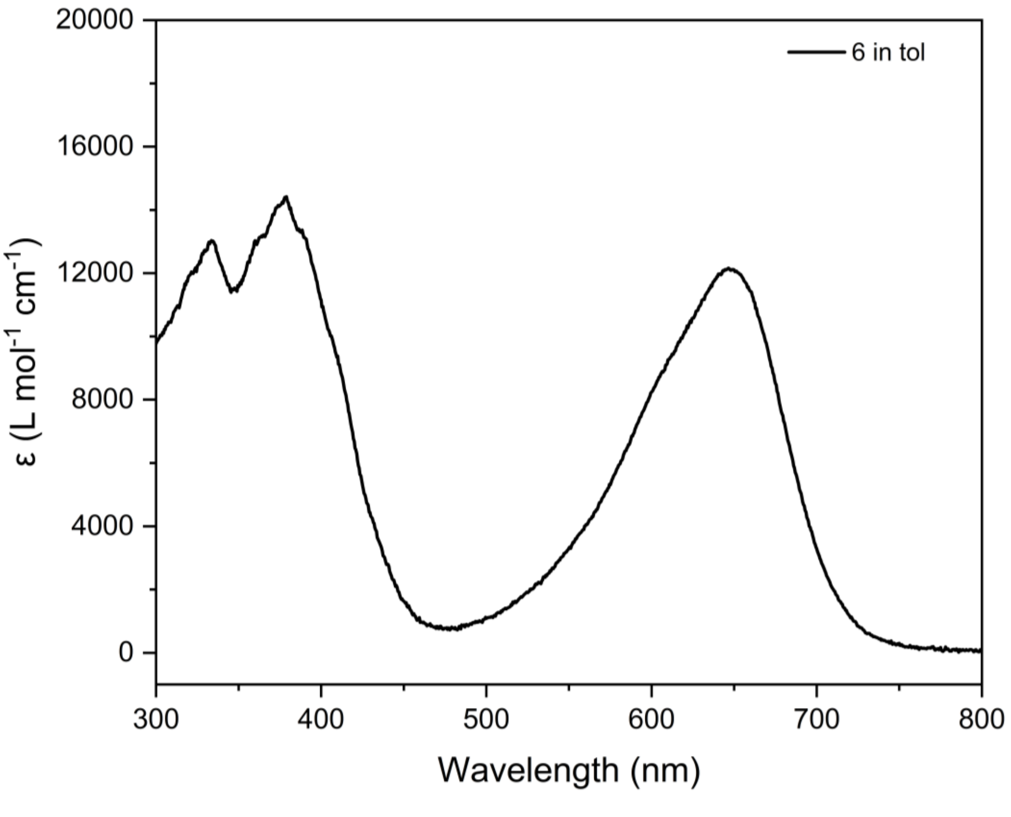

Figure S47. UV/vis spectrum of a 5x10^{-5} M solution of **6** in toluene at ambient temperature.

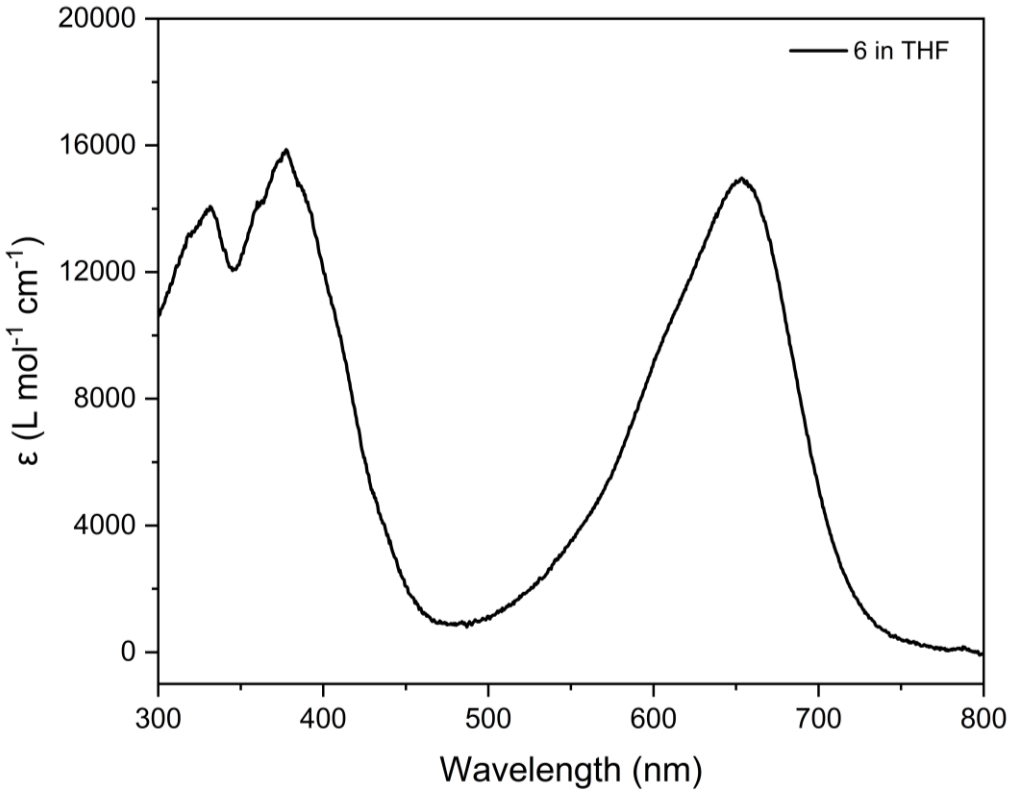

Figure S48. UV/vis spectrum of a 5x10^{-5} M solution of **6** in THF at ambient temperature.

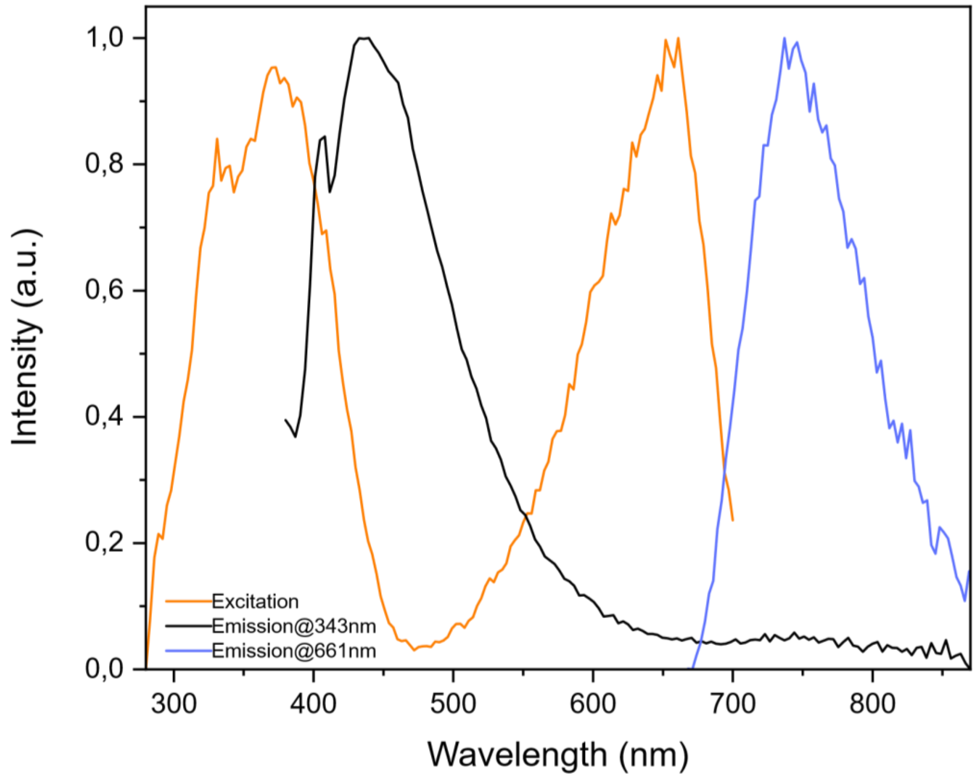

Figure S49. Orange: Excitation spectrum of **6** in toluene; Black: Fluorescence emission spectrum of **6** in toluene excited at 343 nm; Blue: Fluorescence emission spectrum of **6** in toluene excited at 661 nm.

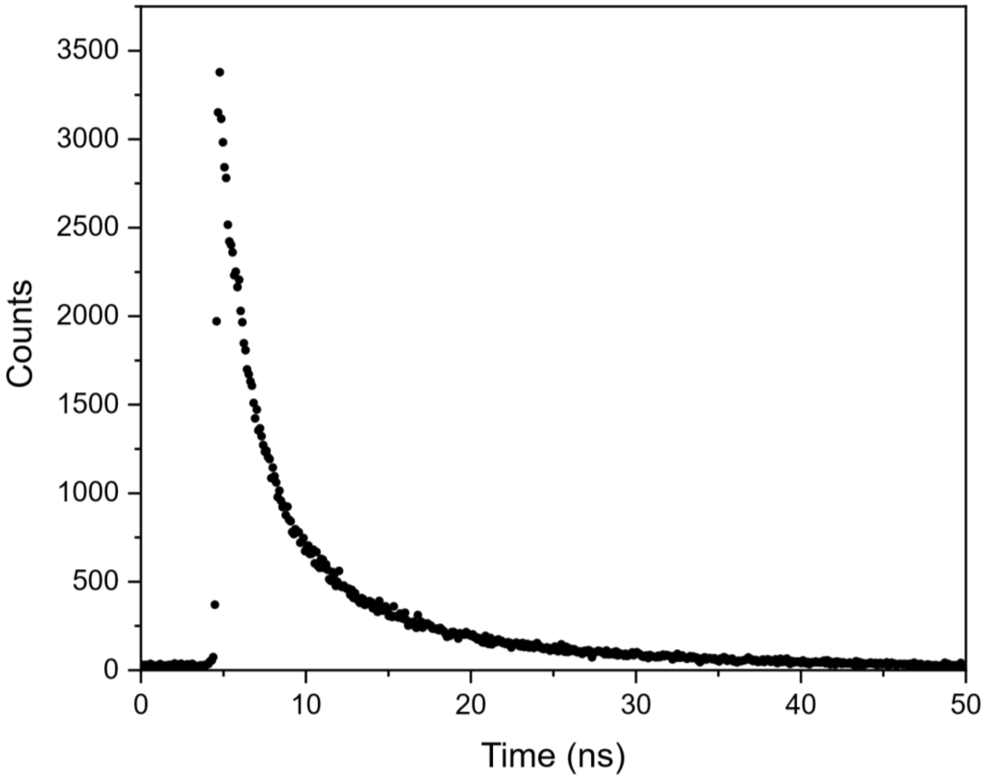

Figure S50. Fluorescence lifetime measurement of **6** in toluene.

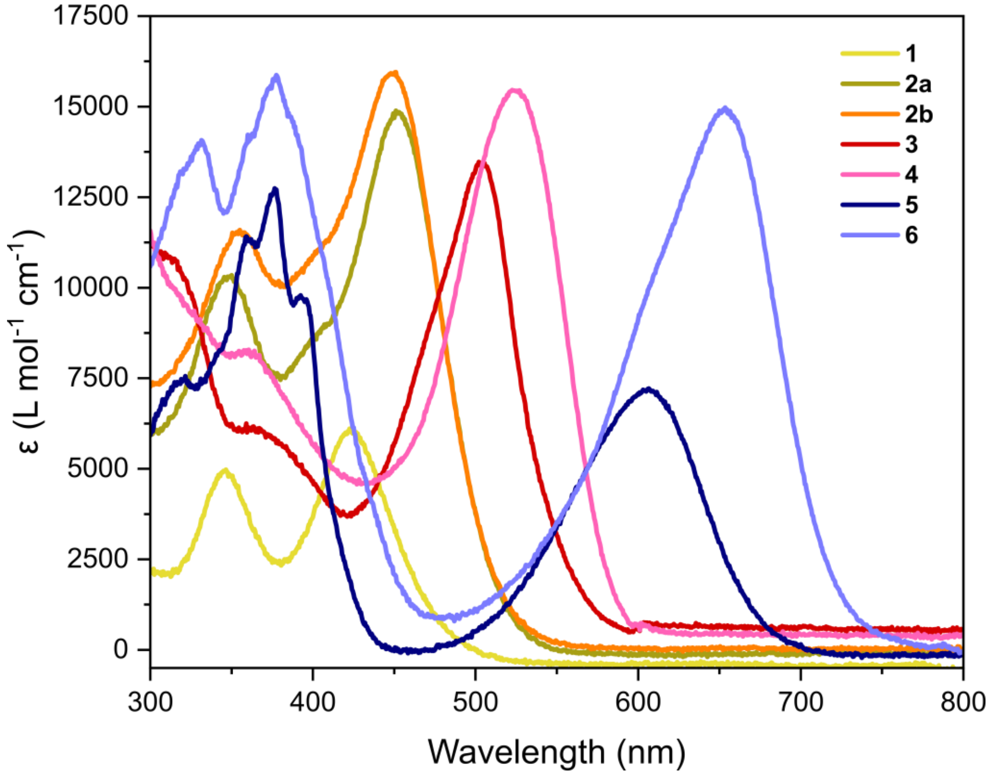

Figure S51. A plot combining all above UV/vis data for compounds **1**-**6** as solutions THF.

Fluorescence measurements

Mean fluorescence lifetime was calculated directly from the histogram, according to the following formula:[8]

$$\tau_H = \frac{\sum_{i=p}^{n}(N_i - noise)t_i}{\sum_{i=p}^{n}(N_i - noise)} - t_p$$

$\boldsymbol{\tau_H}$ Mean fluorescence lifetime

$\boldsymbol{N_i}$ number of detected photons in the *i*-th channel at time t_i

$\boldsymbol{n}$ total number of channels in the histogram

$\boldsymbol{p}$ channel with the highest number of detected photons at time t_p

$\boldsymbol{noise}$ was determined by averaging counts in the noise-domain of the histogram

Table S1. Summary of photophysical data in toluene at 298 K.

	λ_{max} ex [nm]	λ_{max} em [nm]	Δ_{Stokes} [cm^{-1}]	Φ [%]	τ [ns]
**2b**	315	370[a]	4719	-	-
**3**	317	372[a]	4664	-	-
**4**	348	435[b]	5747	-	6.0
**5**	376	432[e]	3448	-	6.3
**6**	370	436[c]	4091	2.07	5.9
	661	746[d]	1724	0.37	-

a) excited at 317 nm, b) excited at 368 nm, c) excited at 343 nm, d) excited at 661 nm, e) excited at 376 nm

Stability measurements

To test the stability of these NHC-phosphanylidene systems, a $5x10^{-5}$ M solution of **4** in toluene was prepared in a glovebox. The solution was transferred to cuvettes with a cap. A UV/vis spectrum with a closed cap was taken and then the cap was removed. The decomposition was monitored until complete decolouration.

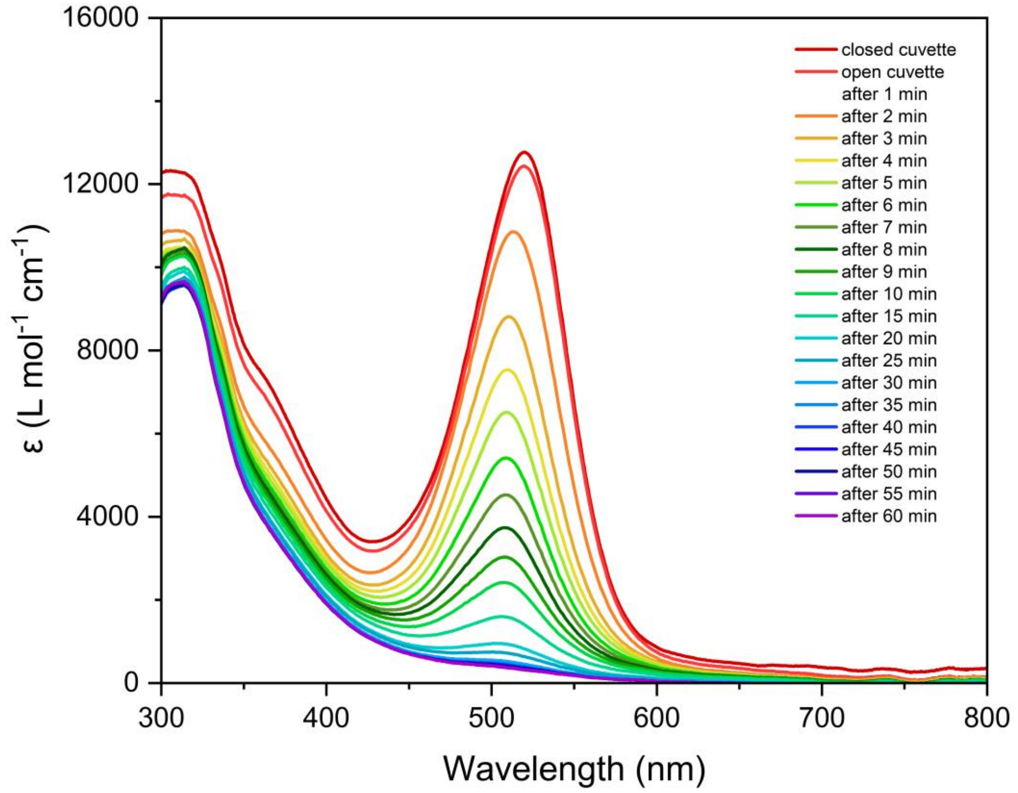

Figure S52. UV/vis spectra of **4** in toluene at ambient temperature exposed to air.

Additionally, **4** was exposed to air as a crystalline solid. Decomposition was monitored *via* ^{1}H NMR spectroscopy in C_6D_6 with 0.05 mmol mL^{-1} mesitylene as internal standard.

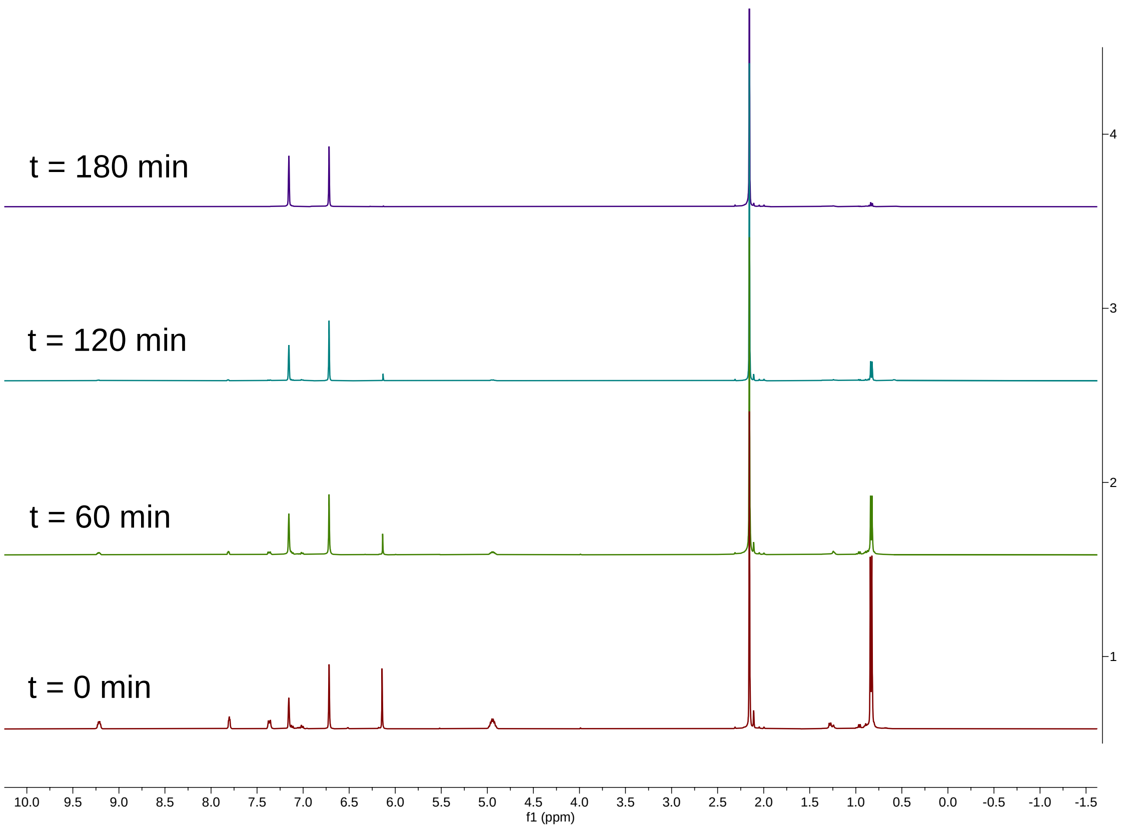

Figure S53. Stacked ^{1}H NMR spectra (400 MHz, C_6D_6, 298 K) of **4** exposed to air as a crystalline solid. NMR spectra taken in C_6D_6 with mesitylene as an internal standard.

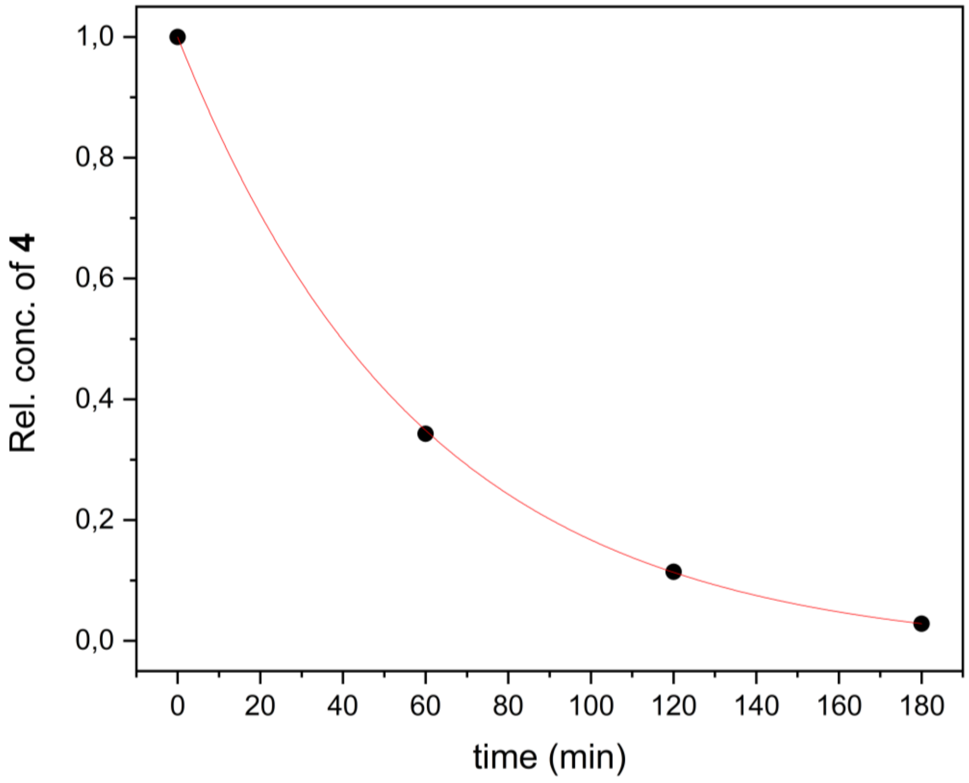

Figure S54. Exponential fit of relative concentration of **4** at t_x to the concentration of **4** at t_0 plotted against time (t_x).

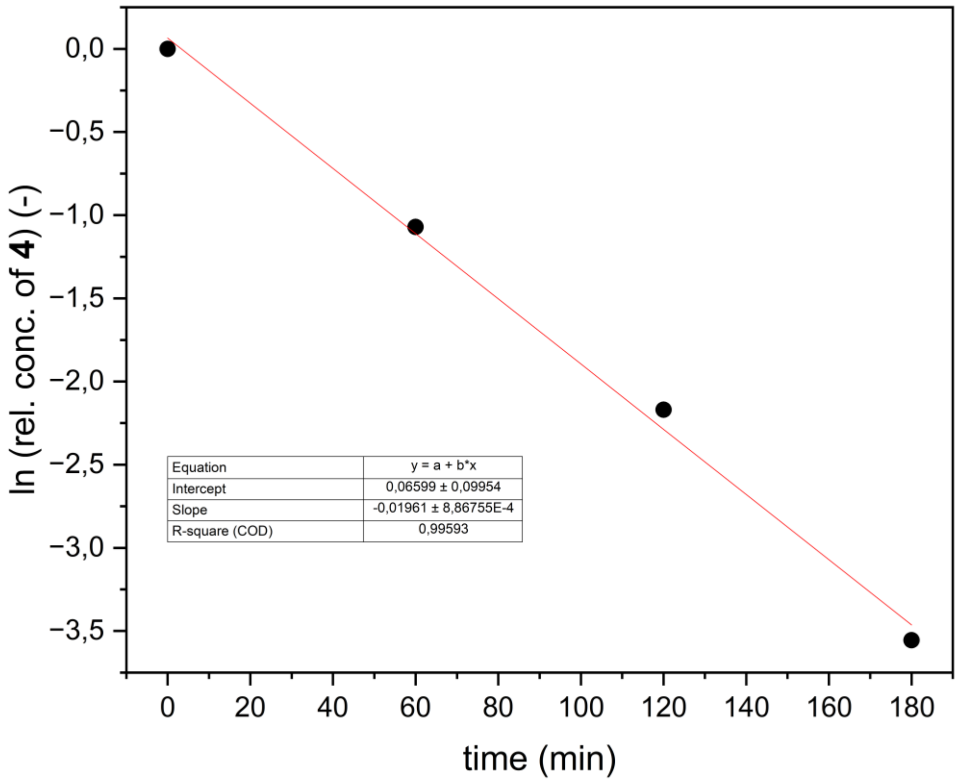

Figure S55. First order time law linearization of Fig. S53.

2. X-ray crystallographic details

Single crystals of **2b**, **3**, **4**, **5**, **6**, and **(NapthP)$_4$** suitable for X-ray structural analysis were mounted in perfluoroalkyl ether oil on a nylon loop and positioned in a 150 K cold N_2 gas stream. Data collection was performed with a STOE StadiVari diffractometer (MoKα radiation) equipped with a DECTRIS PILATUS 300K detector. Structures were solved by Direct Methods (SHELXS-97),[9] or using SHELXT-16,[10] and refined by full-matrix least-squares calculations against F^2 (SHELXL-2018).[11] The positions of the hydrogen atoms were calculated and refined using a riding model. All non-hydrogen atoms were treated with anisotropic displacement parameters. Crystal data, details of data collections, and refinements for all structures can be found in their CIF files, which are available free of charge via www.ccdc.cam.ac.uk/data_request/cif, and are summarized in Table S2-S3.

Table S2. Summary or X-ray data for **2b**, **3**, and **4**.

Compound	**2b**	**3**	**4**
Empirical formula	$C_{30}H_{48}N_4P_2$	$C_{28}H_{38}N_4P_2$, C_4H_8O	$C_{28}H_{38}N_4P_2$
Formula weight	526.66	564.67	492.56
Crystal system	orthorhombic	monoclinic	trigonal
Space group	$P2_12_12_1$	$P2_1/n$	$P3_121$
a (Å)	10.455(2)	16.199(3)	13.7700(19)
b (Å)	13.310(3)	9.3270(19)	13.7700(19)
c (Å)	22.837(5)	21.549(4)	16.430(3)
α (°)	90	90	90
β (°)	90	97.55(3)	90
γ (°)	90	90	120
V (Å^3)	3178.0(11)	3227.7(11)	2698.0(9)
Z	4	4	3
ρcalc (g·cm^{-3})	1.101	1.162	0.909
μ (mm^{-1})	0.160	0.165	0.138
F(000)	1144	1216	792
T (K)	150(2)	150(2)	150(2)
Completeness to θ_{max} (%)	99.9	100.0	99.9
Reflections collected	41115	43201	14165
Unique reflections	6234	6336	3536
Rint	0.0441	0.0714	0.0531
R1 [I > 2σ(I)]	0.0336	0.0526	0.0590
wR2 (all data)	0.0874	0.1342	0.1962
CCDC No.	2537609	2537610	2537611

Table S3. Summary or X-ray data for **5**, **6**, and **(NaphthP)$_4$**.

Compound	**5**	**6**	**(NaphthP)$_4$**
Empirical formula	$C_{23}H_{25}N_2P$	$C_{32}H_{40}N_4P_2$	$C_{40}H_{28}P_4$
Formula weight	360.42	542.62	632.50
Crystal system	monoclinic	monoclinic	orthorhombic
Space group	$P2_1/n$	$P2_1/n$	$P2_12_12$
a (Å)	9.5400(19)	10.668(2)	14.290(3)
b (Å)	16.330(3)	9.955(2)	14.390(3)
c (Å)	25.360(5)	14.176(3)	7.4500(15)
α (°)	90	90	90
β (°)	91.30(3)	102.70(3)	90
γ (°)	90	90	90
V (Å^3)	3949.8(14)	1468.7(5)	1532.0(5)
Z	8	2	2
ρcalc (g·cm^{-3})	1.212	1.227	1.371
μ (mm^{-1})	0.148	0.176	0.276
F(000)	1536	580	656
T (K)	150(2)	150(2)	150(2)
Completeness to θ_{max} (%)	100.0	99.5	99.6
Reflections collected	54525	10723	5141
Unique reflections	9074	2868	2743
Rint	0.0978	0.0680	0.0340
R1 [I > 2σ(I)]	0.0668	0.0637	0.0553
wR2 (all data)	0.2019	0.1833	0.1623
CCDC No.	2537612	2537613	2537614

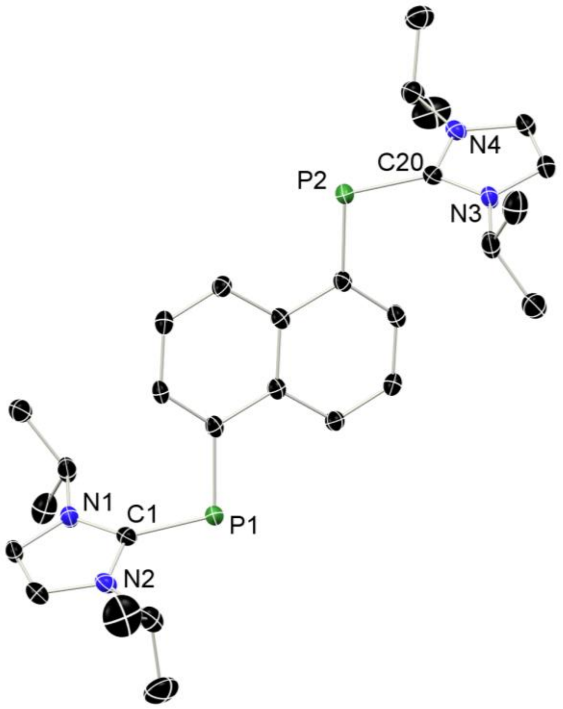

Figure S56. Molecular structure of **3**, with thermal ellipsoids at 30% probability and hydrogen atoms removed for clarity.

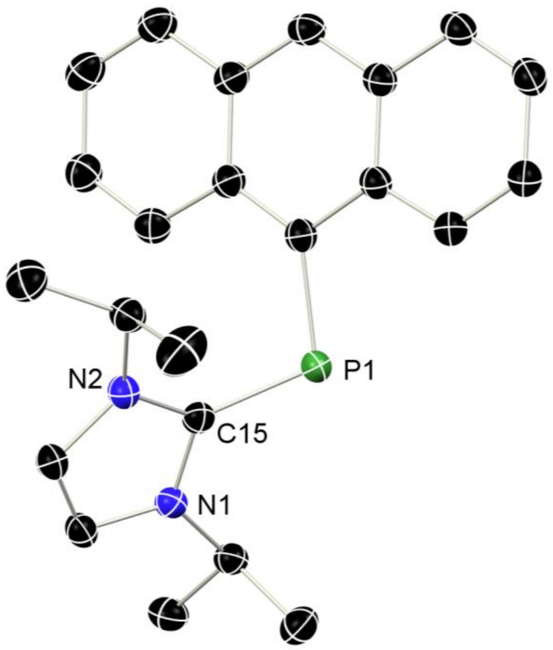

Figure S57. Molecular structure of **5**, with thermal ellipsoids at 30% probability and hydrogen atoms removed for clarity.

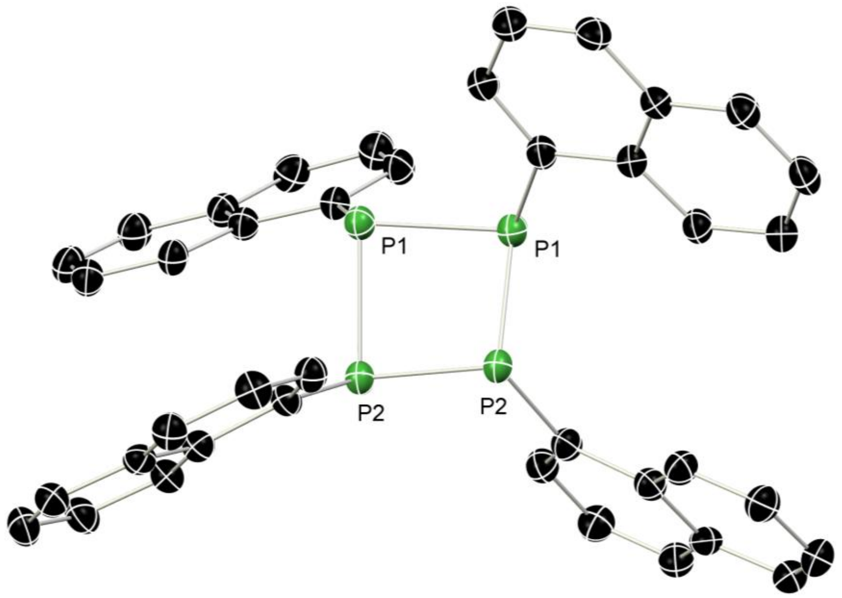

Figure S58. Molecular structure of **(NapthP)$_4$**, with thermal ellipsoids at 30% probability and hydrogen atoms removed for clarity.

3. Computational Methods

All calculations were carried out with the ORCA program package.[12,13] Density fitting techniques, also called resolution-of-identity approximation (RI)[14], were used for GGA calculations and the RIJCOSX[15] approximation was used for hybrid-GGA DFT and CASSCF calculations. Atom-pairwise dispersion corrections[16,17] were used for geometry optimisations. All geometries were obtained at the BP86-D3BJ/def2-SVP[18–20] level of theory on isolated molecules (in the gas phase). For all considered phosphanylidenes, a truncated model was used, whereby isopropyl groups of the NHC were replaced by methyl groups and all aromatic rings were used as the protio species (*i.e.* the 2,5-Me/^{i}Pr groups in **2a** and **2b** are replaced by H atoms), giving model compounds **1’-6’**. TDDFT calculations of the first 10 roots, that is, excited states, were performed at the ωB97X-D3/def2-TZVP[20,21] level of theory together with the CPCM[22] model for toluene. CASSCF calculations were carried out with the def2-SVP basis set including the complete π-system of each substituted arene and a p-type lone pair of electrons at each phosphorus center. In total, this resulted in the following active spaces: **1’** (10/8), **3’** (14/12), **4’** (14/12), **5’** (16/15), **6’** (18/16). Subsequently, the active orbitals were expressed as localised orbitals, allowing for the determination of the occupation of the phosphorus lone pairs and the individual carbon-centered p-orbitals constituting the arene system. These values are tabulated below and the respective output files are attached as a supplement which allow viewing of the orbitals.

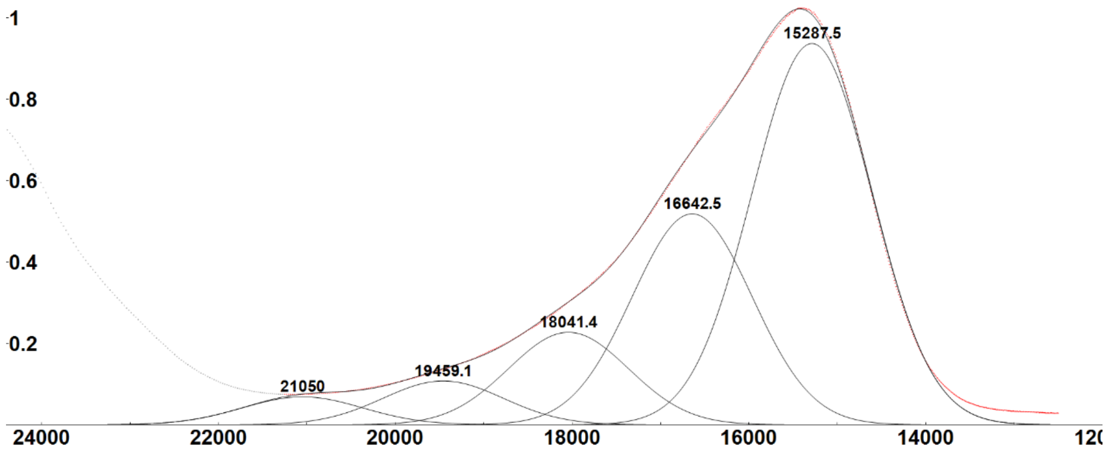

Figure S59. Spectral deconvolution of the UV-vis spectrum of **6’** in toluene represented as wavenumbers [cm^{-1}]. Black: model functions, FWHM = 800 cm^{-1}, red: experimental spectrum.

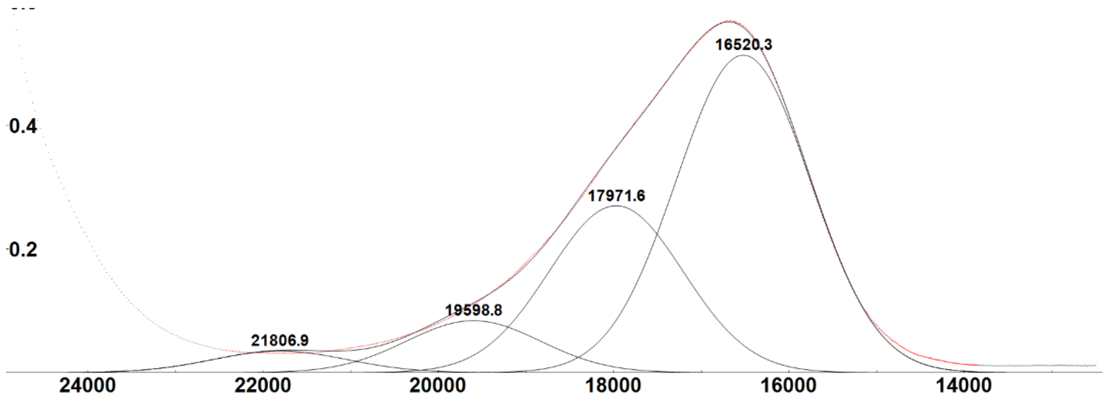

Figure S60. Spectral deconvolution of the UV-vis spectrum of **5’** in toluene represented as wavenumbers [cm^{-1}]. Black: model functions, FWHM = 900 cm^{-1}, red: experimental spectrum.

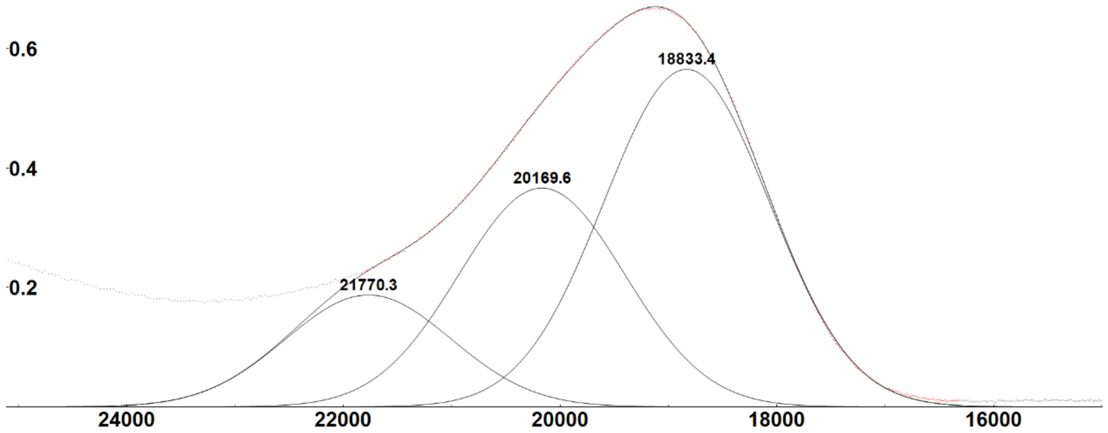

Figure S61. Spectral deconvolution of the UV-vis spectrum of **4’** in toluene represented as wavenumbers [cm^{-1}]. Black: model functions, FWHM = 900 cm^{-1}, red: experimental spectrum.

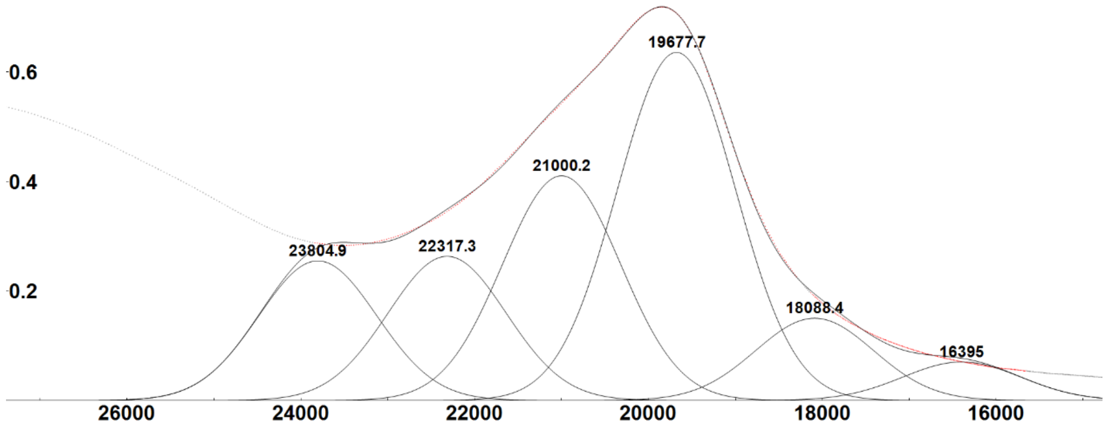

Figure S62. Spectral deconvolution of the UV-vis spectrum of **3’** in toluene represented as wavenumbers [cm^{-1}]. Black: model functions, FWHM = 800 cm^{-1}, red: experimental spectrum.

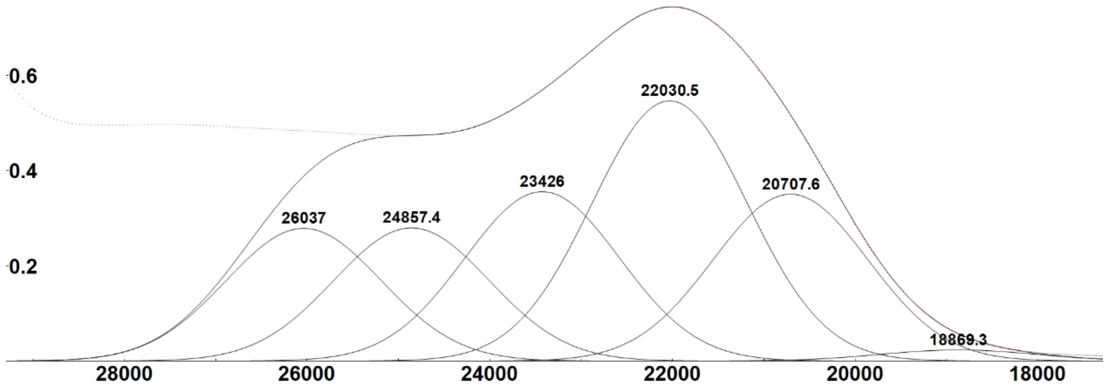

Figure S63. Spectral deconvolution of the UV-vis spectrum of **2'** in toluene represented as wavenumbers [cm^{-1}]. Black: model functions, FWHM = 1000 cm^{-1}, red: experimental spectrum.

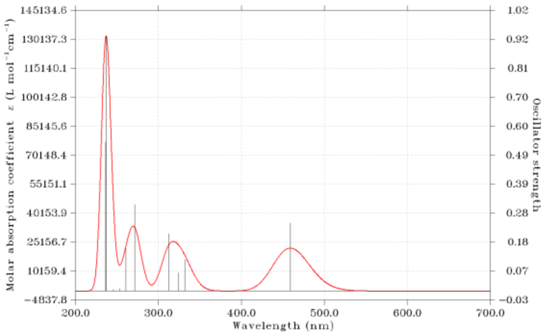

5'

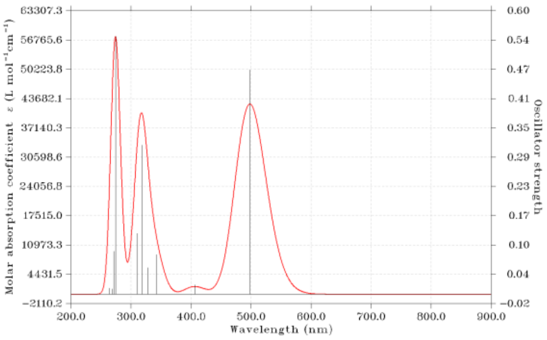

6'

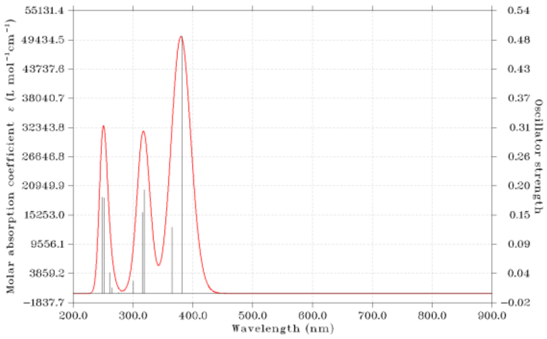

3'

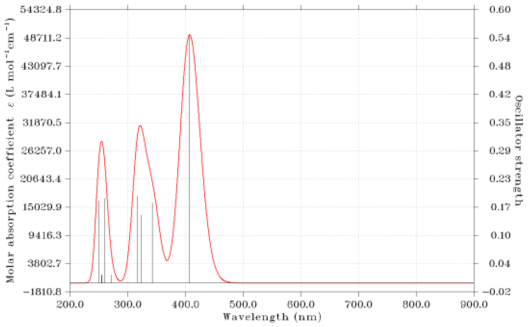

4'

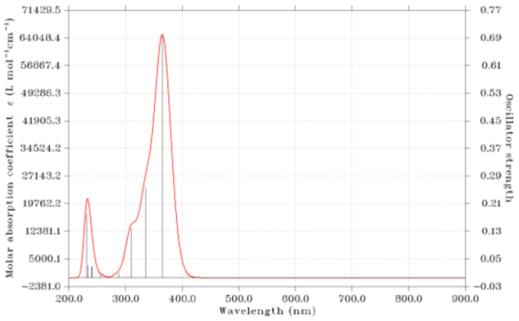

2'

Figure S64. Plot of the spectra obtained from TDDFT calculations. FWHM = 3000 cm^{-1}.

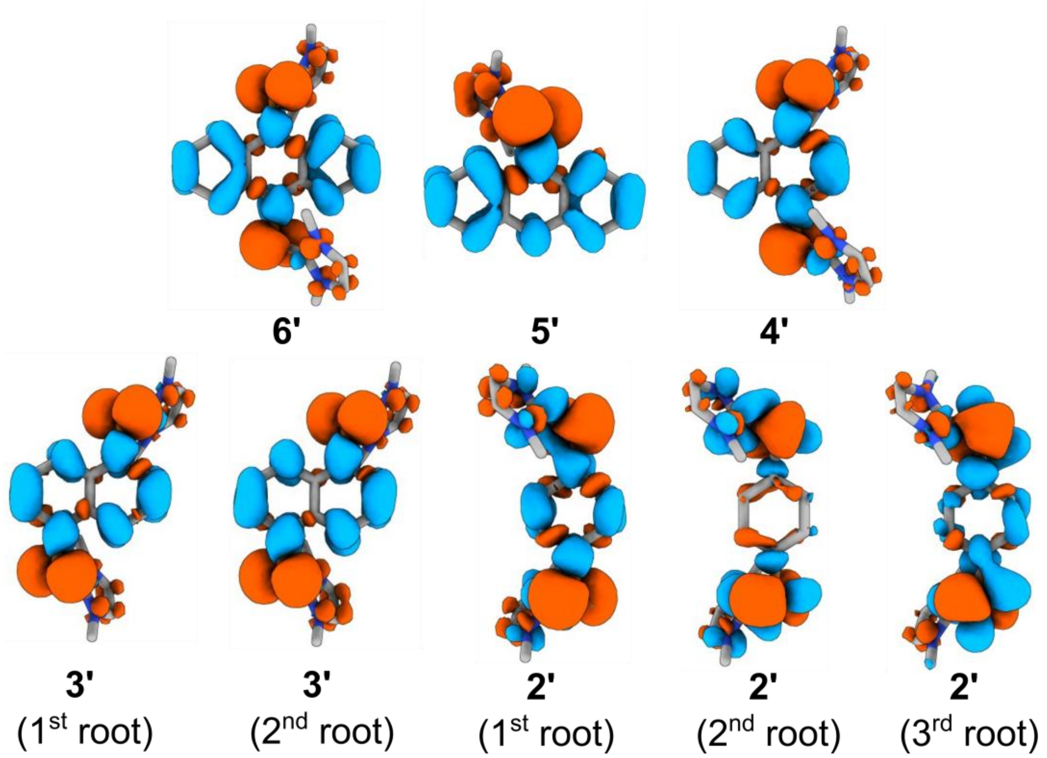

Figure S65. Selected difference densities, transitions proceed from orange to blue. Surface isovalue = 0.001.

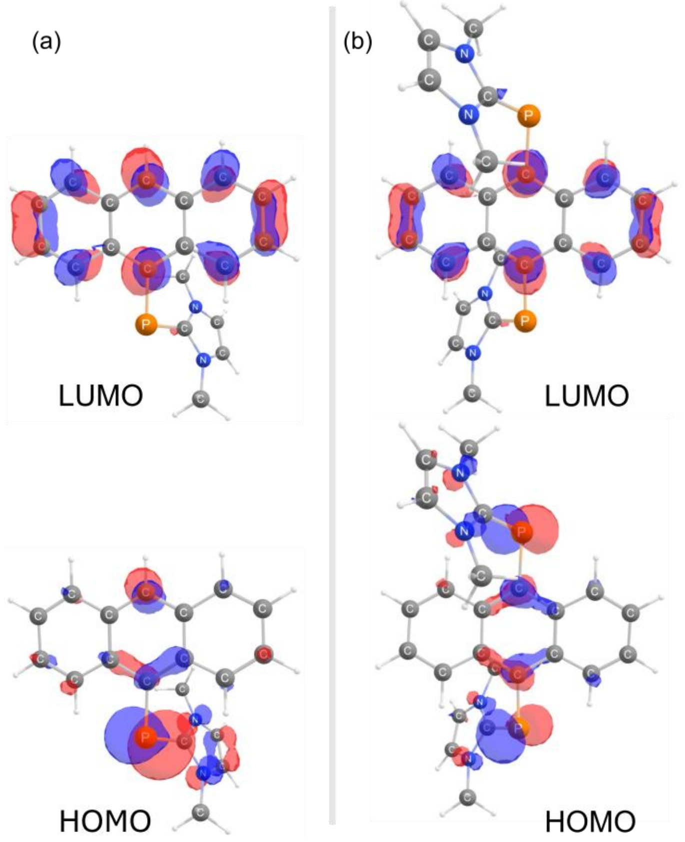

Figure S66. Graphical representations of the calculated frontier orbitals (HOMO & LUMO) for compounds **5** and **6**. Surface isovalue = 0.04.

Table S4. Summary of CASSCF results using localized orbitals as active orbitals.

	**2'**	**3**	**4**	**5**	**6**
Occ. (P)	1.9392, 1.9379	1.9483, 1.9296	1.9392, 1.9385	1.9382	1.9482, 1.9811
Occ. (C)	1.0054, 1.0060, 1.0372, 1.0367, 1.0193	1.0031, 1.0044, 0.9989, 1.0327, 1.0021, 1.0058, 1.0405, 1.0027, 1.0110, 1.0209	1.0200, 1.0198, 1.0387, 1.0375, 1.0003, 1.0003, 0.9957, 1.0072, 0.9956, 1.0072	1.0022, 1.0061, 1.0085, 0.9877, 1.0020, 0.9918, 1.0138, 1.0301, 0.9959, 1.0107, 1.0042, 0.9923, 1.0104, 1.0060	1.0097, 0.9918, 1.0118, 0.9940, 0.9907, 0.9944, 1.0448, 1.0244, 1.0044, 0.9985, 0.9905, 0.9979, 1.0138, 1.0039

4. References

1 S. Li, E. R. Jira, N. H. Angello, J. Li, H. Yu, Y. Diao, M. D. Burke and C. M. Schroeder, *Nat. Commun.*, 2022, **13**, 2102.
2 A. Tsurusaki, N. Nagahora, T. Sasamori, K. Matsuda, Y. Kanemitsu, Y. Watanabe, Y. Hosoi, Y. Furukawa and N. Tokitoh, *Bull. Chem. Soc. Jpn.*, 2010, **83**, 456–478.
3 T. Schaub, M. Backes and U. Radius, *Organometallics*, 2006, **25**, 4196–4206.
4 M. Muhr, P. Heiß, M. Schütz, R. Bühler, C. Gemel, M. H. Linden, H. B. Linden and R. A. Fischer, *Dalton Trans.*, 2021, **50**, 9031–9036.
5 O. Back, M. Henry-Ellinger, C. D. Martin, D. Martin and G. Bertrand, *Angew. Chem. Int. Ed.*, 2013, **52**, 2939–2943.
6 S. A. Reiter, S. D. Nogai and H. Schmidbaur, *Z. Für Naturforschung B*, 2005, **60**, 511–519.
7 G. R. Fulmer, A. J. M. Miller, N. H. Sherden, H. E. Gottlieb, A. Nudelman, B. M. Stoltz, J. E. Bercaw and K. I. Goldberg, *Organometallics*, 2010, **29**, 2176–2179.
8 E. Fišerová and M. Kubala, *J. Lumin.*, 2012, **132**, 2059–2064.
9 G. M. Sheldrick, SHELXL-97, Program for Crystal Structure Refinement, Göttingen, 1997.
10 G. M. Sheldrick, *Acta Crystallogr. Sect. Found. Adv.*, 2015, **71**, 3–8.
11 G. M. Sheldrick, *Acta Crystallogr. Sect. C Struct. Chem.*, 2015, **71**, 3–8.
12 F. Neese, F. Wennmohs, U. Becker and C. Riplinger, *J. Chem. Phys.*, 2020, **152**, 224108.
13 F. Neese, *Wiley Interdiscip. Rev. Comput. Mol. Sci.*, 2018, **8**, e1327.
14 R. A. Kendall and H. A. Früchtl, *Theor. Chem. Acc.*, 1997, **97**, 158–163.
15 F. Neese, F. Wennmohs, A. Hansen and U. Becker, *Chem. Phys.*, 2009, **356**, 98–109.
16 S. Grimme, J. Antony, S. Ehrlich and H. Krieg, *J. Chem. Phys.*, 2010, **132**, 154104.
17 S. Grimme, S. Ehrlich and L. Goerigk, *J. Comput. Chem.*, 2011, **32**, 1456–1465.
18 J. P. Perdew, *Phys. Rev. B*, 1986, **33**, 8822–8824.
19 A. D. Becke, *Phys. Rev. A*, 1988, **38**, 3098–3100.
20 F. Weigend and R. Ahlrichs, *Phys. Chem. Chem. Phys.*, 2005, **7**, 3297–3305.
21 Y.-S. Lin, G.-D. Li, S.-P. Mao and J.-D. Chai, *J. Chem. Theory Comput.*, 2013, **9**, 263–272.
22 M. Cossi, N. Rega, G. Scalmani and V. Barone, *J. Comput. Chem.*, 2003, **24**, 669–681.
